# Factors Affecting the Delivery, Access, and Use of Interventions to Prevent Malaria in Pregnancy in Sub-Saharan Africa: A Systematic Review and Meta-Analysis

**DOI:** 10.1371/journal.pmed.1001488

**Published:** 2013-07-23

**Authors:** Jenny Hill, Jenna Hoyt, Anna Maria van Eijk, Lauren D'Mello-Guyett, Feiko O. ter Kuile, Rick Steketee, Helen Smith, Jayne Webster

**Affiliations:** 1 Department of Clinical Sciences, Liverpool School of Tropical Medicine, Liverpool, United Kingdom; 2 Malaria Control Program, PATH, Seattle, Washington, United States of America; 3 Department of International Public Health, Liverpool School of Tropical Medicine, United Kingdom; 4 Disease Control Department, London School of Hygiene and Tropical Medicine, London, United Kingdom; Kenya Medical Research Institute - Wellcome Trust Research Programme, Kenya

## Abstract

**Background:**

Malaria in pregnancy has important consequences for mother and baby. Coverage with the World Health Organization–recommended prevention strategy for pregnant women in sub-Saharan Africa of intermittent preventive treatment in pregnancy (IPTp) and insecticide-treated nets (ITNs) is low. We conducted a systematic review to explore factors affecting delivery, access, and use of IPTp and ITNs among healthcare providers and women.

**Methods and Results:**

We searched the Malaria in Pregnancy Library and Global Health Database from 1 January 1990 to 23 April 2013, without language restriction. Data extraction was performed by two investigators independently, and data was appraised for quality and content. Data on barriers and facilitators, and the effect of interventions, were explored using content analysis and narrative synthesis. We conducted a meta-analysis of determinants of IPTp and ITN uptake using random effects models, and performed subgroup analysis to evaluate consistency across interventions and study populations, countries, and enrolment sites. We did not perform a meta-ethnography of qualitative data.

Ninety-eight articles were included, of which 20 were intervention studies. Key barriers to the provision of IPTp and ITNs were unclear policy and guidance on IPTp; general healthcare system issues, such as stockouts and user fees; health facility issues stemming from poor organisation, leading to poor quality of care; poor healthcare provider performance, including confusion over the timing of each IPTp dose; and women's poor antenatal attendance, affecting IPTp uptake. Key determinants of IPTp coverage were education, knowledge about malaria/IPTp, socio-economic status, parity, and number and timing of antenatal clinic visits. Key determinants of ITN coverage were employment status, education, knowledge about malaria/ITNs, age, and marital status. Predictors showed regional variations.

**Conclusions:**

Delivery of ITNs through antenatal clinics presents fewer problems than delivery of IPTp. Many obstacles to IPTp delivery are relatively simple barriers that could be resolved in the short term. Other barriers are more entrenched within the overall healthcare system or socio-economic/cultural contexts, and will require medium- to long-term strategies.

*Please see later in the article for the Editors' Summary*

## Abstract 2

Jenny Hill and colleagues conduct a systematic review and meta-analysis of qualitative, quantitative, and mixed methods studies to explore the factors that affect the delivery, access, and use of interventions to prevent malaria in pregnant women in sub-Saharan Africa.

*Please see later in the article for the Editors' Summary*

## Editors' Summary

**Background:**

Half the world's population is at risk of malaria, a mosquito-borne parasite that kills a million people every year. Most of these deaths occur among young children in sub-Saharan Africa, but pregnant women and their unborn babies are also vulnerable to malaria. Infection with malaria during pregnancy can cause maternal death, severe maternal anemia, miscarriages, and pre-term and low-birth-weight babies. Malaria in pregnancy is responsible for about 100,000 babies and 10,000 women dying every year but is preventable by simple, inexpensive interventions that have been available for many years. The World Health Organization recommends a three-pronged approach to the prevention of malaria in pregnancy in areas with stable malaria transmission in Africa—delivery of the antimalarial drug sulfadoxine-pyrimethamine to pregnant women during antenatal clinic visits (intermittent preventative treatment in pregnancy; IPTp), the use of insecticide-treated bed nets (ITNs) to protect pregnant women from the bites of infected mosquitoes, and effective diagnosis and case management of pregnant women with malarial illness.

**Why Was This Study Done?:**

Coverage with this prevention strategy is currently very low. Recent survey data from sub-Saharan African countries suggest that only about a quarter of pregnant women receive two doses of IPTp and only about a third use ITNs. To improve coverage, public health experts need to understand why coverage is so low, and they need to know the factors (determinants) that are associated with the uptake of IPTp and ITNs. In this systematic review and meta-analysis of qualitative, quantitative, and mixed methods studies, the researchers explore the factors that affect delivery, access, and use of IPTp and ITNs among pregnant women in sub-Saharan Africa. A systematic review uses predefined criteria to identify all the research on a given topic. Meta-analysis is a statistical method for combining the results of several studies. Qualitative studies collect non-quantitative data such as reasons for not accepting an intervention, whereas quantitative studies collect numerical data such as the proportion of a population accepting an intervention.

**What Did the Researchers Do and Find?:**

The researchers' search of the Malaria in Pregnancy Library (a resource maintained by the Malaria in Pregnancy Consortium) and the Global Health Database identified 98 studies that provided data on barriers to and determinants of IPTp and ITN uptake and/or data on interventions designed to increase IPTp and ITN uptake. The researchers explored these data using content analysis (a research methodology that examines words and phrases within texts) and narrative synthesis (a method for summarizing results drawn from several qualitative studies). Key barriers to the provision and uptake of IPTp and ITNs included unclear policy and guidance on IPTp, general healthcare system issues such as drug shortages, healthcare facility issues such as unavailability of water for the provision of IPTp by directly observed therapy, poor healthcare provider performance such as confusion about the timing of IPTp doses, and the delayed antenatal care-seeking practices of pregnant women. The researchers' meta-analysis identified education, knowledge about malaria, socio-economic status, number and timing of antenatal clinic visits, and number of pregnancies as key determinants of IPTp uptake, and employment status, education, knowledge, age, and marital status as key determinants of coverage of ITN use. So, for example, highly educated women were more likely to receive IPTp or ITNs than poorly educated women.

**What Do These Findings Mean?:**

These findings identify key interacting barriers to access, delivery, and use of IPTp and ITNs in sub-Saharan Africa and show that these barriers are relatively consistent across countries. Moreover, they suggest that there are fewer barriers to the delivery of ITNs through antenatal clinics than to the delivery of IPTp. Importantly, some of the barriers to IPTp uptake can be resolved in the short term (for example, simplification of country policies and guidance on IPTp might increase its uptake), but barriers to uptake that are entrenched within the overall healthcare system will only be resolved with medium- to long-term strategies that aim to improve the quality of antenatal services and to encourage antenatal clinic use among women. Overall, this analysis provides a checklist of factors that policy-makers involved in national malaria programs may be able to use to help them decide which interventions to prioritize. However, the researchers warn, multi-country studies are nevertheless urgently needed to evaluate targeted or multifaceted interventions designed to increase delivery and uptake of IPTp and ITNs, to reduce the adverse consequences of malaria in pregnancy.

**Additional Information:**

Please access these websites via the online version of this summary at http://dx.doi.org/10.1371/journal.pmed.1001488.

- Information is available from the World Health Organization on malaria (in several languages) and on IPTp; the World Malaria Report 2012 provides details of the current global malaria situation
- The US Centers for Disease Control and Prevention also provides information on malaria and on IPTp; a personal story about malaria in pregnancy is available
- Information is available from the Roll Back Malaria Partnership on all aspects of global malaria control, including information on malaria in pregnancy
- The Malaria in Pregnancy Consortium is undertaking research into the prevention and treatment of malaria in pregnancy
- MedlinePlus provides links to additional information on malaria (in English and Spanish)

## Introduction

Malaria in pregnancy can have important consequences for the mother, foetus, and newborn child, yet the harmful effects are preventable [1]. The adverse outcomes of malaria in pregnancy can be substantially reduced by interventions that have been available for over two decades [2]–[4] and that are inexpensive and cost-effective [5]. Access to and use of these interventions by pregnant women is, however, extremely low, representing a failure of the public health community.

In areas of stable malaria transmission in Africa the World Health Organization (WHO) recommends a package of intermittent preventive treatment in pregnancy (IPTp) with sulphadoxine–pyrimethamine (SP) and use of insecticide-treated nets (ITNs), together with effective case management of clinical malaria and anaemia [6]. IPTp consists of two doses of SP taken 1 mo apart commencing in the second trimester [7],[8]. Both IPTp and ITNs are commonly delivered through antenatal clinics (ANCs) through collaboration between malaria and reproductive health programmes. The Roll Back Malaria Partnership aims to ensure that all pregnant women receive IPTp and at least 80% of people at risk from malaria in areas of high-intensity transmission use ITNs by 2010 [9], with even more ambitious targets of 100% for both interventions by 2015 [10].

Achievement of high coverage of these preventive interventions among pregnant women remains elusive for many countries in sub-Saharan Africa [11],[12]. A recent review of national survey data shows that in 27 countries with survey data between the years 2009 and 2011, the median coverage of two doses of SP was 24.5% (range 7.3%–69.4%), even though the median coverage for at least two ANC visits was 84.6% (range 49.7%–96.9%, 22 countries, 2003–2011) (A. M. van Eijk, personal communication), representing substantial missed opportunities at ANCs. Despite the call for universal ITN coverage [13] and all 45 malaria-endemic countries having a policy of providing ITNs to pregnant women, the median use of an ITN the previous night among pregnant women in 37 countries from survey data for the years 2009–2011 was 35.3% (range 5.2%–75.5%) (A. M. van Eijk, personal communication). According to a Countdown to 2015 report, in 20 countries with data, IPTp and ITNs, together with case management of malaria during pregnancy, have the lowest coverage among all the interventions delivered to pregnant women at ANCs [14].

Evidence on the determinants of coverage and reasons for the failure in delivery and uptake of IPTp and ITNs from qualitative [15] and quantitative studies is currently disparate, in addition to which, many relevant reviews are now outdated [5],[16]–[18]. We therefore undertook a systematic review to update the evidence and to integrate findings from three separate syntheses of studies on (1) barriers to achieving high coverage, (2) determinants of uptake, and (3) interventions to increase coverage. We then explored the extent to which the intervention studies have addressed known barriers and determinants, and identified critical gaps in the knowledge required for the formulation of effective strategies. The review was restricted to sub-Saharan Africa as the only malaria-endemic region with a specific WHO strategy for the prevention of malaria in pregnancy, which includes both IPTp with SP and ITNs.

## Methods

### Search Strategy

We performed a systematic and comprehensive literature search of electronic databases on 23 April 2013, including the Malaria in Pregnancy Library (http://library.mip-consortium.org; updated 20 April 2013) and the Global Health Database [19], and a search of bibliographies of retrieved articles. The Malaria in Pregnancy Library contains peer-reviewed published and unpublished literature compiled from 40 sources including PubMed, the Global Health Library, Google Scholar, Lilacs (Latin American and Caribbean Health Sciences Literature), Popline, the ProQuest Digital Dissertations and Theses database, Web of Knowledge, WorldCat, and registers of trials and studies [20]. A full account of the search terms used is presented in Table S1.

### Study Inclusion Criteria and Analysis Strategy

Titles and abstracts were reviewed independently by two authors (J. Hill and J. Hoyt/A. M. van Eijk). Studies were eligible for inclusion if they met the following criteria: (1) reported an original research study; (2) addressed barriers to, facilitators of, or determinants of the delivery or uptake of IPTp and/or ITNs in pregnancy, or evaluated the impact of an intervention to increase the coverage of IPTp and/or ITNs in pregnancy; (3) were published between 1 January 1990 and 23 April 2013; and (4) were conducted in sub-Saharan Africa. No restrictions were placed on publication language or study design, i.e., quantitative, qualitative, and mixed methods studies were included, and both peer-reviewed papers and grey literature were included. Studies meeting the inclusion criteria were grouped according to whether their content addressed (1) barriers or facilitators, (2) determinants, and/or (3) evaluation of intervention(s); some studies contributed to more than one of these content groups (Figure 2). Studies with content on barriers or facilitators and/or determinants were then further categorised into studies exploring factors among pregnant women, healthcare providers, or both. Studies with content on delivery interventions were categorised by intervention, i.e., IPTp, ITNs, or both. The kappa statistic was used to measure the chance-adjusted inter-rater agreement for eligibility.

### Data Extraction

Two authors extracted data and appraised the quality and content of included studies. J. Hill and J. Hoyt/A. M. van Eijk extracted quantitative and qualitative data on barriers and facilitators from quantitative, qualitative, and mixed methods studies using pre-existing themes used by the authors of the included studies, which were stratified according to whether the views or perspectives were those of pregnant women or healthcare providers; the views or perspectives mainly comprised self-reported information but also observed data. The barrier and facilitator themes were then divided into four predetermined categories adapted from the literature [21],[22] for pregnant or postpartum women (Box 1) and for healthcare providers (Box 2). Because facilitators uniformly reflected the converse of the barriers, we report only the barriers (Table S4). A. M. van Eijk and J. Hoyt/L. D'Mello-Guyett extracted quantitative data from quantitative and mixed methods studies that explored the determinants of receipt of one or two doses of IPTp and ITN ownership and use, henceforth referred to as “determinants”. J. Hill and J. Hoyt/L. D'Mello-Guyett extracted quantitative, qualitative, and descriptive data from the studies evaluating delivery strategies for IPTp and/or ITNs according to the type of delivery intervention, e.g., promotion, training, or type of delivery mechanism.

**Box 1. Barriers from the Women's Perspective by Level**

**Individual level:** factors related to a woman's knowledge, thoughts, beliefs, actions and behaviour, pregnancy, and health status

**Social/cultural/household level:** factors related to a woman's economic and social position, household factors including gender roles, societal and cultural norms and traditions, and religious practices

**Environmental level:** factors related to seasonality of malaria, weather conditions, physical access, and transportation

**Healthcare system level:** factors related to the various components and quality of the healthcare system, such as staff attitudes or performance, medication, service provision, and user fees

**Box 2. Barriers from the Healthcare Provider Perspective by Level**

**Individual level:** factors related to the knowledge, attitudes, and performance of individual healthcare providers

**Organisational level:** factors related to the operation of the health facility unit, such as management, staff rosters/rotation, and services

**Healthcare system level:** factors that are dependent on higher levels of the healthcare system related to the various components and quality of services, such as supply of drugs or ITNs, development and dissemination of policy guidelines, training and supervision of staff, and imposition of user fees

**Non-Healthcare system:** macro-level factors external to the healthcare system such as media, water supply, side effects of medications, and women's practices

Two authors (J. Hill and J. Hoyt/L. D'Mello-Guyett) assessed the quality of reporting of individual studies using a checklist of criteria developed a priori based on criteria and methods described in the literature. For observational quantitative studies the criteria of reporting were randomised sample selection, multivariate analysis, and minimising bias through study design and analysis [23],[24]. For qualitative studies the criteria were the extent to which the authors described the sampling strategy, the effects of reflexivity, and methods to ensure reliability and validity [25],[26]. For mixed methods studies, the following reporting criteria were used: justification of mixed methods, clearly described sampling strategy, clear reporting of methods for the qualitative component, analysis strategy, multivariate analyses, minimising bias, and integration of qualitative and quantitative findings [27],[28]. For intervention studies, reporting criteria were presence/type of control, steps to reduce bias, and the extent to which authors described confounding, loss to follow-up, and external validity [29]. No studies were excluded on the basis of quality.

### Data Synthesis

Barriers and facilitators were described and explored using content analysis and narrative synthesis of qualitative and quantitative data. Data from the pregnant women's perspective were synthesised across four levels (individual, household/social/cultural, healthcare system, and environmental) and assessed in relation to receipt of IPTp, ITN ownership, and ITN use. Similarly, data from the healthcare provider perspective were synthesised across four levels (individual, organisational, healthcare system and non-healthcare system) and assessed in relation to the delivery of IPTp and ITNs in the ANC setting.

The intervention studies were grouped into common strategies and explored using a narrative synthesis to summarise each intervention and to compare and contrast findings between studies evaluating similar strategies for scaling up one or both malaria interventions.

### Statistical Analysis

We conducted a meta-analysis of data on determinants using Stata version 12 (StataCorp) and Comprehensive Meta-Analysis (Biostat, http://www.meta-analysis.com/). Summary odds ratios (ORs) were calculated using random effects models based on the approach of DerSimonian and Laird [30]. Data were extracted from studies using the following hierarchy based on availability: raw data (numerators and denominators); computed unadjusted ORs, computed adjusted ORs. The use of adjusted (by multivariate analysis) or cluster-adjusted ORs as provided by the studies is indicated in the meta-analysis forest plots. If studies presented results for both “1+ doses” and “2+ doses” of IPTp, only the data for “2+ doses” was used. We conducted sub-group analysis and considered the following factors for IPTp: number of SP doses (1+ or 2+), location of enrolment (community or clinic), study population (postpartum women, a mixed population of postpartum and pregnant women, or pregnant women only), and study country. The subgroup analysis for ITNs considered location of enrolment (community or health facility), study population (postpartum women, a mixed population of postpartum and pregnant women, or pregnant women only), study country, and—for ITNs—type of net (ITN or untreated net) and definition of net use (last night or during pregnancy). Sensitivity analysis was conducted to assess the potential effect of study quality on the examined associations. We assigned studies a score based on the quality assessment, and studies that failed to report on three or more quality criteria scored as low-to-moderate quality. The *I* ^2^ and 95% CI were used to quantify heterogeneity [31].

### Synthesis across the Barriers, Determinants, and Intervention Studies

We compared identified barriers with the determinants identified in the meta-analysis and aligned them with the intervention studies. The barriers were first collapsed into a limited number of key categories using a coding template, and the implications for intervention for each category of barriers were described. We then matched the proposed interventions derived from the barrier studies against the intervention studies included in the review to assess the extent to which the intervention studies addressed the barriers identified in the observational studies.

## Results

### Study Selection and Characteristics

**Figure 1:**
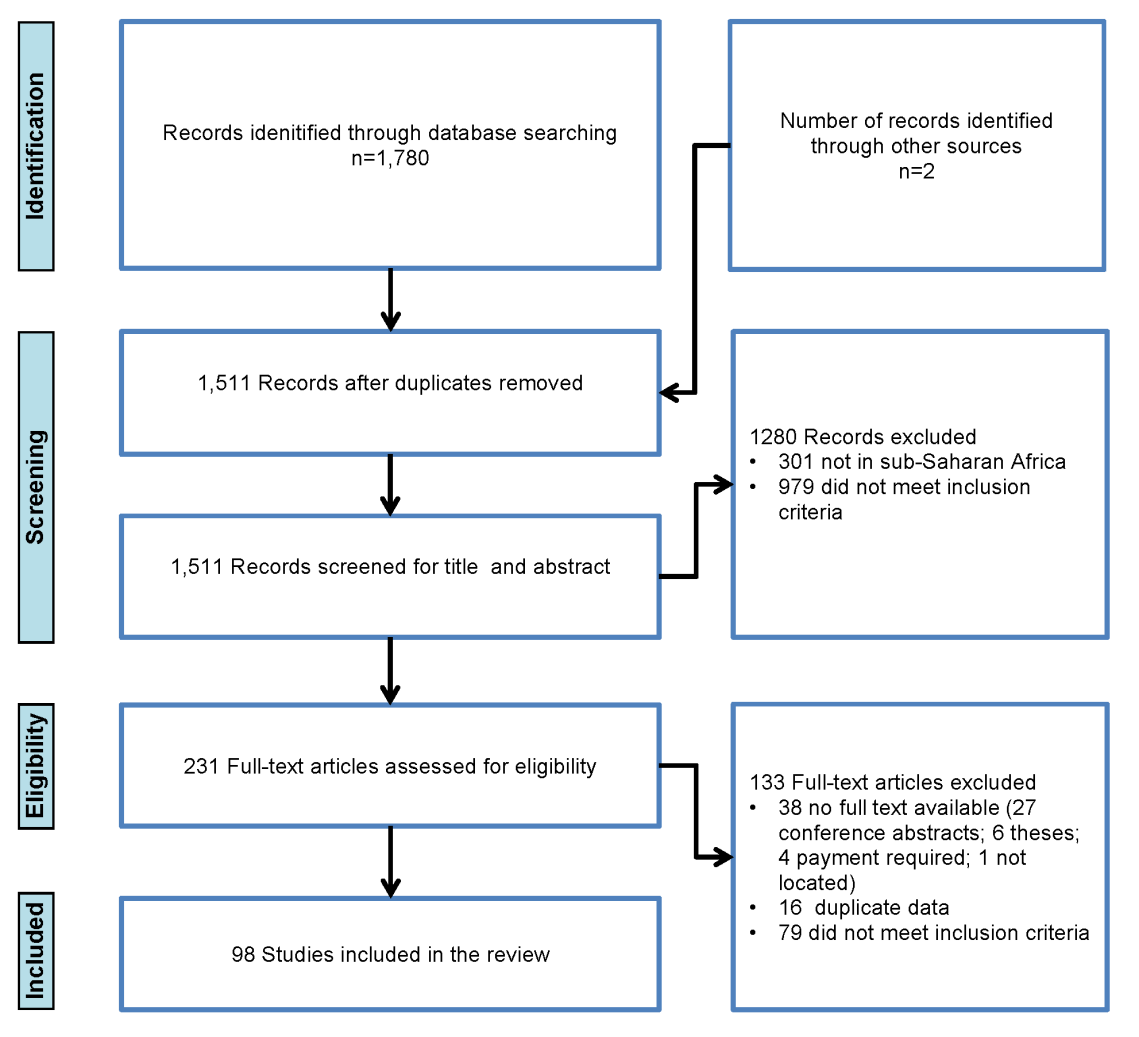
Flowchart of studies included in the review.

The primary search identified 1,780 citations (1,240 from the Malaria in Pregnancy Library, 540 from the Global Health Database, and two from bibliographies and authors), from which 271 duplicates were removed (Figure 1). From the remaining 1,511, 1,280 articles were excluded on the basis of abstracts. Of 231 full-text articles reviewed, 133 were excluded as they did not meet the inclusion criteria, the full text was not available, or they contained duplicate data, leaving 98 included articles. There was close agreement between reviewers on the included studies (kappa score of 0.86).

**Figure 2:**
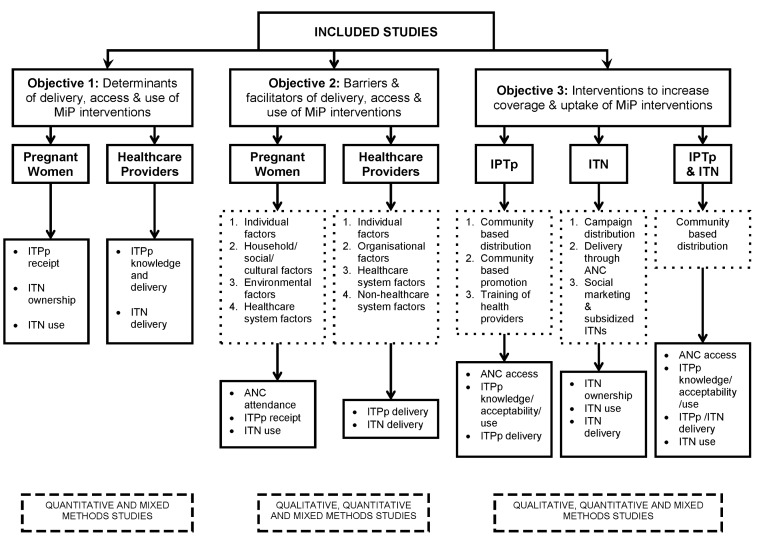
Analysis strategy. MiP, malaria in pregnancy.

Of the 98 included studies, 81 contributed data on barriers and determinants (Table 1), and 20 studies contributed data on interventions that aimed to increase coverage and uptake of IPTp (Table 2) or ITNs (Table 3). One study did not contain data in a usable format for the meta-analysis [32]. The key characteristics of the barrier and determinant studies and of the intervention studies are provided in Table S2.

**Table 1:** Data extracted for barriers and determinants by study.

Study	IPTp		ITN	
**Facility-based surveys**	**Barriers**	**Determinants**	**Barriers**	**Determinants**
Akaba 2013 [34]	√	√	√	√
De Allegri 2013 [82]		√	√	
Aluko 2012 [71]			√	√
Amoran 2012a [35]	√	√		
Amoran 2012b [72]			√	√
Arulogun 2012 [55]	√			
Bouyou-Akotet 2013 [113]		√		
Diala 2012 [40]	√			
Iliyasu 2012 [36]	√	√		
Mubyazi 2012 [63]	√			
Mutagonda 2012 [43]	√			
Namusoke 2012 [59]		√		
Onoka 2012a [37]	√	√		
Onoka 2012b [114]	√			
Onwujekwe 2012 [61]	√			
d'Almeida 2011 [115]		√		
Donkor 2011 [48]	√			
Manirakiza 2011 [116]		√		√
Napoleon 2011 [117]		√		√
Nduka 2011 [118]		√		
Okonta 2011 [73]			√	√
Olajide 2011 [74]			√	√
Tutu 2011 [119]	√			
Smith Paintain 2011 [64]	√			
Gross 2011 [33]	√			
Ambrose 2011 [77]			√	
Sande 2010 [45]	√	√		
Antwi 2010 [53]	√	√		
Mubyazi 2010 [52]	√		√	
Smith 2010 [47]	√			
Karunamoorthi 2010 [67]			√	√
Wagbatsoma 2010 [120]			√	
Akinleye 2009 [121]	√			
Takem 2009 [122]		√		
Klebi 2009 [123]		√		
Musa 2009 [69]			√	
Njoroge 2009 [65]			√	√
Adjei 2009 [49]	√	√		
Mubyazi 2008 [18]	√			
Pettifor 2008 [76]			√	√
Anders 2008 [56]	√			
Onyeaso 2007 [60]	√		√	
Mnyika 2006 [124]				√
Launiala 2007 [44]	√			
Brentlinger 2007 [62]	√		√	
Kweku 2007 [83]			√	
Van Geertruyden 2005 [78]			√	√
Gates Malaria Partnership 2005 [39]	√			
Mubyazi 2005 [41]	√			
Nganda 2004 [125]		√	√	√
Ashwood-Smith 2002 [54]	√			
**Community-based surveys**				
Hill 2013 [126]		√		√
Ankomah 2012 [80]			√	√
Ansah-Ofei 2011 [46]	√			
Auta 2012 [81]			√	√
Zere 2012 [127]	√	√		√
Faye 2011 [128]		√		
O'Meara 2011 [85]	√			
Ndyomugyenyi 2010 [50]	√	√		
Grietens 2010 [51]	√			
Sangare 2010a [57]	√	√		
Mbonye 2010 [129]		√		
Sangare 2010b [70]			√	√
Beiersmann 2010 [84]			√	
Acquah 2009 [130]	√			
Brabin 2009 [42]	√			
Gies 2009 [90]		√		
Gikandi 2008 [131]		√		√
Marchant 2008 [58]	√	√		
Belay 2008 [79]			√	√
Hassan 2008 [132]				√
Kiwuwa 2008 [133]		√		√
Ouma 2007 [91]		√		
PSI Burundi 2006 [134]				√
PSI Rwanda 2006 [135]				√
PSI Zambia 2006 [136]			√	
Mbonye 2006a [38]	√			
Mbonye 2006b [66]			√	
van Eijk 2005 [68]		√	√	√
Guyatt 2004 [137]		√	√	√
Marchant 2002 [75]			√	√
**Summary total**	**38**	**31**	**28**	**27**

**Table 2:** Evaluation of interventions aimed at increasing coverage of IPTp (six studies).

Study/Measure	Description (Country)	Baseline			Point of Evaluation			
		Intervention (Percent)	Control (Percent)	*p*-Value	Intervention (Percent)	Control (Percent)	*p*-Value^a^	*p*-Value^b^
**Msyamboza 2009 ** [**86**]	IPTp delivered by community health workers (Malawi)							
IPTp 2+		36/87 (41.4)	47/107 (43.9)	0.77	663/912 (72.7)	412/897 (45.9)	<0.001	<0.001
ANC 2+		76/87 (87.3)	103/107 (96.3)	0.03	586/888 (66.0)	831/895 (92.9)	<0.001	<0.001
**Mbonye 2007 ** [**87**]	IPTp delivered by community resource persons, sensitisation campaigns (Uganda)							
IPTp 2+^c^					1,404/2,081 (67.5)	281/704 (39.9)	<0.001	
ANC 2+^d^					948/983 (96.4)	240/247 (97.2)	0.70	
ANC 4+^d^					558/983 (56.8)	188/247 (76.1)	<0.001	
**Ndyomugyenyi 2009 ** [**88**]	IPTp delivered by community-directed drug distributors for onchocerciasis control (Uganda)							
IPTp 2+		161/317 (50.8)	152/310 (49.0)	0.66	424/473 (89.6)	237/453 (52.3)	<0.001	<0.001
ANC 2+					429/473 (90.7)	364/453 (80.4)	<0.001	
ANC 4+		89/317 (28.1)	77/310 (24.8)	0.36	206/473 (43.6)	90/453 (19.9)	<0.001	<0.001
**Okeibunor 2011 ** [**89**]	IPTp (and ITNs) delivered by community-directed distributors (Nigeria)							
IPTp 2+		66/711 (9.3)	35/563 (6.2)	0.05	66%	27%		<0.01^e^
ANC 1+		489/711 (68.8)	283/563 (50.0)	<0.001	90%	72%		<0.01^e^
**Gies 2009 ** [**90**] ** (G1/G2)**	Community-based promotional activities on IPTp and antenatal care by trained community promoters (Burkina Faso)							
IPTp 2+					518/721 (71.8)	389/793 (49.1)	<0.001	
ANC 2+					644/721 (89.3)	1,144/1,519 (75.3)	<0.001	
ANC 4+					188/721 (26.1)	246/1,519 (16.2)	<0.001	
1st ANC visit in 1st/2nd trim^d^					552/679 (81.3)	961/1,365 (70.4)	<0.001	
**Ouma 2007** f [**91**]	Training of health facility staff in one region on IPTp and focussed antenatal care (Kenya)							
IPTp 2+		22/312 (7.1)	20/302 (6.6)	0.87	99/268 (36.9)	48/440 (10.9)	<0.001	<0.001
ANC 2+		274/319 (85.9)	251/316 (79.4)	0.06	201/272 (73.9)	323/452 (71.5)	0.49	<0.001
1st ANC visit in 1st/2nd trim		235/319 (73.7)	198/316 (62.7)	0.004	166/272 (61.0)	236/452 (52.2)	0.03	0.001

a Comparing intervention and control at point of evaluation.

b Comparing baseline and point of evaluation for intervention.

c Denominator for IPTp 2+: women who have received already one SP dose.

d Denominator: women with at least one ANC visit.

e Analysis adjusted for clustering.

f Information from article enhanced by supplemental data from authors.

G1, primigravidae; G2, secundigravidae; trim, trimester of pregnancy.

**Table 3:** Evaluation of interventions aimed at increasing coverage of ITNs (15 studies).

Type of Distribution	Study/Measure	Description (Country)	Baseline			Point of Evaluation		
			Intervention (Percent)	Control (Percent)	*p*-Value	Intervention (Percent)	Control (Percent)	*p*-Value^a^
**Campaign**	**Okeibunor 2011** b [**89**]	Community distribution of free ITNs to pregnant women (Nigeria)						
	ITN use last night		128/711 (18.0)	48/563 (8.5)	<0.001	28%	10%	0.12
	**Thwing 2011** b [**92**]	Community distribution of voucher for free ITN to households with children under 5 y (Senegal)						
	ITN use last night		28.5%			49.2%		—c
	**Ahmed 2010 ** [**93**]	Community distribution of subsidised ITNs to poor households (Uganda)						
	ITN use last night (peri-urban)					324/1306 (24.8)		0.13^d^
	ITN use last night (rural)					1,340/4,983 (26.9)		
	**Khatib 2008** e [**94**]	ITN coverage among infants by delivery channel of net—ANC voucher versus under five vaccination (Tanzania)						
	ITN use by infant							
	ITN use last night (ANC voucher)					175/422 (41.5)		-
	ITN use last night (vaccination campaign)					114/422 (27.0)		
	ITN use last night (commercial market)	Campaign versus commercial market (Tanzania)				101/422 (24.0)		
	**Mbonye 2007 ** [**87**]	Malaria prevention promotion by community resource persons, sensitisation campaigns (Uganda)						
	Use of ITN in pregnancy		160/2,078 (7.7)	85/703 (12.1)	<0.001	211/1,416 (14.9)	64/259 (24.7)	<0.001
**ANC**	**Guyatt 2003** f [**99**]	Free ITN distribution through ANC (Kenya)						
	ITN use in pregnancy (high transmission)					93/111 (83.8)		-
	ITN use in pregnancy (low transmission)					73/126 (57.9)		-
	**Pettifor 2009 ** [**98**]	Free ITN distribution through ANC (DRC)						
	ITN use last night		82/326 (25.2)			258/326 (79.1)		<0.001
	**Hanson 2009** b [**101**]	Voucher for subsidised ITN through ANC (Tanzania)						
	ITN use last night		82/772 (10.6)			144/621 (23.2)		<0.001
	**Marchant 2010** b [**100**]	Voucher for subsidised ITN through ANC (Tanzania)						
	ITN use last night (poorest quintile)					10/138 (6.9)		<0.001^g^
	ITN use last night (wealthiest quintile)					54/113 (47.9)		
	**Kweku 2007 ** [**83**]	Voucher for subsidised ITN through ANC (Ghana)						
	Vouchers redeemed (urban)					63.3%		0.009^h^
	Vouchers redeemed (rural)					47.0%		
	**Muller 2008** b [**102**]	Social marketing with/without free ITN distribution through ANC: randomised controlled trial (Burkina Faso)						
	ITN use last night (free ITNs at ANC)		5/72 (6.9)			10/107 (9.3)		
	ITN use last night (no free ITNs at ANC)		5/100 (5.0)			14/105 (13.3)		
**Community-based**	**Nonaka 2012 ** [**103**]	Subsidised ITNs through community health committee to all households irrespective of pregnant women (Niger)						
	ITN use last night					51/64 (76.1)	42/62 (64.6)	0.18
**Social marketing**	**PSI Madagascar 2009 ** [**96**]	Social marketing and subsidised ITNs (Madagascar)						
	ITN use last night		35/311 (11.1)			101/176 (57.6)		<0.001
	**PSI Kenya 2008** i [**95**]	Social marketing and subsidised ITNs (Kenya)						
	ITN use last night					79/177 (44.6)		
	**PSI Burundi 2007 ** [**97**]	Social marketing and subsidised ITNs (Burundi)						
	ITN use last night		142/721 (19.7)			181/611 (29.6)		<0.01

a Comparing intervention and control at point of evaluation.

b Analysis adjusted for clustering.

c Sample sizes not provided.

d Comparing peri-urban and rural women.

e ITN use in infants used as a proxy for ITN use by pregnant women, as women share their sleeping places with their newborns in this setting.

f ITN use during pregnancy among women who had received a free United Nations Children's Fund ITN during 2001 when they were pregnant and did not previously use an ITN.

g Comparison of the poorest quintile versus wealthiest quintile.

h Comparing redemption of vouchers issued in urban versus rural health facilities.

i ITN use last night among households with a pregnant woman in residence.

DRC, Democratic Republic of the Congo.

Of the 48 quantitative studies, the majority of studies were assessed to be of moderate quality (2–3/5; *n* = 24), with the remaining studies equally distributed between low (0–1/5; *n* = 13) and high (5/5; *n* = 11) quality (Table S3). The criterion least commonly reported among these studies was “social desirability minimised”. The eight qualitative studies were assessed to be of moderate-to-high quality (4–6/7), with only one study reporting on saturation and only two studies reporting on reflexivity of the researcher (Table S3). The majority of the 22 mixed methods studies were assessed to be of medium (4–6/9; *n* = 11) to high quality (8–9/10; *n* = 10), with only three studies reporting on use of multivariate analysis (Table S3). Of the 20 intervention studies, the majority were assessed to be of moderate quality (2–3/4; *n* = 17), with four low-quality studies and one high-quality study (4/4) (Table S3).

### Barriers to IPTp Coverage

#### Barriers to receiving IPTp—the perspective of pregnant women

For barriers categorised as individual, many of the barriers to receipt of IPTp reported by women related to their lack of knowledge about IPTp (see Table S4 for content analysis of qualitative and quantitative data on barriers). For example, women were unaware of the benefits of IPTp [33]–[37] or the preventive value of SP [38],[39], why SP was being given [35],[39],[40], and the number of doses, timing, and dose of SP required [35],[36],[40]–[43]. There was also confusion over what drugs were safe to take during pregnancy [35],[36], leading some women to reject all medication [44] or to fear the perceived side effects of SP [36],[41],[45],[46], with women in one study fearing that SP would cause abortions [38]. Women who had had personal experience of the side effects of SP were also deterred from taking IPTp with SP [41],[43],[46]–[48]. Reasons given for not receiving a second dose of IPTp included not returning for a second ANC visit [38],[49],[50] and illness or shyness [39] or low social position [51] leading to delayed ANC attendance. This lack of knowledge of IPTp means that women who take IPTp are placing considerable trust in ANC staff to provide them with safe and beneficial drugs and services [37],[40],[42],[44],[47].

For barriers categorised as household/social/cultural, it is common for women to have to purchase SP or water for taking SP by directly observed therapy (DOT), constituting an important economic barrier to the uptake of IPTp [35]–[38],[43],[52],[53]. Commitments to farming, employment, and childcare were barriers to ANC attendance earlier in pregnancy, resulting in women receiving no or incomplete doses of IPTp [39],[43]. Women often delayed going to an ANC until the pregnancy was advanced (about 7 mo gestation) [40] because their husbands did not give them money for transport [38], presenting a shorter window of opportunity to receive two doses of IPTp. In Nigeria, women reported needing their husbands' support or consent before attending an ANC or before taking any drugs [36],[40].

For barriers categorised as healthcare system–related, women reported that in some health facilities women who could not pay the fees were denied services [52], or they were asked to pay another fee in order to receive the second dose of IPTp [54]. Barriers to receiving SP by DOT were that women had to buy the drug elsewhere, or that they took it home because they needed to eat first or because the nurses told them to [55], or that they were asked to share cups [36]. Among women who did not receive IPTp, a key reason reported in four studies was that it was not offered by ANC staff [35],[37],[46],[50],[56]–[58]. They also reported frequent periodic shortages of SP as a reason for not receiving IPTp [35],[36],[43],[45],[49],[50]. Women in Tanzania reported fines and penalties being imposed by healthcare providers if they started attending an ANC late in pregnancy [52]. Other reasons for not receiving SP were that women were taking iron sulphate and folic acid supplementation [46] or were referred to a laboratory for testing [46].

#### Barriers to delivery of IPTp—the perspective of healthcare providers

Barriers to the delivery of IPTp found at the individual level centred on the knowledge and perceptions of healthcare providers about IPTp using SP (see Table S4 for content analysis of qualitative and quantitative data on barriers). General knowledge regarding the IPTp strategy among healthcare providers was considered poor [18],[37],[39],[55],[60],[61]. Confusion among healthcare providers over the timing and dosing of SP in relation to gestational age was commonly cited [18],[33],[37],[54],[55],[60],[61], in addition to imprecise estimation of gestational age leading to missed SP doses [62], or SP being given to women regardless of guidelines for gestational age [37],[44],[63]. Healthcare providers could not name the major side effects or contraindications of SP in Ghana or Nigeria [49],[53],[55],[64] and often gave SP and iron tablets to women without any explanations or instructions, or instructions were not given in the local language [44]. Healthcare providers were often found to blame pregnant women's behaviour for the poor uptake of IPTp. For example, healthcare providers in Malawi and Nigeria reported that women did not want to take SP on an empty stomach [37],[54], and that late attendance at the ANC [18],[54],[61] or women not returning for subsequent ANC visits [45],[61] was a contributor to low IPTp coverage.

Organisational-level barriers at health facilities were that staff were sometimes too busy to prescribe SP [54] or that cups [18],[37],[41],[62] or drinking water was not available to enable provision of SP by DOT [37],[49],[63]. An important finding was that there can be substantial variation across health facilities in the delivery of IPTp and in the information provided to pregnant women [18].

Many of the barriers to the effective delivery of IPTp reside within higher levels of the healthcare system and/or at the policy level. Guidelines have generally been too complicated, and in some cases there has been conflicting information from different programmes within the ministry of health, as occurred in Tanzania, where two different IPTp guidelines remained in circulation, one in the 2006 Malaria Diagnosis and Treatment guidelines and one in the Focussed Antenatal Care guidelines [33]. Several studies identified that guidelines were not available at the health facilities [33],[39],[49]. This lack of access to appropriate information is exacerbated by lack of effective training and supervision of healthcare providers and lack of quality assurance of IPTp delivery in facilities [18],[37],[39],[63]. In Tanzania, the change in national treatment policy from using SP to using artemisinin combination therapies was poorly managed and led to negative media coverage about SP and loss in confidence in SP among healthcare providers and the general public [18]. Related to the policy context, a pilot IPTp project in Mozambique encountered multiple incompatibilities between the delivery of IPTp and of other antenatal care initiatives (e.g., programmes for control of syphilis, anaemia, and HIV) that had to be overcome [62]. A major barrier identified in several studies was periodic stockouts of SP, sometimes for extended periods [18],[37],[44],[49],[53],[54],[62],[63]. This results in women either being turned away without being given IPTp or being given a prescription to go and buy the drug from a private drug seller [38] or from a pharmacy at another government facility, and represents a serious missed opportunity, as there is no guarantee that the women will buy and take the drug. Practices in private facilities often differ from those in government health facilities: private facilities are more likely to fail to adhere to national guidelines on IPTp delivery, to charge user fees for IPTp [18], or to dispense other malaria drugs requested by pregnant women [41], creating inconsistencies within national programmes. Delivery of IPTp is impeded by a lack of basic facilities, drug shortages, and insufficient training or support and remuneration [63], with either staff being too busy [54] or SP not being available, such that user fees are levied for IPTp even where it is supposed to be provided free [37],[63].

### Barriers to ITN coverage

#### Barriers to ITN uptake and use—the perspective of pregnant women

A common individual-level barrier to use of ITNs was associated with the inconvenience and discomfort of using an ITN (see Table S4 for content analysis of qualitative and quantitative data on barriers). Pregnant women described feeling hot and uncomfortable under the net while sleeping [34],[48],[65]–[74] and the inconvenience of putting it up and taking it down each night [67],[68],[72]–[74]. The belief that the chemicals used to treat the ITNs were harmful to pregnant women and their unborn child was reported as a barrier in studies in Nigeria [74], Ghana [48], Kenya, and Uganda, which led to many women discontinuing use of ITNs [65],[66]. In Nigeria, women did not believe that ITNs prevented malaria [34],[73].

At the household/social/cultural level, the most frequently reported barrier to ITN access for pregnant women was cost [65],[66],[69],[71]–[78]. The studies describing cost as a major barrier were undertaken in countries in east, west, and central Africa and involved both rural and urban populations, suggesting cost is a common barrier to ITN access in many contexts. Women in several studies indicated either a lack of support from their husband [34],[66],[79] or that they were reliant on their husband to purchase the ITN on their behalf [70],[75]. A barrier to adolescents and primigravidae using ITNs was the perception among community members and the pregnant women themselves that these groups were at a low risk of getting malaria [66]. Place of residence [48],[71],[79]–[82],[99], seasonality of use (low in hot weather) [65],[67], and perceptions that there were no mosquitoes in the area [68],[77],[79] were the main environmental barriers.

The main barrier at the healthcare system level cited by pregnant women was the “unavailability” of ITNs [66],[69],[71],[74],[79]. These stockouts can exacerbate the issue of cost in many cases, as women often travel to distribution points to collect the ITN, incurring both direct and indirect costs, only to find the ITNs out of stock [52]. Barriers associated with the distribution of ITN vouchers through ANC services in Tanzania included long travel distances to redeem the vouchers, variation in top-up costs, and the negative attitudes of ANC staff when women return without having redeemed their voucher [52]. Once women obtain an ITN, they do not always use them. The side effects (burning eyes), heat, and restrained mobility were seen to prevent women from using ITNs, according to healthcare providers in Ghana [48].

#### Barriers to delivery of ITNs—the perspective of healthcare providers

Healthcare providers frequently identified insufficient stock to meet the demand as a major barrier [83],[84] (see Table S4 for content analysis of qualitative and quantitative data on barriers). A study in Kenya found that despite pregnant women being eligible for a free ITN at ANC services, households with a pregnant woman who recently attended ANC services were no more likely to own an ITN than households without a pregnant woman [85], suggesting that free distribution programmes are not always effective at reaching their target population. While the delivery of ITNs or vouchers through ANC services provide extra incentive for pregnant women to attend ANC services [52],[84], it was noted that some women do not return to the ANC once they have received their free ITN [84].

### Determinants of IPTp and ITN Uptake and Use among Women: Meta-Analysis

**Figure 3:**
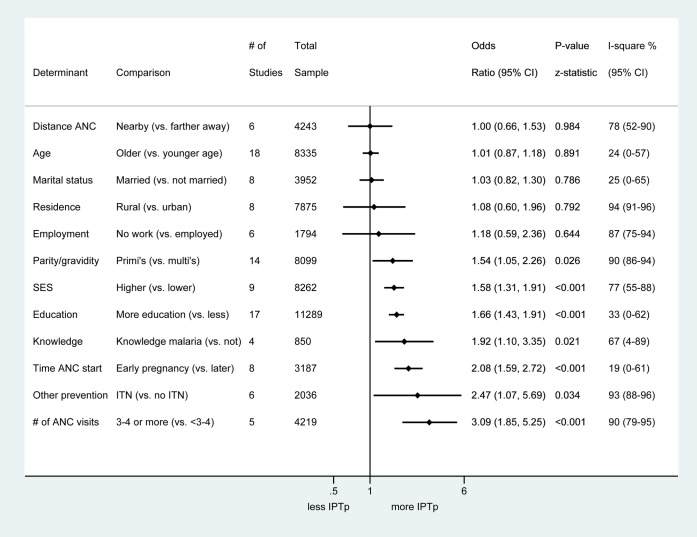
Summary odds ratios of determinants of IPTp receipt assessed in 19 studies with quantitative data. All studies used 2+ doses of SP versus less except four studies, which used 1+ doses of SP versus less; these are Mbonye 2010 [129], van Eijk 2005 [68], Nganda 2004 [125], and Napoleon 2011 [117]. SES, socio-economic status.

The key determinants of IPTp receipt among women from 31 studies were number and timing of ANC visits, parity/gravidity, education, knowledge about malaria/IPTp, socio-economic status, and use of ITNs (Figure 3; Text S2). Receipt of IPTp was higher among women making 3–4 ANC visits compared to women making fewer visits and among women first attending an ANC in their first or second trimester compared to those registering in their third trimester. Primigravidae were more likely to receive IPTp than multigravidae, with significant variation among studies (*I* ^2^ 90%, 95% CI 86–94; Text S2). More highly educated women were more likely to receive IPTp than women with less or no education, as were wealthier women. There was no association between IPTp receipt and location of residence; however, there was high heterogeneity between studies (*I* ^2^ 94%, 95% CI 91–96) and significant variation by country (*p*<0.001; Text S2). ITN users were also more likely to have received IPTp, as were women with greater knowledge of malaria. Sensitivity analysis suggested that the association between IPTp uptake and being primigravid, and between IPTp uptake and higher number of ANC visits, was stronger in the studies that were scored low-to-moderate quality than in the better quality studies (Text S2).

**Figure 4:**
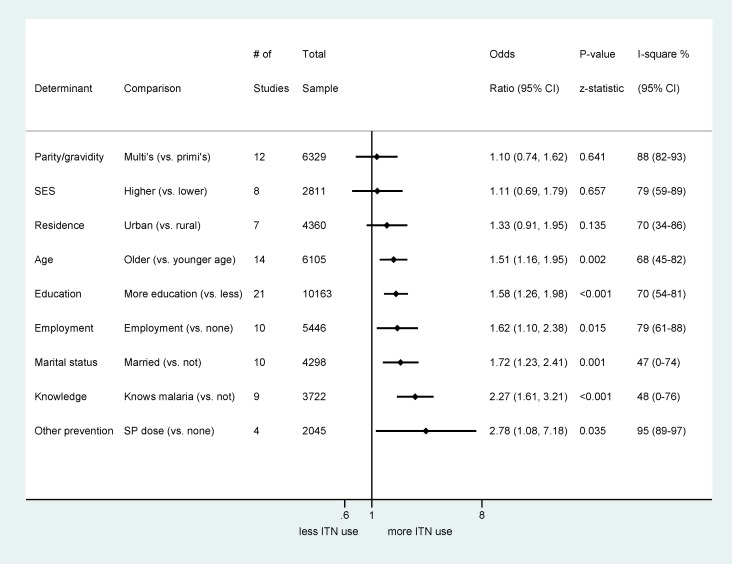
Summary odds ratios of determinants of ITN use assessed in 17 studies with quantitative data. SES, Socio-economic status.

The key determinants of ITN use among pregnant women from 27 studies were age, marital status, education, knowledge about malaria/ITNs, employment status, and receipt of IPTp (Figure 4; Text S2). Older women (aged >19 y) and married women were the most likely to use an ITN. Women with higher education or greater knowledge of malaria or ITNs were more likely to use ITNs than women with lower education or less knowledge, and women who were employed in a wage-paying job were also more likely to use ITNs during pregnancy than farmers or housewives. Women who had received IPTp were more likely to use ITNs. The effect of education on ITN use showed significant variation by country (*p* = 0.028; Text S2), and the effect of marital status on ITN use varied significantly by location of enrolment (*p* = 0.001; Text S2). Sensitivity analysis indicated a stronger association between ITN use and marital status in the low-to-moderate quality studies compared to the better quality studies (Text S2).

### Intervention Studies

#### Interventions to increase coverage of IPTp

The evidence from four studies that evaluated community-based distribution of IPTp suggests that community resources have the potential to complement the delivery of IPTp through ANCs to increase access to and uptake of IPTp among pregnant women [86]–[89] (Table 2). However, there was evidence that community-based distribution may concurrently reduce women's attendance at ANCs, though this was not consistent across the four studies: two studies showed reduced ANC attendance in the intervention sites [86],[87], and two showed increased ANC attendance [88],[89]. An alternative to delivering IPTp through community-based programmes is to employ community-based resource persons to promote IPTp, while referring women to ANCs to be given SP. This approach had substantial success in Burkina Faso, and resulted not only in higher IPTp coverage (71.8% versus 49.2% in intervention and control groups, respectively; *p*<0.001) but also in women attending the ANC earlier, in their first or second trimester (81.3% versus 70.4% in intervention and control groups, respectively; *p*<0.001), and in more women making two or more visits (89.3% versus 75.3% in intervention and control groups, respectively; *p*<0.001) [90].

One intervention study evaluated strategies to improve healthcare provider knowledge and performance on how to deliver IPTp. The study was undertaken in Kenya, 4 y after the national IPTp policy was adopted, and suggests that retraining of healthcare providers on the delivery, timing, and dosing of IPTp significantly increased coverage of IPTp (36.9% versus 10.9% in intervention and control groups, respectively; *p*<0.001) [91].

#### Interventions to increase coverage of ITNs

The included intervention studies evaluated two main channels for delivering ITNs to pregnant women: campaign delivery (non-targeted) [89],[92]–[94] and routine delivery to pregnant women through ANC services (targeted), with three alternative mechanisms evaluated at ANCs: distribution of free nets with [95]–[97] or without social marketing [98],[99], and distribution of subsidised vouchers [83],[84],[100]–[102]. One study compared the impact of ANC delivery alone versus ANC delivery plus community-based distribution of subsidised nets in Niger (Table 3).

Campaign delivery of ITNs to households with pregnant women [89], households with children under 5 y [94], or poor households [93] had limited impact on increasing coverage among pregnant women with one exception, which was a campaign in Senegal that delivered ITN vouchers to all households with children under 5 y, alongside vitamin A and mebendazole (an anthelmintic) [92] (49.2% versus 28.5% ITN coverage in intervention versus control groups, respectively; no statistical analysis reported). In a comparison study in Tanzania, the Tanzania National Voucher Scheme, which provides a voucher subsidy to pregnant women at ANCs, which is then used to purchase an ITN from a contracted retailer, achieved greater coverage than a 3-d mass campaign targeting ITNs to households with infants, based on the assumption that infants sleep with their mothers, a common practice in this setting, or ITNs sourced from retailers [94]. The voucher scheme was, however, inequitable, with fewer poorer women receiving nets [100]. In a comparison study of routine ANC delivery of ITNs alone and ANC delivery plus community-based distribution, there was no significant difference in ITN use among pregnant women between groups [103]. Routine delivery of ITNs through ANCs, by comparison, appeared to be more successful in reaching pregnant women, with four studies demonstrating an increase in ITN coverage among pregnant women compared to baseline [98],[99],[101],[102]. Programmes that delivered vouchers, as opposed to free nets, to women at ANCs experienced more operational challenges [83], and were dependent on retailers having ITN stock available [84].

Social marketing campaigns have been effective in promoting the use of ITNs in some settings through extensive media and educational campaigns that increase awareness about the benefits and importance of ITN use (especially for pregnant women), coupled with provision of readily available ITNs at low cost. They are, however, comparatively expensive to implement and sustain [104].

### Implications for Interventions to Address Barriers

We aligned the barriers to uptake of IPTp and ITNs against the findings from the intervention studies to determine the extent to which these interventions addressed known barriers (Tables 4 and 5). There were four key categories of barriers to women receiving IPTp: pregnant women's knowledge of IPTp, access to an ANC, affordability of ANC services, and quality of ANC services. Women's lack of knowledge of IPTp was very common and yet may be improved through relatively simple promotional activities delivered through all available channels, such as community-based resource persons, facility-based counselling and education, and messaging via the media and local leaders. We identified only one relevant intervention study, which evaluated community-based promotion of IPTp in Burkina Faso [90]. Women's access to an ANC was a barrier in remote settings, where community-based distribution or outreach services may be required to supplement ANC services. Four studies evaluating community-based distribution of IPTp were identified in the review, using a combination of existing [87],[88] or new community resource persons [86],[89].

**Table 4:** Synthesis matrix comparing findings from observational studies with those of intervention studies for IPTp.

Type of Factor	Findings from Observational Studies		Findings from Intervention Studies	
	Categories Derived from Barriers	Implications for Interventions to Increase Uptake	Type of Intervention Evaluated	Number of Intervention Studies
**Pregnant women factors**	**Category 1—pregnant women's knowledge**			
	Example barriers• Lack of knowledge of the preventive benefits of IPTp• Belief that use of drugs or SP in pregnancy is unsafe, e.g., could cause abortion• Fear of perceived side effects of SP• Unaware of the dangers of malaria in pregnancy	Promotion of IPTp strategy and safety of SP for IPTp through a variety of channels, e.g., community-based, clinic-based, media, local leaders	Community-based promotion of IPTp and referral of women to ANC	1 study in Burkina Faso (Gies 2009 [90])
	**Category 2—access to ANC**			
	Example barriers• Poor access to ANC• Direct and indirect costs of accessing ANC• Commitments to farming, employment, or childcare• Unwillingness to reveal pregnancy• Lack of awareness of importance of ANC services	Community-based distribution of IPTp in hard-to-reach populations with limited access to ANC, e.g., through community-based volunteers and/or community-based referral systems to increase use of ANC	Community-based distribution in settings with poor access to ANC, or community-based distribution in settings with existing drug distribution programmes, e.g., onchocerciasis, or community-based referral of women to ANC	3 studies evaluating community-based distribution of IPTp (Okeibunor 2011 [89], Msyamboza 2009 [86], Mbonye 2007 [87]); 1 study in Uganda (Ndyomugyenyi 2009 [88]); 1 study in Burkina Faso (Gies 2009 [90])
	**Category 3 –affordability of ANC services**			
	Example barriers• ANC registration fees• Laboratory fees• Cost of SP• Unofficial penalties charged by healthcare providers for late ANC attendance	See healthcare provider factors		
	**Category 4—quality of ANC services**			
	Example barriers• Providers do not offer IPTp• SP unavailable• Lack of water or cups for DOT• Poor attitudes of healthcare providers• Lack of information or instructions given by healthcare providers regarding IPTp	See healthcare provider factors		
**Healthcare provider factors**	**Category 1—provider knowledge**			
	Example barriers• Poor knowledge of IPT strategy, timing and dosage of SP• Imprecise estimation of gestational age• Confusion about when to give IPTp in relation to treatment of malaria, HIV, or other• Perception that women will or should not take SP on empty stomach	Training and supervision of healthcare providers	Training of healthcare providers	1 study in Kenya (Ouma 2007 [91])
	**Category 2—provider attitudes**			
	Example barriers• Health education not given in local language• Information and instructions on IPTp not given to pregnant women• Providers do not offer IPTp• Providers treat women with lack of respect	Training and supervision of healthcare providers on provider–client interactions	None	None
	**Category 3—health facility organisation**			
	Example barriers• Restrictive ANC hours• Lack of cups or drinking water• Frequent provider absence from work• Ineffective staff rosters	Reorganisation of staff rosters, opening hours, etc., and better management, supervision, and accountability of staff	None	None
	**Category 4—inadequate guidance on IPTp**			
	Example barriers• Variation in information given to healthcare providers on IPTp• No guidelines available at facility• Lack of supervision and monitoring of IPTp• Lack of recent training on IPTp• Private facilities following different practices• Incompatibilities between delivery of IPTp and other health interventions	Provision of consistent, simple guidelines to all health facilities, both public and private sectors, together with training and supervision	Modelling the effect of simple guidelines on coverage with IPTp	1 study in Tanzania (Gross 2011 [33])
	**Category 5—fees for ANC services**			
	Example barriers• ANC registration fees• Cost of SP• Unofficial penalties charged by healthcare providers for late ANC attendance	Modification or removal of user fees and regulation against imposition of penalties	None	None
	**Category 6—supply of SP**			
	Example barriers• SP unavailable• Poor stock control	Timely procurement and distribution systems for SP, and system to prioritise use of funds for SP at health facilities	None	None

**Table 5:** Synthesis matrix comparing findings from observational studies with those of intervention studies for ITNs.

Type of Factor	Findings from Observational Studies		Findings from Intervention Studies	
	Categories Derived from Barriers	Implications for Interventions to Increase Uptake	Type of Intervention Evaluated	Number of Intervention Studies
**Pregnant women factors**	**Category 1—pregnant women's knowledge**			
	Example barriers• Lack of knowledge of benefits of ITNs for mother and child• Discomfort of using ITNs• Lack of habit of using ITNs• Fear of chemicals used on ITNs• Perception that there are no mosquitoes	Promotion of ITN strategy and safety of insecticides used to treat nets through a variety of channels, e.g., community-based, clinic-based, media, local leaders	Promotional campaigns using a variety of channels, e.g., social marketing, clinic-based, media	3 social marketing studies by PSI in Burundi (2007 [97]), Kenya (2008 [95]), and Madagascar (2009 [96])
	**Category 2—household or cultural constraints**			
	Example barriers• Lack of support from husband and/or community• Lack of cultural habit of using ITNs• Cultural beliefs, e.g., resemblance of ITNs to burial shrouds	Promotion of ITN strategy and safety of insecticides used to treat nets through a variety of channels, e.g., community-based, clinic-based, media, local leaders	As above	As above
	**Category 3—access to ITNs**			
	Example barriers• Lack of retailers• Cost of ITNs• Inability to pay top-up fees on vouchers• Direct and indirect costs of accessing ITN distribution points	Delivery of free ITNs to pregnant women through ANC or campaigns, or delivery of voucher subsidies through ANC or campaigns, or community-based distribution of subsidised ITNs	Delivery of free ITNs to pregnant women through ANC or campaigns, or delivery of voucher subsidies through ANC or campaigns, or community-based distribution of subsidised ITNs	3 studies evaluated free ITNs: 2 studies through ANC (Pettifor 2009 [98], Guyatt 2003 [99]) and 1 study through campaign delivery (Thwing 2011 [92]); 7 studies evaluated voucher subsidies: 2 studies via campaign delivery (Ahmed 2010 [93], Khatib 2008 [94]), 5 studies via ANC (Beiersmann 2010 [84], Marchant 2010 [100], Hanson 2009 [101], Muller 2008 [102], Kweku 2007 [83]); 1 study community-based: Nonaka 2012 [103]
**Healthcare provider factors**	**Category 1—provider knowledge**			
	Example barrier• Lack of knowledge of ITN benefits for mother and child	Training and supervision of healthcare providers on ITNs	None	None
	**Category 2—provider attitudes**			
	Example barriers• Providers refuse to offer ITNs to pregnant women• Providers impose eligibility criteria for ITNs or vouchers	Better training, management, supervision, and accountability of staff	None	None
	**Category 3—health facility organisation**			
	Example barriers• Vouchers not available at facility• As for IPTp	Reorganisation of staff rosters, hours, etc., and better management, supervision, and accountability of staff	None	None
	**Category 4—fees for ANC services**			
	Example barriers• ANC registration fees• Cost of ITNs	Removal of user fees and regulation against imposition of penalties	None	None
	**Category 5—supply of ITNs/vouchers**			
	Example barriers• Poor stock control• Stockouts of ITNs• Vouchers not available	Timely procurement and distribution systems for ITNs or vouchers	None	None

Six key categories of barriers to healthcare providers delivering IPTp were identified: provider knowledge of IPTp, provider attitudes, health facility organisation, policy and guidance, fees for services, and supply of SP. Poor knowledge and poor administration of IPTp guidelines by healthcare providers appear to be substantial barriers to achieving high coverage, as highlighted in several studies included in this review. Provider knowledge of the IPTp strategy could be improved through retraining and closer supervision by district staff; however, only one study was identified that evaluated the impact of retraining of healthcare providers in Kenya on the delivery, timing, and dosing of IPTp [91]. Simplified policy and guidance on IPTp would be a relatively simple intervention to improve healthcare provider practice in delivering IPTp, and while no relevant intervention study was identified, one study in Tanzania modelled the effect of simplified guidelines on coverage with IPTp, demonstrating that coverage could be increased with simplified guidance [33]. No intervention studies were identified that addressed supply of SP, even though this was one of the commonest barriers identified in the observational studies. Poor healthcare provider attitude is a generic problem often entrenched in resource-constrained healthcare system and public sector settings, and may be difficult to address; no relevant intervention studies were identified. Similarly, user fees at ANCs are a generic barrier to ANC services, and no intervention studies were identified that addressed this.

Three key categories of barriers to women receiving and using ITNs were identified: pregnant women's knowledge of ITNs, household or cultural constraints, and access to ITNs. As for IPTp, pregnant women's knowledge of ITNs as well as certain household and cultural constraints could be addressed through promotion of ITNs through a variety of channels. Social marketing using extensive media and educational campaigns has been used in a large number of countries, and three evaluation studies were identified in this review [95]–[97]. Access to ITNs has been a problem for women in terms of direct and indirect costs, ITN availability, and access to distribution points. Three studies evaluated the delivery of free ITNs to pregnant women through ANCs [98],[99] or campaigns [92], one study evaluated community-based delivery of subsidised ITNs [103], and seven studies evaluated voucher subsidies delivered through ANCs [83],[84],[100]–[102] or campaigns [93],[94]. Categories of barriers to healthcare providers delivering ITNs were similar to those for the delivery of IPTp: provider knowledge, provider attitudes, health facility organisation, fees for services, and supply of ITNs. We did not find any relevant studies that evaluated interventions that directly addressed these provider barriers.

## Discussion

To our knowledge this is the first systematic review of the factors affecting the delivery, access, and use of interventions to prevent malaria in pregnant women that uses research findings from quantitative, qualitative, and mixed methods studies, that assesses both user and provider perspectives, and that integrates these findings with intervention studies. This analysis provides a comprehensive basis for identifying key bottlenecks in the delivery and uptake of IPTp and ITNs among pregnant women, and for understanding which scale-up interventions have been effective, in order to prioritise which interventions are most likely to have the greatest impact in the short or medium term.

Barriers to the delivery of IPTp and ITNs were found at different levels of implementation, and broadly fall into policy and guidance, healthcare system issues, health facility issues, and healthcare provider performance. Whilst many of the barriers reflected broader weaknesses in the healthcare system, some were specific to the intervention. With regard to IPTp, a key identified barrier to effective delivery was healthcare provider confusion about the timing of the two doses of IPTp and whether IPTp can be given on an empty stomach. This confusion stemmed from a combination of unclear policy and guidance, inadequate training, and lack of information and job aids on IPTp. Several studies reported conflicting national policies with regards to provision of IPTp in relation to management of HIV and other diseases or conditions, and when to give IPTp if women have been treated for malaria, a problem also identified in another review [105]. Also, some studies reported that healthcare providers expressed uncertainty over the effectiveness of SP for IPTp. Clearly there is an urgent need for countries to update national IPTp policy and guidance, and to ensure that this information reaches frontline providers at ANCs and outpatient departments providing treatment to pregnant women for illness, e.g., through directives or memos from the Director of Medical Services, as done in Kenya (M. J. Hamel, personal communication). The recent WHO IPTp policy update recommendation with simplified guidance on IPTp dosing, which also restates the continued effectiveness of IPTp with SP, serves as an important opportunity for national programmes to update and reinvigorate their IPTp strategy [106].

Organisational problems at the facility level were also common, such as lack of privacy and confidentiality in the health encounter [51] and the restriction of hours of ANC services, resulting in high client-to-staff ratios, long waiting times [49],[52], and reduced consultation times, all of which contribute to poor quality of care at ANCs. Absenteeism and high staff rotation at the facility leading to lack of continuity of care and high workload among staff on duty was also reported [62]. Most of these organisational problems present another area for improvement in the short term that does not require additional resources, though it will require better management and accountability by the heads of health facilities. Other barriers were, however, dependent on higher levels of the healthcare system, such as high staff turnover [62], understaffing (particularly in remote areas), poor infrastructure [41], poor supervision, and poor use of data to identify problems and inform decision-making. These problems are inherent in the healthcare systems in some areas in some countries, and will require longer term strategies and increased investment in healthcare system strengthening. Also persistently reported across the studies and dependent on action taken at higher levels were stockouts of both SP for IPTp and ITNs, and lack of water or cups for providing IPTp by DOT. The reviewed studies did not explore the reasons for the stockouts, but they are likely to be a combination of lack of funding at the national level for procurement of commodities (i.e., specific to IPTp and ITNs) and problems in supply chain management.

Barrier studies among women highlighted additional healthcare system barriers leading to poor uptake of IPTp and/or ITNs. Having to pay user fees or pay for SP, drinking water for DOT, or ITNs was a common barrier, as were the indirect costs associated with visiting ANCs, such as transport, food, and opportunity costs. This finding was supported by the meta-analysis of determinants of coverage among pregnant women, which showed that socio-economic status and employment status are important predictors of IPTp and ITN coverage, respectively. These inequities may to some extent reflect the determinants of women's access to ANCs, where user fees are routinely applied to registration, consultations, laboratory tests, and drugs, as identified in a review of factors affecting utilisation of antenatal care in developing countries [107]. However, in some instances user fees are also applied to SP (e.g., where women have to purchase SP or water to take IPTp by DOT) and to ITNs [108]. This situation calls for a review of charging policies for IPTp and ITNs across national programmes, and of user fees and charges at ANCs in general. Another common barrier to ANC utilisation was the poor quality of interactions between healthcare providers and pregnant women [38],[41]. Women were generally perceived as passive recipients and were provided with little or no information about the services provided [44], and women with a low social position, such as adolescents [51], and less educated women are most vulnerable. This issue appears to be a problem in some resource-poor settings and is more difficult to tackle. However, educating women about their rights and about the ANC services available to them may go some way to empowering women to be able to demand better services.

This finding is supported by the fact that pregnant women's lack of knowledge and understanding of IPTp and ITNs was consistently reported in both the barrier and determinant data as an important factor preventing the uptake and use of IPTp and ITNs. Women who understand the benefits of IPTp and the safety of SP, and how and when to take it, are more likely to take it. However, many women do not receive adequate information about IPTp, and this can result in fears that the drug causes harm, even abortion [15], or women showing preference for an alternative drug. Whilst there are some reports that women experience side effects from IPTp, the severity and extent of these events are not clearly described. There were also reports of women fearing that the chemical used on ITNs would harm the foetus [15]. Whilst knowledge is also an important facilitator of ITN use, barrier studies reveal important deterrents to ITN use such as the inconvenience and discomfort of use [109], especially in the dry season, and the lack of a culture or habit of net use. These findings were consistent with the meta-analysis of determinants in that coverage of both IPTp and ITNs was lower among women with no education and, in some countries, women living in rural areas; these women were less likely to access ANC and/or health education services. The meta-analysis was useful in identifying other important risk groups. Younger or adolescent women, unmarried women, and less educated women were significantly less likely to use ITNs. The barrier studies show that this may be related to lower affordability and in-household access among these women. Adolescents, unmarried women, and less educated women therefore constitute high-risk groups for targeting ITNs. This suggests that ministries of health need to pay more attention to IPTp and ITN promotion and health education, with additional targeting of risk groups, as well as using new innovations for communication of messages, since traditional health education is not offered at all facilities or is not always effective.

Women seeking care at ANCs often have to overcome barriers at the household or societal level, and these barriers are more challenging to address. Women have commitments to farming or employers and the responsibility of childcare, and often have to defer to their husbands or in-laws in decision-making over accessing ITNs or use of household income to pay for ANC services. In a review of ANC access, use of ANCs was shown to increase with husband's educational level and was an even stronger predictor than women's education in some settings [107]. Local cultural norms and practices present a considerable barrier to women accessing ANC services in some but not all study countries, with wide variation within countries and between countries, a finding also reported in the review by Pell et al. [15].

In comparison to the observational studies, the review identified comparatively few studies that evaluated interventions to promote scale-up of these interventions, particularly for IPTp. Whilst many of the barriers to IPTp and ITN coverage identified in the observational studies related to healthcare providers and service delivery, very few studies that evaluated interventions to improve service delivery were found. Similarly, very few studies explored the determinants of delivery of either IPTp or ITNs among healthcare providers, or supply-side interventions designed to improve the quality of delivery of IPTp or of ITNs with a chosen strategy, whether it be campaigns or routine delivery through ANCs. Of the six studies that evaluated interventions to increase coverage of IPTp, all but one targeted women's knowledge or access, the last being a healthcare provider intervention. Consideration of the context for employing community-based distribution of IPTp is important; this distribution strategy appears to be an effective additional strategy to boost coverage in areas where there is already a successful community-based distribution programme, as seen in the onchocerciasis control programme in Uganda [88], but may serve to undermine women's attendance at ANCs in areas where ANC attendance is fragile. Community-based promotion, on the other hand, has the potential benefit in some settings of increasing access and uptake of IPTp by providing women with information about the importance and benefits of IPTp, and at the same time reinforcing the message that women should obtain antenatal care from ANCs, where they benefit from the full range of focussed ANC services [90]. While 13 studies were identified that evaluated the effectiveness of alternative delivery strategies to increase ITN coverage among pregnant women, the study objectives and designs were heterogeneous; hence, it was not possible to draw generalisable conclusions. Nevertheless, ANC services appear to be an important source of free ITNs for pregnant women in rural areas, a finding supported in a review of best practices of ITN programmes in sub-Saharan eastern Africa [108].

### Strengths and Limitations of the Review

The review triangulates data from quantitative, qualitative, and mixed methods studies to increase the content validity and comprehensiveness of the review; it does not, however, attempt a full meta-ethnography of qualitative data, which has been undertaken recently by others [15],[110]. The meta-analysis of determinants was used to explore the range of effects between studies and to provide a pooled analysis to support the findings of the narrative (interpretive) synthesis. Although the use of cluster-unadjusted ORs may have overestimated precision, these were limited to four out of 36 studies. There was considerable heterogeneity among studies included in the meta-analysis, and we explored only a limited number of variables in the subgroup analysis to assess whether these could explain the differences between studies (Text S2). The lack of adjustment for ANC attendance in studies using community-based surveys means that the determinants of IPTp use may be partly driven by determinants of ANC access. However, the differences in the results between studies that enrolled women in the community and those that enrolled women in clinics in the subgroup analysis were not significant (Text S2). Whilst distinguishing between use of SP for treatment versus use for prevention poses an important challenge in interpreting community surveys, this limitation was not measured in the studies included in the meta-analysis. Whilst no restrictions were placed on the language of publication, and no studies were excluded on the basis of language, the focus the Malaria in Pregnancy Library (the primary source of studies) to date has been on the European family of languages and predominantly English. Reviewer bias was limited by the use of two independent reviewers to assess inclusion criteria. Reporting of included studies was assessed for quality, and reporting quality for the majority of studies was assessed to be fair. There were three quantitative studies that met no reporting quality criteria and 13 studies that met only one criterion (10 quantitative and three intervention studies). Findings from the studies with data on barriers were found to be entirely consistent with findings from other studies, and provided no new or surprising themes, and inclusion of these studies did not alter the study findings.

Our review includes 98 studies from across sub-Saharan Africa, with 77 of these specifically containing data on barriers and determinants of delivery, access, and use of IPTp and ITNs among healthcare providers and pregnant women; this is a sizeable body of evidence. In summary, the delivery and uptake of IPTp and ITNs by pregnant women is impeded by a wide range of factors among both pregnant women and the healthcare system, each influenced by an array of social, cultural, economic, and institutional factors, with each factor influenced by the others in a complex interchange. There are also geographic variations, with some barriers more prominent in some countries than in others. Notwithstanding this complexity, many of the barriers highlighted in this review are relatively consistent across countries and are surmountable: barriers that programmes can address in the near term with limited additional investment. Delivery of ITNs through ANCs presents a narrower range of problems than delivery of IPTp. Actions to increase coverage of IPTp and ITNs in the short term would be (1) to simplify country policies and guidance to align the updated WHO IPTp policy [106] with the new WHO policy for focused antenatal care, consisting of four visits in the second and third trimesters, and ensure effective dissemination to frontline healthcare providers through training and job aids; (2) to earmark funding for procurement of SP and ITNs; (3) to review ANC fee structures; and (4) to launch targeted promotional campaigns to reach high-risk populations of pregnant women, according to local settings, e.g., rural, poor, or adolescent women. Promotional campaigns will need to reflect the needs of women and offer services they will accept at a price they can afford.

Other barriers are more entrenched within the overall healthcare system and will require medium- to long-term strategies to improve the overall quality of antenatal services and encourage the habit of ANC use among women. New multifaceted interventions should be explored, such as quality improvement initiatives that link improvements in delivery of IPTp and ITNs to other core ANC services, management tools for facility-level decision-making, and innovations, such as use of mobile phones for defaulter tracing, supply chain/stock control, reporting of health management information systems data on coverage, and surveillance. Increasing drug resistance means that IPTp with SP will most likely be replaced by more complicated and expensive drug regimens [4],[111], or new strategies, such as intermittent screening and treatment [112]. Intermittent screening and treatment will require adjustments to be made in the ANC setting [47],[64], and will not have the added benefit of IPTp in controlling infections that cannot be detected by rapid diagnostic tests or microscopy. Malaria prevention estimates have increased only modestly between 2007 and 2010 (from 13.6% to 21.5% coverage for IPTp and from 17.0% to 38.8% coverage for ITN use) [138].

## Conclusion

Our synthesis shows that the key barriers to access, delivery, and use of IPTp and ITNs are relatively consistent across countries. These barriers may be helpful as a checklist for use by country malaria programmes and/or policy-makers to identify factors influencing uptake of these interventions in their specific location or context. The review also highlights the need for multi-country studies that evaluate targeted or multifaceted interventions aimed to improve the delivery and uptake of IPTp and ITNs. More research is also needed to understand and improve the policy change process to facilitate future replacement of SP with alternative drug regimens for IPTp or alternative strategies such as screening and treatment that will present even greater challenges for delivery.

## Supporting Information

Table S1 **Search terms and databases used in the review.**
(DOCX)

Table S2 **Study characteristics.** Table S2.1. Characteristics of studies on determinants, barriers, and facilitators. Table S2.2. Characteristics of intervention studies.
(DOCX)

Table S3 **Checklist for quality of reporting.** Table S3.1. Checklist for quality of reporting: quantitative studies. Table S3.2. Checklist for quality of reporting: qualitative studies. Table S3.3. Checklist for quality of reporting: mixed methods studies. Table S3.4. Checklist for quality of reporting: intervention studies.
(DOCX)

Table S4 **Barriers and facilitators to delivery, access, and use of IPTp and ITNs.** Table S4.1. Barriers and facilitators to receipt of IPTp from the perspective of pregnant and recently delivered women. Table S4.2. Barriers and facilitators to ITN ownership and use from the perspective of pregnant and recently delivered women. Table S4.3. Barriers and facilitators to the delivery of IPTp from the healthcare provider perspective. Table S4.4. Barriers and facilitators to the delivery and use of ITNs from the healthcare provider perspective.
(DOCX)

Text S1 **PRISMA statement.**
(DOC)

Text S2 **Meta-analysis of determinants of IPTp and ITN use in pregnancy.**
(PDF)

## Abbreviations

ANC: antenatal clinic
DOT: directly observed therapy
IPTp: intermittent preventive treatment in pregnancy
ITN: insecticide-treated net
OR: odds ratio
SP: sulphadoxine-pyrimethamine
WHO: World Health Organization
