# Supplementary material for: Factors Affecting the Delivery, Access, and Use of Interventions to Prevent Malaria in Pregnancy in Sub-Saharan Africa: A Systematic Review and Meta-Analysis
Source: PLoS Med. 2013 Jul 23;10(7):e1001488. doi: 10.1371/journal.pmed.1001488 (PMC3720261; doi:10.1371/journal.pmed.1001488)
Supplement: Table S1 — Search terms and databases used in the review. (DOCX) [file pmed.1001488.s001.docx]

**Table S1. Search terms and databases used in the review.**

| **Global Health Search** | **MiP Library Search†** |
| --- | --- |
| Malaria  **AND** | ---- |
| pregnant wom* OR provider* OR ANC service* OR antenatal service* OR ANC OR health provider OR health work* OR health servic* provider OR gestation*  **AND** | ---- |
| intermittent preventive treatment* OR intermittent presumptive therap* OR intermittent protective treatment* OR ipt OR iptp OR LLIN OR long lasting insecticid* treated net* OR insecticid* treated net* OR itn OR bed net* OR bed-net* OR bednet* OR mosquito net* OR mosquito-net* OR sulphadoxine pyrimethamine OR sulfadoxine pyrimethamine OR sp OR chemoprophyla* OR chemoprevent*  **AND** | intermittent preventive treatment* OR intermittent presumptive therap* OR intermittent protective treatment* OR ipt OR iptp OR LLIN OR long lasting insecticid* treated net* OR insecticid* treated net* OR itn OR bed net* OR bed-net* OR bednet* OR mosquito net* OR mosquito-net* OR sulphadoxine pyrimethamine OR sulfadoxine pyrimethamine OR sp OR chemoprophyla* OR chemoprevent*  **AND** |
| utilisation OR utilization OR coverage OR barrier* OR attendance OR compliance OR adherence OR attitude* OR knowledge OR practic* OR belie* OR delivery effectiv* OR determinant* OR distribut* OR evaluat* OR delivery system* OR predictor OR DOT* OR directly observed OR uptake OR behaviour* OR behavior* OR perception* OR acceptance OR availability OR awareness | utilisation OR utilization OR coverage OR barrier* OR attendance OR compliance OR adherence OR attitude* OR knowledge OR practic* OR belie* OR delivery effectiv* OR determinant* OR distribut* OR evaluat* OR delivery system* OR predictor* OR gestation* OR DOT* OR directly observed OR uptake OR behaviour* OR behavior* OR perception* OR acceptance OR availability OR awareness |

†All material in the Malaria in Pregnancy library contain the words *malaria* and *pregnant woman*
