# Supplementary material for: Factors Affecting the Delivery, Access, and Use of Interventions to Prevent Malaria in Pregnancy in Sub-Saharan Africa: A Systematic Review and Meta-Analysis
Source: PLoS Med. 2013 Jul 23;10(7):e1001488. doi: 10.1371/journal.pmed.1001488 (PMC3720261; doi:10.1371/journal.pmed.1001488)
Supplement: Table S2 — Study characteristics. Table S2.1. Characteristics of studies on determinants, barriers, and facilitators. Table S2.2. Characteristics of intervention studies. (DOCX) [file pmed.1001488.s002.docx]

Tables S2: Study characteristics.

Table S2.1. Characteristics of studies on determinants, barriers and facilitators.

| **Author/**  **Year** | **Region** | **Country** | **Scale** | **Urban/ Rural** | **Transmission setting** | **Study period/**  **Year** | **Target group (type and number)** | **Sample** | **N** | **Data Type** | **Study design** | **IPTp/ ITN** | **Primary outcome(s)** |
| --- | --- | --- | --- | --- | --- | --- | --- | --- | --- | --- | --- | --- | --- |
| **IPTp** | | | | | | | | | | | | | |
|  |  |  |  |  |  |  |  |  |  |  |  |  |  |
| Amoran, 2012a [[1](#_ENREF_1)] | West Africa | Nigeria | 1 district | Rural | Perennial | 2008 | RD | ANC based | 255 | Quant | Cross-sectional | IPTp | IPTp use |
| Arulogun, 2012 [[2](#_ENREF_2)] | West Africa | Nigeria | 1 district | Urban | Perennial | NR | PW/HW | ANC based | 408 | Mixed | Cross-sectional | IPTp | IPTp use |
| Diala, 2012 [[3](#_ENREF_3)] | West Africa | Nigeria | >1 district | Urban/rural | Perennial | 2009 | PW | ANC based | 30 | Qual | Cross-sectional | IPTp | IPTp use |
| Iliyasu, 2012 [[4](#_ENREF_4)] | West Africa | Nigeria | 1 district | Urban | Perennial | 2011 | PW | ANC based | 239 | Quant | Cross-sectional | IPTp | IPTp use |
| Mubyazi, 2012 [[5](#_ENREF_5)] | East Africa | Tanzania | >1 district | Rural | Perennial | 2005-2006 | HW | ANC based | 78 | Mixed | Cross-sectional | IPTp | IPTp acceptability |
| Mutagonda, 2012 [[6](#_ENREF_6)] | East Africa | Tanzania | 1 district | Urban/rural | Perennial | 2010-2011 | PW | ANC based | 470 | Mixed | Cross-sectional | IPTp | IPTp use |
| Namusoke, 2012 [[7](#_ENREF_7)] | East Africa | Uganda | 1 district | Urban | Perennial | 2008-2009 | PW | Hospital based | 204 | Quant | Cross-sectional | IPTp | IPTp use |
| Onoka, 2012a [[8](#_ENREF_8)] | West Africa | Nigeria | >1 district | Urban/rural | Perennial | 2010 | PW/RD | Hospital based | 1307 | Mixed | Cross-sectional | IPTp | IPTp use |
| Onoka, 2012b [[9](#_ENREF_9)] | West Africa | Nigeria | >1 district | Urban/rural | Perennial | 2010 | HP | ANC based | 34 | Mixed | Cross-sectional | IPTp | IPTp guidelines |
| Onwujekwe, 2012 [[10](#_ENREF_10)] | West Africa | Nigeria | 1 district | Urban | Perennial | 2010 | HW | Hospital based | 52 | Quant | Cross-sectional | IPTp | IPTp knowledge/use |
| Ansah-Ofei, 2011 [[11](#_ENREF_11)] | West Africa | Ghana | 1 district | Rural | Perennial | NR | PW/RD | Population based | 100 | Mixed | Cross-sectional | IPTp | IPTp use |
| D’Almeida, 2011[[12](#_ENREF_12)] | West Africa | Benin | >1 district | Urban/ rural | Perennial | 2005-2009 | PW | Hospital based | 2420 | Quant | Longitudinal | IPTp | IPTp use |
| Donkor, 2011 [[13](#_ENREF_13)] | West Africa | Ghana | 1 district | Urban/ rural | Perennial | 2009 | PW/HW | ANC based | 88 | Quant | Cross-sectional | IPTp | IPTp knowledge |
| Faye, 2011 [[14](#_ENREF_14)] | West Africa | Senegal | National | Urban/ rural | NR | 2006 | 15-49 yrs | Population based | 1906 | Quant | Cross-sectional | IPTp | IPTp 2 |
| Nduka, 2011 [[15](#_ENREF_15)] | West Africa | Nigeria | >1 district | Urban/rural | Perennial | NR | PW | Hospital based | 844 | Quant | Cross-sectional | IPTp | IPTp use |
| Tutu, 2011 [[16](#_ENREF_16)] | West Africa | Ghana | 1 district | Rural | Perennial | 2005-2007 | RD/HW | Hospital based | 2583/ 37 | Mixed | Survey/interview | IPTp | IPTp knowledge |
| Smith Paintain, 2011 [[17](#_ENREF_17)] | West Africa | Ghana | >1 district | NR | Perennial | 2009 | HW | ANC based | 134 | Mixed | Survey/interview | IPTp | IPTp knowledge |
| Gross, 2011 [[18](#_ENREF_18)] | East Africa | Tanzania | >1 district | Rural | Perennial | 2007-2008 | PW/HW | ANC based | 440/18 | Mixed | Survey/interview | IPTp | IPTp use/IPTp delivery |
| Ndyomugyenyi, 2010 [[19](#_ENREF_19)] | East Africa | Uganda | 1 district | NR | Perennial | 2007-2008 | RD1 | Community based | 453 | Quant | Cross-sectional | IPTp | ANC visits/IPTp 2 |
| Sangare, 2010a [[20](#_ENREF_20)] | East Africa | Uganda | 1 district | Urban | Seasonal | 2008-2009 | RD12 | Community based | 500 | Quant | Cross-sectional | IPTp | IPTp 2 |
| Mbonye, 2010 [[21](#_ENREF_21)] | East Africa | Uganda | 1 district | Rural | Perennial | 2009 | 15-49yrs | Community based | 9758 | Mixed | Survey/interview | IPTp | IPTp use |
| Sande, 2010 [[22](#_ENREF_22)] | East Africa | Kenya | 1 district | Urban/ rural | Perennial | 2007 | PW/RD/HW | Hospital based | 325 | Mixed | Survey/interview/FGD | IPTp | ANC visits/IPTp use |
| Grietens, 2010 [[23](#_ENREF_23)] | West Africa | Burkina Faso | 1 district | Rural | Perennial/SP | 2003-2006 | PW | Community based | 2240 | Mixed | Survey/interview/FGD | IPTp | ANC attendance/IPTp 2 |
| Antwi, 2010 [[24](#_ENREF_24)] | West Africa | Ghana | 1 district | Rural | Perennial/SP | 2009 | PW/RD3/HW | ANC based | 168/ 420/ 30 | Mixed | Survey/interview | IPTp | ANC access/IPTp 2 |
| Smith, 2010 [[25](#_ENREF_25)] | West Africa | Ghana | >1 district | Rural | Perennial/SP | 2009 | PW | ANC based | NR | Qual | FGD | IPTp | IPTp acceptance |
| Akinleye, 2009 [[26](#_ENREF_26)] | West Africa | Nigeria | >1 district | Rural | Perennial | 2007 | PW | ANC based | 209 | Quant | Cross-sectional | IPTp | IPTp 1 |
| Takem, 2009 [[27](#_ENREF_27)] | West Africa | Cameroon | 1 district | NR | Perennial | 2006-2007 | PW | ANC based | 527 | Quant | Cross-sectional | IPTp | IPTp 2 |
| Acquah, 2009 [[28](#_ENREF_28)] | West Africa | Ghana | 1 district | Rural | NR | 2008 | PW/RD3 | Community based | 641 | Quant | Cross-sectional | IPTp | IPTp 3 |
| Klebi, 2009 [[29](#_ENREF_29)] | West Africa | Ghana | 1 district | Rural | Perennial | NR | PW/RD7/HW | ANC based | 900 | Quant | Cross-sectional | IPTp | IPTp use |
| Gies, 2009 [[30](#_ENREF_30)] | West Africa | Burkina Faso | 1 district | Rural | Perennial | 2003-2006 | PW | Community based | 2766 | Quant | Cross-sectional | IPTp | ANC visits/IPTp 2 |
| Adjei, 2009 [[31](#_ENREF_31)] | West Africa | Ghana | 1 district | NR | NR | NR | RD3/HW | Hospital based | 320 | Mixed | Survey/interview | IPTp | ANC access/IPTp 1/2 |
| Brabin, 2009 [[32](#_ENREF_32)] | West Africa | Gambia | 1 district | Rural | NR | 2007 | PW | Community based | NR | Qual | FGD/interview | IPTp | IPTp knowledge |
| Marchant, 2008 [[33](#_ENREF_33)] | East Africa | Tanzania | National | Urban/ rural | Perennial | 2005-2007 | RD12 | Population based | 1171/1229/ 1214 | Quant | Cross-sectional | IPTp | IPTp 1/2 |
| Anders, 2008 [[34](#_ENREF_34)] | East Africa | Tanzania | >1 districts | Urban/ rural | Perennial/SP | 2006 | PW/HW | ANC based | 113/12 | Mixed | Interview/ observation | IPTp | ANC timing/IPTp 2 |
| Mubyazi, 2008 [[35](#_ENREF_35)] | East Africa | Tanzania | National | NR | NR | 2006-2007 | HW | Hospital based | 11 | Qual | Interviews | IPTp | IPTp use/IPTp delivery |
| Ouma, 2007 [[36](#_ENREF_36)] | East Africa | Kenya | 1 district | Rural | Perennial | 2005 | RD1-4 | Community based | 726 | Quant | Cross-sectional | IPTp | IPTp 1/2 |
| Launiala, 2007 [[37](#_ENREF_37)] | East Africa | Malawi | 1 district | Rural | NR | 2002 | PW/HW | ANC based | 282 | Mixed | Survey/interview/ FGD/observation | IPTp | ANC access/IPTp delivery |
| Mbonye, 2006a [[38](#_ENREF_38)] | East Africa | Uganda | 1 district | Rural | Perennial | 2002-2003 | PW | Community based | 90 | Qual | Interview/FGD | IPTp | ANC access/IPTp use |
| Gates Malaria Partnership, 2005 [[39](#_ENREF_39)] | West Africa | Gambia | >1 district | NR | NR | 2002-2004 | PW/HW | ANC based | 111 | Mixed | Interview/FGD | IPTp | ANC access/IPTp use |
| Mubyazi, 2005 [[40](#_ENREF_40)] | East Africa | Tanzania | 1 district | Rural | NR | 2004 | PW/HW | Hospital based | 89 | Qual | Interview/FGD | IPTp | ANC access/IPTp use |
| Holtz, 2004 [[41](#_ENREF_41)] | East Africa | Malawi | 1 district | Urban | NR | 2000 | RD24 | Community based | 391 | Quant | Cross-sectional | IPTp | IPTp 2 |
| Ashwood-Smith, 2002 [[42](#_ENREF_42)] | East Africa | Malawi | 1 district | Urban | NR | 2001 | PW/HW | ANC based | 287/41 | Mixed | Survey/interview | IPTp | IPTp 1 /2 timing |
| **ITN** | | | | | | | | | | | | | |
| Aluko, 2012 [[43](#_ENREF_43)] | West Africa | Nigeria | 1 district | Urban/rural | Perennial | 2008 | RD | ANC based | 335 | Quant | Cross-sectional | ITN | ITN use/ownership |
| Amoran, 2012b [[44](#_ENREF_44)] | West Africa | Nigeria | 1 district | Urban/rural | Perennial | 2007 | PW | ANC based | 300 | Quant | Cross-sectional | ITN | ITN use/ownership |
| Ankomah, 2012 [[45](#_ENREF_45)] | West Africa | Nigeria | >1 district | Urban/rural | Perennial | 2008 | PW | Population based | 2348 | Quant | Cross-sectional | ITN | ITN use/ownership |
| Auta, 2012 [[46](#_ENREF_46)] | West Africa | Nigeria | National | Urban/rural | Perennial | 2008 | 15-49 yrs | Population based | 34070 | Quant | Cross-sectional | ITN | ITN use/ownership |
| Ambrose, 2011 [[47](#_ENREF_47)] | East Africa | Tanzania | 1 district | NR | NR | 2009 | PW | ANC based | 222 | Quant | Cross-sectional | ITN | ITN use/ownership |
| Okonta, 2011 [[48](#_ENREF_48)] | West Africa | Nigeria | 1 district | Urban/Rural | Perennial | 2009 | RD | Hospital based | 537 | Quant | Cross-sectional | ITN | ITN use |
| Olajide, 2011 [[49](#_ENREF_49)] | West Africa | Nigeria | 1 district | Urban | Perennial | 2010 | PW | ANC based | 320 | Quant | Cross-sectional | ITN | ITN use |
| O’Meara, 2011 [[50](#_ENREF_50)] | East Africa | Kenya | 1 district | Rural | Perennial | 2009-2010 | PW | Community based | 2988 | Quant | Cross-sectional | ITN | ITN ownership |
| Karunamoorthi, 2010 [[51](#_ENREF_51)] | East Africa | Ethiopia | 1 district | Urban | Seasonal | 2008 | PW | ANC based | 225 | Quant | Cross-sectional | ITN | ITN use |
| Wagbatsoma, 2010 [[52](#_ENREF_52)] | West Africa | Nigeria | >1 district | Rural | Perennial | NR | PW | ANC based | 385 | Quant | Cross-sectional | ITN | ITN use |
| Sangare, 2010b [[53](#_ENREF_53)] | East Africa | Uganda | 1 district | Urban | Seasonal | 2008-2009 | RD12 | Community based | 500 | Quant | Cross-sectional | ITN | ITN use/ownership |
| Beiersmann, 2010 [[54](#_ENREF_54)] | West Africa | Burkina Faso | 1 district | Urban/ rural | Perennial/SP | 2006 | HW | Community based | 4/11 | Qual | Interview/FGD | ITN | ITN delivery |
| Musa, 2009 [[55](#_ENREF_55)] | West Africa | Nigeria | >1 district | Urban/ rural | NR | 2006 | PW | ANC based | 455 | Quant | Cross-sectional | ITN | ITN use |
| Njoroge, 2009 [[56](#_ENREF_56)] | East Africa | Kenya | 1 district | NR | Perennial | 2007 | PW | ANC based | 220 | Mixed | Survey/interview/ FGD | ITN | ITN use |
| Pettifor, 2008 [[57](#_ENREF_57)] | Central Africa | DRC | 1 district | Urban | Perennial/SP | 2005 | PW | ANC based | 351 | Quant | Cross-sectional | ITN | ITN use/ownership |
| Belay, 2008 [[58](#_ENREF_58)] | East Africa | Ethiopia | 1 district | Urban/ rural | Seasonal | 2006 | PW | Community based | 815 | Mixed | Survey/FGD | ITN | ITN use |
| Hassan, 2008 [[59](#_ENREF_59)] | East Africa | Sudan | 1 district | NR | NR | NR | PW | Community based | 19 | Mixed | Survey/FGD | ITN | ITN use |
| Mnyika, 2007 [[60](#_ENREF_60)] | East Africa | Tanzania | 1 district | Urban | NR | NR | PW | ANC based | 729 | Quant | Cross-sectional | ITN | ITN use |
| Kweku, 2007 [[61](#_ENREF_61)] | West Africa | Ghana | >1 district | Urban/ rural | NR | 2004-2005 | PW/HW | ANC based | 957 | Mixed | Facility data/Interview | ITN | ITN delivery |
| PSI, 2006 Z [[62](#_ENREF_62)] | Southern Africa | Zambia | National | Urban/ rural | NR | 2005 | PW | Population based | 268 | Quant | Cross-sectional | ITN | ITN use |
| PSI, 2006 B [[63](#_ENREF_63)] | Central Africa | Burundi | National | Urban/ rural | NR | 2005 | PW | Population based | 721 | Quant | Cross-sectional | ITN | ITN use |
| PSI, 2006 R [[64](#_ENREF_64)] | Central Africa | Rwanda | National | Urban/ rural | NR | 2005 | PW | Population based | NR | Quant | Cross-sectional | ITN | ITN use |
| Van Geertruyden, 2005 [[65](#_ENREF_65)] | Central Africa | Rwanda | >1 district | NR | Perennial/SP | 2002 | PW | ANC based | 1432 | Quant | Cross-sectional | ITN | ITN use |
| Mbonye, 2006b [[66](#_ENREF_66)] | East Africa | Uganda | 1 district | Rural | Perennial | 2002-2003 | PW | Community based | 90 | Qual | Interview/FGD | ITN | ITN use |
| Marchant, 2002 [[67](#_ENREF_67)] | East Africa | Tanzania | 1 district | Rural | Perennial | 1998-1999 | PW | Community based | 671 | Quant | Cross-sectional | ITN | ITN use |
| **IPTp & ITN** | | | | | | | | | | | | | |
| Akaba, 2013 [[68](#_ENREF_68)] | West Africa | Nigeria | 1 district | Urban/rural | Perennial | 2010 | PW | ANC based | 403 | Quant | Cross-sectional | IPTp/ ITN | IPTp use/ ITN use |
| De Allegri, 2013 [[69](#_ENREF_69)] | West Africa | Burkina Faso | >1 district | Rural | Perennial | 2006-2008 | PW | Population based | 1106 | Quant | Cross-sectional | IPTp/ITN | ITN/IPTp coverage |
| Hill, 2013 [[70](#_ENREF_70)] | East Africa | Kenya | 1 district | Rural | Perennial | 2010 | PW/RD | Population based | 339 | Quant | Cross-sectional | IPTP/ ITN | IPTp use/ ITN use |
| Bouyou-Akotet, 2012 [[71](#_ENREF_71)] | West Africa | Gabon | >1 district | Urban | Perennial | 2011 | 15-49 yrs | ANC based | 1030 | Quant | Cross-sectional | IPTp/ITN | IPTp use/ITN use |
| Zere, 2012 [[72](#_ENREF_72)] | West Africa | Ghana | >1 district | Urban/rural | Perennial | 2008 | 15-49 yrs | Population based | 4916 | Quant | Cross-sectional | IPTp/ITN | DHS Survey Coverage |
| Manirakiza, 2011 [[73](#_ENREF_73)] | West Africa | Central African Republic | 1 district | Urban | Perennial | 2009 | PW | Hospital based | 328 | Quant | Cross-sectional | IPTp/ITN | IPTp acceptability/ITN usage |
| Napoleon, 2011 [[74](#_ENREF_74)] | East Africa | Sudan | 1 district | Urban | NR | 2009 | PW/RD | ANC based | 334 | Quant | Cross-sectional | IPTp/ ITN | IPTp use/ITN use |
| Mubyazi, 2010 [[75](#_ENREF_75)] | East Africa | Tanzania | >1 district | Rural | Perennial | 2005-2006 | PW/RD | ANC based | 240 | Qual | FGD/interview | IPTp/ ITN | ANC access/IPTp use & delivery /ITN use |
| Gikandi, 2008 [[76](#_ENREF_76)] | East Africa | Kenya | >1 district | Rural | Perennial/SP | 2006-2007 | PW/RD | Population based | 976 | Quant | Cross-sectional | IPTp/ ITN | IPTp 1/ITN use |
| Kiwuwa, 2008 [[77](#_ENREF_77)] | East Africa | Uganda | 1 district | Rural | Perennial/SP | 2005 | RD5 | Community based | 769 | Quant | Cross-sectional | IPTp/ ITN | ANC attendance/IPTp use/ITN use |
| Onyeaso, 2007 [[78](#_ENREF_78)] | West Africa | Nigeria | 1 district | Urban | NR | 2006 | HW | ANC based | 497 | Quant | Cross-sectional | IPTp/ ITN | IPTp knowledge/ITN delivery |
| Brentlinger, 2007 [[79](#_ENREF_79)] | East Africa | Mozambique | >1 district | Urban/ rural | Perennial | 2003-2004 | PW/HW | ANC based | 7911 | Mixed | Survey/interview | IPTp/ ITN | ANC access/IPTp use/IPTp delivery |
| Van Eijk, 2005 [[80](#_ENREF_80)] | East Africa | Kenya | 1 district | Rural | Perennial | 2002 | RD12 | Community based | 635 | Quant | Cross-sectional | IPTp/ ITN | ANC attendance/ timing/IPTp use/ITN use |
| Guyatt, 2004 [[81](#_ENREF_81)] | East Africa | Kenya | >1 districts | Urban/ rural | Perennial/SP | 2001 | PW/RD12 | Community based | 1814 | Quant | Cross-sectional | IPTp/ ITN | ANC timing/visits/IPTp use/ITN use |
| Nganda, 2004 [[82](#_ENREF_82)] | East Africa | Tanzania | 1 district | Rural | Perennial | 2003 | RD | Hospital based | 293 | Quant | Cross-sectional | IPTp/ ITN | IPTp 1/ITNuse |

Abbreviations:

NR: Not reported

Scale: 1 district, >1 district, National

Sample: Population based = National or >1 district, Community based = 1 district or community survey

Transmission setting: Perennial (endemic), Perennial/SP (with Seasonal Peaks)

Target population: PW = pregnant women, 15-49 yrs, RD = recently delivered (number denotes months since delivery), HW = health workers

Primary outcome: IPTp use (means dose is unspecified)

Table S2.2. Characteristics of intervention studies.

| **Author** | **Year** | **Country** | **Objective** | **Scale** | **Time Frame** | **Target Group** | **N** | **IPTp &/or ITNs** | **Intervention** | **Level of Subsidy** | **Voucher or Net** | **Study Design** | **Type of Control** | **Primary Outcome** |
| --- | --- | --- | --- | --- | --- | --- | --- | --- | --- | --- | --- | --- | --- | --- |
| **IPTp - Community based distribution** | | | | | | | | | | | | | | |
| Msyamboza [[83](#_ENREF_83)] | 2009 | Malawi | Assess the impact of community based distribution of IPTp on uptake | 1 district | 2002-2004 | PW | 1752 | IPTp | Community Based Distribution |  |  | Quasi-experimental | Control site | ANC attendance/IPTp 2 doses |
| Ndyomugyenyi [[84](#_ENREF_84)] | 2009 | Uganda | Evaluate the impact of community-directed drug distributors (CDDs) delivering IPTp on uptake | >1 district | 2007-2008 | PW | 926 | IPTp | Community Based Distribution |  |  | Quasi-experimental | Control site | ANC attendance/IPTp 1 dose/IPTp 2 doses |
| Mbonye [[85](#_ENREF_85)] | 2007 | Uganda | Assess the impact of community based distribution of IPTp on uptake | 1 district | 2003-2005 | PW | 2785 | IPTp | Community Based Distribution |  |  | Quasi-experimental | Control site | ANC attendance/IPTp 2 doses/ITN use |
| **IPTp - Educational & promotional campaigns** | | | | | | | | | | | | | | |
| Gies [[30](#_ENREF_30)] | 2009 | Burkina Faso | Evaluate the effect of a promotional campaign for women on ANC use and IPTp coverage | 1 district | 2003-2006 | PW | 2766 | IPTp | Educational & Promotional Campaigns |  |  | Quasi-experimental | Control site | ANC attendance/IPTp 2 doses |
| Ouma [[36](#_ENREF_36)] | 2007 | Kenya | Evaluate the impact of HW training on delivery & use of IPTp | 1 district | 2005 | RD 1-4 | 726 | IPTp | Educational & Promotional Campaigns |  |  | Quasi-experimental | Before/After | IPTp 2 doses |
| **IPTp/ITN - Community based distribution/Campaign distribution** | | | | | | | | | | | | | | |
| Okeibunor [[86](#_ENREF_86)] | 2011 | Nigeria | Increase access to malaria prevention (IPT/ITN) among pregnant women | >1 district | 2007-2010 | PW | 2652 | IPTp/ITN | Campaign Distribution/Community based distribution | Free | Net | Quasi-experimental | Control site | ANC attendance/IPTp 2 doses/ITN use |
| **ITN - Campaign distribution** | | | | | | | | | | | | | | |
| Thwing [[87](#_ENREF_87)] | 2011 | Senegal | Evaluate the progress of the National distribution campaign towards universal coverage | National | 2009-2010 | PW | NR | ITN | Campaign Distribution | Free | Net | Cross-sectional | Before/After | ITN use |
| Khatib [[88](#_ENREF_88)] | 2008 | Tanzania | Evaluate impact of delivery channels on ITN coverage and use | 1 district | 2006 | Infants | 484 | ITN | Campaign Distribution | Subsidised/Free | Net/Voucher | Cross-sectional | Comparison | ITN use/Source of ITN |
| Ahmed [[89](#_ENREF_89)] | 2010 | Uganda | Evaluated the effect of subsidised ITN distribution on use among the poorest women | 1 district | 2009 | PW | NR | ITN | Campaign Distribution | Subsidised | Net | Cross-sectional | Comparison | ITN use |
| **ITN - ANC distribution** | | | | | | | | | | | | | | |
| Marchant [[90](#_ENREF_90)] | 2010 | Tanzania | Evaluate the impact of the National Voucher Scheme on equity | National | 2007 | PW/RD 12 | 2034 | ITN | ANC Distribution | Subsidised | Voucher | Cross-sectional | N/A | Equity of ITN use |
| Beiersmann [[54](#_ENREF_54)] | 2010 | Burkina Faso | Qualitative exploration of provider perceptions and acceptability ITN delivery mechanisms | 1 district | 2007 | HW | 60 | ITN | ANC Distribution | Subsidised/Free | Net | FGD/interviews | N/A | ITN delivery |
| Hanson [[91](#_ENREF_91)] | 2009 | Tanzania | Evaluate the impact of the National Voucher Scheme on coverage to pregnant women | National | 2004-2006 | PW | 2070 | ITN | ANC Distribution | Subsidised | Voucher | Cross-sectional | Time comparison | ITN use/Voucher use |
| Pettifor [[92](#_ENREF_92)] | 2009 | DRC | Evaluate the impact of ITNs delivered free at ANC on ITN use | 1 district | 2005 | PW | 362 | ITN | ANC Distribution | Free | Net | Cohort | Baseline/ 6mths | ITN use |
| Muller [[93](#_ENREF_93)] | 2008 | Burkina Faso | Evaluate effect of free ITN distribution at ANC with or w/out social marketing on ITN use | 1 district | 2006-2007 | PW | 384 | ITN | ANC Distribution | Subsidised/Free | Net | Cross-sectional | Baseline/1yr | ITN use |
| Kweku [[61](#_ENREF_61)] | 2007 | Ghana | Examine pilot ITN voucher scheme delivered at ANC and determine barriers to access | >1 district | 2004-2005 | PW | 51658 | ITN | ANC Distribution | Subsidised | Voucher | Cross-sectional | N/A | Voucher use |
| Guyatt [[94](#_ENREF_94)] | 2003 | Kenya | Assess the impact of free ITN distribution at ANC on ITN use | >1 district | 2002 | PW | 294 | ITN | ANC Distribution | Free | Net | Cross-sectional | Baseline/1yr | ITN use |
| **ITN – Community based distribution** | | | | | | | | | | | | | | |
| Nonaka, 2012 [[95](#_ENREF_95)] | West Africa | Niger | Evaluate the impact of  community-based net distribution strategy  on the coverage and use of ITN | 1 district | 2010 | PW | 134 | ITN | Community based distribution | Subsidised | Net | Cross-sectional | Comparison | ITN use |
| **ITN - Social marketing & subsidies** | | | | | | | | | | | | | | |
| PSI Kenya [[96](#_ENREF_96)] | 2008 | Kenya | Assess the impact of social marketing campaign and subsidized ITNs on coverage and use | National | 2003-2007 | PW | 320 | ITN | Social Marketing & Subsidies | Subsidised | Net | Cross-sectional | Baseline/2yr/4yr | ITN use |
| PSI Madagascar [[97](#_ENREF_97)] | 2009 | Madagascar | Assess the impact of social marketing campaign and subsidized ITNs on coverage and use | National | 2004-2006 | 15-49yrs | 2920 | ITN | Social Marketing & Subsidies | Subsidised | Net | Cross-sectional | Baseline/2yr | ITN use |
| PSI Burundi [[98](#_ENREF_98)] | 2007 | Burundi | Assess the impact of social marketing campaign and subsidized ITNs on coverage and use | National | 2006-2007 | PW | 1332 | ITN | Social Marketing & Subsidies | Subsidised | Net | Cross-sectional | Baseline/1yr | ITN use |

Abbreviations:

Scale: 1 district, >1 district, National

Target Population: PW=pregnant women, 15-49yrs, RD=recently delivered (number denotes months since delivery)

**References**

1. Amoran OE, Ariba AA, Iyaniwura CA (2012a) Determinants of intermittent preventive treatment of malaria during pregnancy (IPTp) utilization in a rural town in Western Nigeria. Reproductive Health 9: (13 August 2012)-(2013 August 2012).

2. Arulogun OS, Okereke CC (2012) Knowledge and practices of intermittent preventive treatment of malaria in pregnancy among health workers in a southwest local government area of Nigeria. Journal of Medicine and Medical Sciences 3: 415-422.

3. Diala C, Pennas T, Choi P, Rogers S (2012) Barriers to uptake of malaria prevention and treatment during pregnancy in Cross River and Nasawara States, Nigeria.

4. Iliyasu Z, Gajida AU, Galadanci HS, Abubakar IS, Baba AS, et al. (2012) Adherence to intermittent preventive treatment for malaria in pregnancy in urban Kano, northern Nigeria. Pathogens and Global Health 106: 323-329.

5. Mubyazi GM, Bloch P, Byskov J, Magnussen P, Bygbjerg IC, et al. (2012) Supply-related drivers of staff motivation for providing intermittent preventive treatment of malaria during pregnancy in Tanzania: evidence from two rural districts. Malaria Journal 11: (15 February 2012)-(2015 February 2012).

6. Mutagonda R, Kamuhabwa AAR, Massawe S, Mpembeni R (2012) Intermittent preventive therapy and treatment of malaria during pregnancy: a study of knowledge among pregnant women in Rufiji District, Southern Tanzania. Tropical Journal of Pharmaceutical Research 11: 835-845.

7. Namusoke F, Ntale M, Wahlgren M, Kironde F, Mirembe F (2012) Validity of self-reported use of sulphadoxine-pyrimethamine intermittent presumptive treatment during pregnancy (IPTp): a cross-sectional study. Malaria Journal 11: (5 Se-(5 Se.

8. Onoka CA, Hanson K, Onwujekwe OE (2012a) Low coverage of intermittent preventive treatment for malaria in pregnancy in Nigeria: demand-side influences. Malaria Journal 11: (23 March 2012)-(2023 March 2012).

9. Onoka CA, Onwujekwe OE, Hanson K, Uzochukwu BS (2012b) Sub-optimal delivery of intermittent preventive treatment for malaria in pregnancy in Nigeria: influence of provider factors. Malaria Journal 11: (7 Se-(7 Se.

10. Onwujekwe OC, Soremekun RO, Uzochukwu B, Shu E, Onwujekwe O (2012) Patterns of case management and chemoprevention for malaria-in-pregnancy by public and private sector health providers in Enugu state, Nigeria. BMC Research Notes 5: 211.

11. Ansah-Ofei A, Mwini-Nyaledzigbor PP, Affram CK (2011) Improving the quality of life of pregnant women using the intermittent preventive treatment with sulfadoxine pyrimethamine (IPT-SP): experiences from Dangme West District, Ghana. Journal of Ghana Science Association 13: 88-102.

12. d'Almeida TC, Agboton-Zoumenou MA, Garcia A, Massougbodji A, Briand V, et al. (2011) Field evaluation of the intermittent preventive treatment of malaria during pregnancy (IPTp) in Benin: evolution of the coverage rate since its implementation. Parasites and Vectors 4: 108.

13. Donkor ES, Asiedua E (2011) Knowledge, practices and challenges of intermittent malaria preventive (IPT) treatment during pregnancy in Ghana. Africa Journal of Nursing and Midwifery 13: 34-45.

14. Faye A, Manga NM, Seck I, Niang K, Leye MM, et al. (2011) Access to intermittent preventive treatment (IPT) in a situation of abolition of user's fee: role of economic welfare. Bulletin de la Societe de Pathologie Exotique.

15. Nduka FO, Nwosu E, Oguariri RM (2011) Evaluation of the effectiveness and compliance of intermittent preventive treatment (IPT) in the control of malaria in pregnant women in south eastern Nigeria. Annals of Tropical Medicine and Parasitology 105: 599-605.

16. Tutu EO, Browne E, Lawson B (2011) Effect of sulphadoxine-pyrimethamine on neonatal birth weight and perceptions on its impact on malaria in pregnancy in an intermittent preventive treatment programme setting in Offinso District, Ghana. International Health.

17. Smith PL, Antwi GD, Jones C, Amoako E, Adjei RO, et al. (2011) Intermittent screening and treatment versus intermittent preventive treatment of malaria in pregnancy: provider knowledge and acceptability. PLoS ONE 6: e24035.

18. Gross K, Alba S, Schellenberg J, Kessy F, Mayumana I, et al. (2011) The combined effect of determinants on coverage of intermittent preventive treatment of malaria during pregnancy in the Kilombero Valley, Tanzania. Malaria Journal 10: 140.

19. Ndyomugyenyi R, Katamanywa J (2010) Intermittent preventive treatment of malaria in pregnancy (IPTp): do frequent antenatal care visits ensure access and compliance to IPTp in Ugandan rural communities? Transactions of the Royal Society of Tropical Medicine and Hygiene 104: 536-540.

20. Sangare LR, Stergachis A, Brentlinger PE, Richardson BA, Staedke SG, et al. (2010a) Determinants of use of intermittent preventive treatment of malaria in pregnancy: Jinja, Uganda. PLoS ONE 5: e15066.

21. Mbonye AK, Hansen KS, Wamono F, Magnussen P (2010) Integration of malaria and HIV/AIDS prevention through the private sector in Uganda. International Health 2: 52-58.

22. Sande JH, Kaseje D, Nyapada L, Owino VO (2010) Fear of being tested for HIV at ANC clinics associated with low uptake of intermittent preventive treatment (IPT) of malaria among pregnant women attending Bondo District Hospital, Western Kenya. East African Journal of Public Health 7: 92-96.

23. Grietens KP, Gies S, Coulibaly SO, Ky C, Somda J, et al. (2010) Bottlenecks for high coverage of intermittent preventive treatment in pregnancy: the case of adolescent pregnancies in rural Burkina Faso. PLoS ONE 5: e12013.

24. Antwi GD (2010) Factors influencing the uptake of intermittent preventive treatment of malaria in pregnancy in the Bosomtwe district of Ghana: Department of Community Health, School of Medical Sciences, K.N.U.S.T., Kumasi, Ghana.

25. Smith LA, Jones C, Adjei RO, Antwi GD, Afrah NA, et al. (2010) Intermittent screening and treatment versus intermittent preventive treatment of malaria in pregnancy: user acceptability. Malaria Journal 9: 18.

26. Akinleye SO, Falade CO, Ajayi IO (2009) Knowledge and utilization of intermittent preventive treatment for malaria among pregnant women attending antenatal clinics in primary health care centers in rural southwest, Nigeria: a cross-sectional study. BMC Pregnancy and Childbirth 9: 28.

27. Takem EN, Achidi EA, Ndumbe PM (2009) Use of intermittent preventive treatment for malaria by pregnant women in Buea, Cameroon. Acta Tropica 112: 54-58.

28. Acquah AAK (2009) Controlling malaria in pregnancy: investigating the factors that influence the uptake of intermittent preventive treatment services in the Nzema-East District, Ghana: Department of Community Health, School of Medical Sciences, College of Health Sciences, Kwame Nkrumah University of Science and Technology.

29. Klebi GW (2009) Factors accounting for the decline in IPTp2/IPTp3 among pregnant mothers in the Keta municipality - Ghana: School of Graduate Studies, Kwame Nkrumah University of Science and Technology, Kumasi, Ghana.

30. Gies S, Coulibaly SO, Ky C, Ouattara FT, Brabin BJ, et al. (2009) Community-based promotional campaign to improve uptake of intermittent preventive antimalarial treatment in pregnancy in Burkina Faso. American Journal of Tropical Medicine and Hygiene 80: 460-469.

31. Adjei DJD (2009) Factors affecting the intermittent preventive therapy of malaria in pregnancy programme in the Ejisu-Juabeng municipality: School of Graduate Studies, Kwame Nkrumah University of Science and Technology.

32. Brabin L, Stokes E, Dumbaya I, Owens S (2009) Rural Gambian women's reliance on health workers to deliver sulphadoxine-pyrimethamine as recommended intermittent preventive treatment for malaria in pregnancy. Malaria Journal 8: 25.

33. Marchant T, Nathan R, Jones C, Mponda H, Bruce J, et al. (2008) Individual, facility and policy level influences on national coverage estimates for intermittent preventive treatment of malaria in pregnancy in Tanzania. Malaria Journal 7: 260.

34. Anders K, Marchant T, Chambo P, Mapunda P, Reyburn H (2008) Timing of intermittent preventive treatment for malaria during pregnancy and the implications of current policy on early uptake in north-east Tanzania. Malaria Journal 7: 79.

35. Mubyazi GM, Bygbjerg IC, Magnussen P, Olsen O, Byskov J, et al. (2008) Prospects, achievements, challenges and opportunities for scaling-up malaria chemoprevention in pregnancy in Tanzania: the perspective of national level officers. Malaria Journal 7: 135.

36. Ouma PO, van Eijk AM, Hamel MJ, Sikuku E, Odhiambo F, et al. (2007) The effect of health care worker training on the use of intermittent preventive treatment for malaria in pregnancy in rural western Kenya. Tropical Medicine and International Health 12: 953-961.

37. Launiala A, Honkasalo ML (2007) Ethnographic study of factors influencing compliance to intermittent preventive treatment of malaria during pregnancy among Yao women in rural Malawi. Trans R Soc Trop Med Hyg 101: 980-989.

38. Mbonye AK, Neema S, Magnussen P (2006a) Perceptions on use of sulfadoxine-pyrimethamine in pregnancy and the policy implications for malaria control in Uganda. Health Policy 77: 279-289.

39. Gates Malaria Partnership, Institute for Health R, Development D (2005) Evaluation of the intermittent preventive treatment component of the accelerated child survival and development project in the Gambia.

40. Mubyazi G, Bloch P, Kamugisha M, Kitua A, Ijumba J (2005) Intermittent preventive treatment of malaria during pregnancy: a qualitative study of knowledge, attitudes and practices of district health managers, antenatal care staff and pregnant women in Korogwe District, North-Eastern Tanzania. Malaria Journal 4: 31.

41. Holtz TH, Kachur SP, Roberts JM, Marum LH, Mkandala C, et al. (2004) Use of antenatal care services and intermittent preventive treatment for malaria among pregnant women in Blantyre District, Malawi. Tropical Medicine and International Health 9: 77-82.

42. Ashwood-Smith H, Coombes Y, Kaimila N, Bokosi M, Lungu K (2002) Availability and use of sulphadoxine-pyrimethamine (SP) in pregnancy in Blantyre District: A Safe Motherhood and Blantyre Integrated Malaria Initiative (BIMI) Joint Survey. Malawi Medical Journal 14: 8-11.

43. Aluko JO, Oluwatosin AO (2012) Utilization of insecticide treated nets during pregnancy among postpartum women in Ibadan, Nigeria: a cross-sectional study. BMC Pregnancy and Childbirth 12: (29 March 2012)-(2029 March 2012).

44. Amoran OE, Lawal KM, Jeminusi OA, Alabi AA, Oluwole FA (2012b) Determinants of uptake of insecticide treated nets among pregnant women in Ado-Odo Local Government area of Ogun State, Nigeria. Journal of Community and Health Education 2: 1000126.

45. Ankomah A, Adebayo SB, Arogundade ED, Anyanti J, Nwokolo E, et al. (2012) Determinants of insecticide-treated net ownership and utilization among pregnant women in Nigeria. BMC Public Health 12: (6 February 2012)-(2016 February 2012).

46. Auta A (2012) Demographic factors associated with insecticide treated net use among Nigerian women and children. North American Journal of Medical Sciences 4: 40-44.

47. Ambrose EE, Mazigo HD, Heukelbach J, Gabone O, Mwizamholya DL (2011) Knowledge, attitudes and practices regarding malaria and mosquito net use among women seeking antenatal care in Iringa, south-western Tanzania. Tanzania Journal of Health Research 13.

48. Okonta PI (2011) Utilization of insecticide-treated net during pregnancy in Delta-State: a survey of recently delivered mothers. Ebonyi Medical Journal 10.

49. Olajide FO, Afolabi OT, Olajide AO, Omisore AG, Omomuniniyi OA (2011) Challenges with the use of insecticide treated nets among pregnant women in Ife-Ijesha Zone, South Western Nigeria. Journal of Community Medicine and Primary Health Care 23: 79-86.

50. O'Meara WP, Smith N, Ekal E, Cole D, Ndege S (2011) Spatial distribution of bednet coverage under routine distribution through the public health sector in a rural district in Kenya. PLoS ONE 6: e25949.

51. Karunamoorthi K, Deboch B, Tafere Y (2010) Knowledge and practice concerning malaria, insecticide-treated net (ITN) utilization and antimalarial treatment among pregnant women attending specialist antenatal clinics. Journal of Public Health 18: 559-566.

52. Wagbatsoma VA, Aigbe EE (2010) ITN utilization among pregnant women attending ANC in Etsako West Lga, Edo State, Nigeria. Nigerian Journal of Clinical Practice 13: 144-148.

53. Sangare LR (2010b) Use of malaria prevention and control measures during pregnancy in Jinja, Uganda: University of Washington, Seattle, USA.

54. Beiersmann C, De AM, Tiendrebeogo J, Ye M, Jahn A, et al. (2010) Different delivery mechanisms for insecticide-treated nets in rural Burkina Faso: a provider's perspective. Malaria Journal 9: 352.

55. Musa OI, Salaudeen GA, Jimoh RO (2009) Awareness and use of insecticide treated nets among women attending ante-natal clinic in a northern state of Nigeria. Journal of the Pakistan Medical Association 59: 354-358.

56. Njoroge FK, Kimani VN, Ongore D, Akwale WS (2009) Use of insecticide treated bed nets among pregnant women in Kilifi District, Kenya. East African Medical Journal 86: 314-322.

57. Pettifor AE, Taylor E, Nku D, Duvall S, Tabala M, et al. (2008) Bed net ownership, use, and perceptions among women seeking antenatal care in Kinshasa, Democratic Republic of the Congo (DRC): opportunities for improved maternal and child health. BMC Public Health 8: 331.

58. Belay M, Deressa W (2008) Use of insecticide treated nets by pregnant women and associated factors in a pre-dominantly rural population in northern Ethiopia. Tropical Medicine and International Health 13: 1303-1313.

59. Hassan SEH, Malik EM, Okoued SI, Eltayeb EM (2008) Retention and efficacy of long-lasting insecticide-treated nets distributed in eastern Sudan: a two-step community-based study. Malaria Journal 7: 85.

60. Mnyika KS, Kabalimu TK, Mbaruku G (2006) Determinants of utilisation of mosquito bednets for malaria prevention among pregnant women in Kigoma urban district, western Tanzania. East African Journal of Public Health 3: 31-34.

61. Kweku M, Webster J, Taylor I, Burns S, Dedzo M (2007) Public-private delivery of insecticide-treated nets: a voucher scheme in Volta Region, Ghana. Malaria Journal 6: 14.

62. Population Services International (2006) Zambia (2005): Malaria TRaC Study examining the use of inecticide treated nets among pregnant women in Zambia. First Round.

63. Population Services International (2006) Burundi (2006): Determinants of the use of bed nets among mothers of children less than 5 years of age and pregnant women in Burundi. Bujumbura, Burundi.

64. Population Services International (2006) Rwanda (2006): Malaria TRaC Study evaluating the use of insecticide treated nets among pregnant women and children under 5 years of age. First Round. Kigali, Rwanda.

65. Van Geertruyden JP, Ntakirutimana D, Erhart A, Rwagacondo C, Kabano A, et al. (2005) Malaria infection among pregnant women attending antenatal clinics in six Rwandan districts. Tropical Medicine and International Health 10: 681-688.

66. Mbonye AK, Neema S, Magnussen P (2006b) Preventing malaria in pregnancy: a study of perceptions and policy implications in Mukono district, Uganda. Health Policy and Planning 21: 17-26.

67. Marchant T, Schellenberg JA, Edgar T, Nathan R, Abdulla S, et al. (2002) Socially marketed insecticide-treated nets improve malaria and anaemia in pregnancy in southern Tanzania. Tropical Medicine and International Health 7: 149-158.

68. Akaba GO, Otubu J, Agida ET, Onafowokan O (2013) Knowledge and utilization of malaria preventive measures among pregnant women at a tertiary hospital in Nigeria's federal capital territory. Nigerian Journal of Clinical Practice 16: 201-206.

69. De Allegri M, Louis VR, Tiendrebeogo J, Souares A, Ye M, et al. (2012) Moving towards universal coverage with malaria control interventions: achievements and challenges in rural Burkina Faso. International Journal of Health Planning and Management.

70. Hill J, Dellicour S, Bruce J, Ouma P, Smedley J, et al. (2013) Effectiveness of antenatal clinics to deliver intermittent preventive treatment and insecticide treated nets for the control of malaria in pregnancy in Kenya Plos ONE In press.

71. Bouyou-Akotet MK, Mawili-Mboumba DP, Kombila M (2013) Antenatal care visit attendance, intermittent preventive treatment and bed net use during pregnancy in Gabon. BMC Pregnancy and Childbirth 13: (26 February 2013)-(2026 February 2013).

72. Zere E, Kirigia JM, Duale S, Akazili J (2012) Inequities in maternal and child health outcomes and interventions in Ghana. BMC Public Health 12: 252.

73. Manirakiza A, Serdouma E, Djalle D, Soula G, Laganier R, et al. (2011) Relatively low prevalence of peripheral and placental Plasmodium infection at delivery in Bangui, Central African Republic. Journal of Tropical Medicine 2011: 434816.

74. Napoleon RP, Anyangu AS, Omolocan J, Ongus JR (2011) Preventing malaria during pregnancy: factors determining the use of insecticide-treated bednets and intermittent preventive therapy in Juba. Southern Sudan Medical Journal 4: 33-38.

75. Mubyazi GM, Bloch P, Magnussen P, Olsen OE, Byskov J, et al. (2010) Women's experiences and views about costs of seeking malaria chemoprevention and other antenatal services: a qualitative study from two districts in rural Tanzania. Malaria Journal 9: 54.

76. Gikandi PW, Noor AM, Gitonga CW, Ajanga AA, Snow RW (2008) Access and barriers to measures targeted to prevent malaria in pregnancy in rural Kenya. Tropical Medicine and International Health 13: 208-217.

77. Kiwuwa MS, Mufubenga P (2008) Use of antenatal care, maternity services, intermittent presumptive treatment and insecticide treated bed nets by pregnant women in Luwero district, Uganda. Malaria Journal 7: 44.

78. Onyeaso NC, Fawole AO (2007) Perception and practice of malaria prophylaxis in pregnancy among health care providers in Ibadan. African Journal of Reproductive Health 11: 60-69.

79. Brentlinger PE, Dgedge M, Correia MA, Rojas AJ, Saute F, et al. (2007) Intermittent preventive treatment of malaria during pregnancy in central Mozambique. Bulletin of the World Health Organization 85: 873-879.

80. van Eijk AM, Blokland IE, Slutsker L, Odhiambo F, Ayisi J, et al. (2005) Use of intermittent preventive treatment for malaria in pregnancy in a rural area of western Kenya with high coverage of insecticide-treated bed nets. Tropical Medicine and International Health 10: 1134-1140.

81. Guyatt HL, Noor AM, Ochola SA, Snow RW (2004) Use of intermittent presumptive treatment and insecticide treated bed nets by pregnant women in four Kenyan districts. Tropical Medicine and International Health 9: 255-261.

82. Nganda RY, Drakeley C, Reyburn H, Marchant T (2004) Knowledge of malaria influences the use of insecticide treated nets but not intermittent presumptive treatment by pregnant women in Tanzania. Malaria Journal 3: 42.

83. Msyamboza KP, Savage EJ, Kazembe PN, Gies S, Kalanda G, et al. (2009) Community-based distribution of sulfadoxine-pyrimethamine for intermittent preventive treatment of malaria during pregnancy improved coverage but reduced antenatal attendance in southern Malawi. Tropical Medicine and International Health 14: 183-189.

84. Ndyomugyenyi R, Tukesiga E, Katamanywa J (2009) Intermittent preventive treatment of malaria in pregnancy (IPTp): participation of community-directed distributors of ivermectin for onchocerciasis improves IPTp access in Ugandan rural communities. Transactions of the Royal Society of Tropical Medicine and Hygiene 103: 1221-1228.

85. Mbonye AK, Bygbjerg I, Magnussen P (2007) Intermittent preventive treatment of malaria in pregnancy: Evaluation of a new delivery approach and the policy implications for malaria control in Uganda. Health Policy 81: 228-241.

86. Okeibunor JC, Orji BC, Brieger W, Ishola G, Otolorin E, et al. (2011) Preventing malaria in pregnancy through community-directed interventions: evidence from Akwa Ibom State, Nigeria. Malaria Journal 10: 227.

87. Thwing JI, Perry RT, Townes DA, Diouf MB, Ndiaye S, et al. (2011) Success of Senegal's first nationwide distribution of long-lasting insecticide-treated nets to children under five - contribution toward universal coverage. Malaria Journal 10: (13 A-(13 A.

88. Khatib RA, Killeen GF, Abdulla SM, Kahigwa E, McElroy PD, et al. (2008) Markets, voucher subsidies and free nets combine to achieve high bed net coverage in rural Tanzania. Malaria Journal 7: 98.

89. Ahmed SM, Zerihun A (2010) Possession and usage of insecticidal bed nets among the people of Uganda: is BRAC Uganda Health Programme pursuing a pro-poor path? PLoS ONE 5.

90. Marchant T, Schellenberg D, Nathan R, Armstrong-Schellenberg J, Mponda H, et al. (2010) Assessment of a national voucher scheme to deliver insecticide-treated mosquito nets to pregnant women. Canadian Medical Association Journal 182: 152-156.

91. Hanson K, Marchant T, Nathan R, Mponda H, Jones C, et al. (2009) Household ownership and use of insecticide treated nets among target groups after implementation of a national voucher programme in the United Republic of Tanzania: plausibility study using three annual cross sectional household surveys. British Medical Journal 339: b2434.

92. Pettifor A, Taylor E, Nku D, Duvall S, Tabala M, et al. (2009) Free distribution of insecticide treated bed nets to pregnant women in Kinshasa: an effective way to achieve 80% use by women and their newborns. Tropical Medicine and International Health 14: 20-28.

93. Muller O, De AM, Becher H, Tiendrebogo J, Beiersmann C, et al. (2008) Distribution systems of insecticide-treated bed nets for malaria control in rural Burkina Faso: cluster-randomized controlled trial. PLoS ONE 3: e3182.

94. Guyatt H, Ochola S (2003) Use of bednets given free to pregnant women in Kenya. Lancet (British edition) 362: 1549-1550.

95. Nonaka D, Maazou A, Yamagata S, Oumarou I, Uchida T, et al. (2012) Distribution of subsidized insecticide-treated bed nets through a community health committee in Boboye Health District, Niger. Tropical Medicine and Health 40: 125-131.

96. Population Services International (2008) Kenya (2007): Malaria TRaC Study evaluating bed net ownership and use among pregnant women and children under 5 (years). Third Round. Nairobi, Kenya.

97. Population Services International (2009) Madagascar (2008): Malaria TRaC Study Evaluating the use of insecticide treated nets among pregnant women and mothers/caregivers of children younger than five years in endemic area. Third Round.

98. Population Services International (2007) Burundi (2007): Malaria TRaC Study evaluating bed net use among pregnant women and children under 5 years. First Round. Bujumbura, Burundi.
