# Supplementary material for: Factors Affecting the Delivery, Access, and Use of Interventions to Prevent Malaria in Pregnancy in Sub-Saharan Africa: A Systematic Review and Meta-Analysis
Source: PLoS Med. 2013 Jul 23;10(7):e1001488. doi: 10.1371/journal.pmed.1001488 (PMC3720261; doi:10.1371/journal.pmed.1001488)
Supplement: Table S4 — Barriers and facilitators to delivery, access, and use of IPTp and ITNs. Table S4.1. Barriers and facilitators to receipt of IPTp from the perspective of pregnant and recently delivered women. Table S4.2. Barriers and facilitators to ITN ownership and use from the perspective of pregnant and recently delivered women. Table S4.3. Barriers and facilitators to the delivery of IPTp from the healthcare provider perspective. Table S4.4. Barriers and facilitators to the delivery and use of ITNs from the healthcare provider perspective. (DOCX) [file pmed.1001488.s004.docx]

Tables S4. Barriers and facilitators to delivery, access and use of IPTp and ITNs.

Table S4.1. Barriers and facilitators to receipt of IPTp from the pregnant and recently delivered women perspective.*

|  | **Individual** | | **Social/Cultural/Household** | | **Environmental** | | **Health System** | |
| --- | --- | --- | --- | --- | --- | --- | --- | --- |
| **Pregnant women – Facilitators** | Qualitative | Quantitative | Qualitative | Quantitative | Qualitative | Quantitative | Qualitative | Quantitative |
| Acceptance of malaria prevention drugs | 3 | **1** |  |  |  |  |  |  |
| Trust in ANC staff to give good drugs | 5 | 1 |  |  |  |  |  |  |
| Believe SP is safe to use in pregnancy | 2 |  |  |  |  |  |  |  |
| SP is considered effective to prevent mip/improve birth outcomes | 1 |  |  |  |  |  |  |  |
| SP is considered effective to treat malaria | 2 |  |  |  |  |  |  |  |
| Very few or no side effects | 4 | 1 |  |  |  |  |  |  |
| Partners supportive of using antimalarials in pregnancy |  |  |  | 1 |  |  |  |  |
| Receiving information about IPTp from radio & ANC |  |  |  |  |  |  | 2 | 3 |
| Receiving information about IPTp from nurses and doctors |  |  |  |  |  |  |  | 2 |
| Dose is written in ANC card |  |  |  |  |  |  | 1 |  |
| **Pregnant women – Barriers** |  |  |  |  |  |  |  |  |
| Low knowledge of the benefits of IPTp | 4 | 5 |  |  |  |  |  |  |
| Fear of perceived side effects | 2 | 3 |  |  |  |  |  |  |
| Experienced side effects | 4 | 3 |  |  |  |  |  |  |
| Lack of awareness of timing & dosing of IPTp | 3 | 4 |  |  |  |  |  |  |
| Confusion about what drugs are safe to take in pregnancy | 1 | 2 |  |  |  |  |  |  |
| Perception that SP is strong & many cause miscarriages | 1 |  |  |  |  |  |  |  |
| Poor ANC attendance | 6 | 4 |  |  |  |  |  |  |
| Lack of awareness of what SP is used for | 2 | 3 |  |  |  |  |  |  |
| Needing to take drug home in order to eat, or buying elsewhere | 1 |  |  |  |  |  |  |  |
| Attend ANC/hospital only when sick [with malaria] | 1 |  |  |  |  |  |  |  |
| Having to purchase SP & supply water |  |  | 2 | 4 |  |  |  |  |
| Being advised to drink costly fluids (juice, soda) with SP |  |  | 1 |  |  |  |  |  |
| Commitments at home (child care & farming) |  |  | 2 |  |  |  |  |  |
| Adolescents & PG women not considered more vulnerable |  |  | 1 | 1 |  |  |  |  |
| Need husbands support or consent |  |  | 1 | 1 |  |  |  |  |
| User fees & penalties |  |  |  |  |  |  | 2 | 1 |
| Stock outs of SP |  |  |  |  |  |  | 2 | 6 |
| Not being offered SP by health worker |  |  |  |  |  |  | 1 | 6 |
| ANC cards not being updated properly |  |  |  |  |  |  | 1 |  |
| Need to share cup to take medication |  |  |  |  |  |  |  | 3 |
| Referred to laboratory |  |  |  |  |  |  | 1 |  |
| Taking folic acid and iron sulphate supplementation |  |  |  |  |  |  | 1 | 1 |

* Numbers refer to the number of qualitative and quantitative studies included in this review that report the barrier or facilitator

Abbreviations: IPTp, intermittent preventive treatment; ANC, antenatal clinic; SP, sulphadoxine pyrimethamine; PG, primigravida

Table S4.2. Barrier and facilitators to the delivery of IPTp from the healthcare provider perspective.*

|  | **Individual** | | **Organisational** | | **Health System** | | **Non-Health System** | |
| --- | --- | --- | --- | --- | --- | --- | --- | --- |
| **Healthcare provider – Facilitators** | Qualitative | Quantitative | Qualitative | Quantitative | Qualitative | Quantitative | Qualitative | Quantitative |
| High knowledge of IPTp strategy | 3 | 2 |  |  |  |  |  |  |
| High knowledge of dosing & timing | 1 | 3 |  |  |  |  |  |  |
| Perception that IPTp is beneficial to mother & child | 3 |  |  |  |  |  |  |  |
| Knowledge that SP is for malaria prevention | 2 | 1 |  |  |  |  |  |  |
| Did not feel that side effects were inhibitory | 1 |  |  |  |  |  |  |  |
| Safe drinking water was available for free |  |  |  | 1 |  |  |  |  |
| IPTp was administered according to DOT |  |  |  |  | 1 | 1 |  |  |
| IPTP was administered free of charge |  |  |  |  |  | 2 |  |  |
| SP was available at the health facility |  |  |  |  | 2 | 1 |  |  |
| Staff were given training on IPTp |  |  |  |  | 2 |  |  |  |
| Staff supervised whilst administering SP |  |  |  |  |  | 1 |  |  |
| **Healthcare provider – Barriers** |  |  |  |  |  |  |  |  |
| Confusion about timing & dosing of IPTp | 4 | 4 |  |  |  |  |  |  |
| Low knowledge regarding IPTp strategy | 3 | 3 |  |  |  |  |  |  |
| Low knowledge of the side effects & contraindications of SP | 1 | 3 |  |  |  |  |  |  |
| SP distributed regardless of gestational age | 2 | 1 |  |  |  |  |  |  |
| Imprecise estimation of gestational age | 1 |  |  |  |  |  |  |  |
| Perception that women will not take SP on empty stomach | 2 |  |  |  |  |  |  |  |
| Staff too busy to distribute SP |  |  | 1 |  |  |  |  |  |
| Lack of water cups at facility |  |  | 4 |  |  |  |  |  |
| Health instructions about malaria not given in local language |  |  | 1 |  |  |  |  |  |
| Variation in information about IPTp delivery given to staff |  |  |  | 1 |  |  |  |  |
| Guidelines on IPTp not present at facility |  |  | 1 | 1 |  |  |  |  |
| IPTp delivered based on restrictive schedule |  |  |  |  | 1 |  |  |  |
| SP stock outs |  |  |  |  | 6 | 2 |  |  |
| Lack of supervision & monitoring of IPTp delivery |  |  |  |  | 4 |  |  |  |
| User fees for IPTp |  |  |  |  | 2 |  |  |  |
| Lack of recent IPTp training |  |  |  |  | 2 | 1 |  |  |
| Private healthcare facilities dispensing other malaria drugs for IPTp |  |  |  |  | 2 |  |  |  |
| Incompatibilities of IPTp with other health programs |  |  |  |  | 1 |  |  |  |
| ANC facilities not easily accessible for women |  |  |  |  | 2 |  |  |  |
| Delivery impeded by lack of basic facilities, training and remuneration |  |  |  |  |  | 1 |  |  |
| Water shortages at facilities |  |  |  |  |  |  | 2 | 2 |
| Negative media coverage for SP |  |  |  |  |  |  | 2 | 1 |
| Women prefer other malaria drugs to SP |  |  |  |  |  |  | 1 |  |
| Complaints of side effects from SP |  |  |  |  |  |  | 2 |  |
| Late ANC attendance by women |  |  |  |  |  |  | 3 | 1 |
| Women not returning for subsequent ANC visits |  |  |  |  |  |  | 1 | 1 |
| Women do not feel sick & do not want medication |  |  |  |  |  |  | 1 | 1 |
| DOT not considered important |  |  |  |  |  |  | 1 |  |
| Women had no money to buy water |  |  |  |  |  |  |  | 1 |

- Numbers refer to the number of qualitative and quantitative studies included in this review that report the barrier or facilitator

Abbreviations: IPTp, intermittent preventive treatment; ANC, antenatal clinic; SP, sulphadoxine pyrimethamine; DOT, directly observed therapy

Table S4.3. Barriers and facilitators to ITN ownership and use from the pregnant and recently delivered women perspective.*

|  | **Individual** | | **Social/Cultural/Household** | | **Environmental** | | **Health System** | |
| --- | --- | --- | --- | --- | --- | --- | --- | --- |
| **Pregnant women – Facilitators** | Qualitative | Quantitative | Qualitative | Quantitative | Qualitative | Quantitative | Qualitative | Quantitative |
| Knowledge of vector based transmission |  | 2 |  |  |  |  |  |  |
| Knowledge & awareness of ITN benefits for mother & child | 1 | 3 |  |  |  |  |  |  |
| **Pregnant women – Barriers** |  |  |  |  |  |  |  |  |
| Feeling hot & uncomfortable under the ITN | 2 | 11 |  |  |  |  |  |  |
| Inconvenience of putting it up & down each night |  | 6 |  |  |  |  |  |  |
| Not used to using ITNs | 1 |  |  |  |  |  |  |  |
| Fear of the chemicals on the ITNs | 2 | 1 |  |  |  |  |  |  |
| Belief that ITN’s don’t prevent malaria |  | 4 |  |  |  |  |  |  |
| Can’t afford the cost of ITNs |  |  | 2 | 9 |  |  |  |  |
| Lack of community support from husband &/or community |  |  | 2 | 4 |  |  |  |  |
| Don’t like the style or colour of ITN available |  |  | 1 | 1 |  |  |  |  |
| Unfair community distribution |  |  | 1 |  |  |  |  |  |
| Place of residence |  |  |  |  |  | 7 |  |  |
| Seasonality (hot weather) |  |  |  |  | 1 | 1 |  |  |
| Perception that there are no mosquitoes in the area |  |  |  |  | 1 | 2 |  |  |
| Unavailability of ITNs |  |  |  |  |  |  | 2 | 4 |
| ITN stock outs |  |  |  |  |  |  | 1 |  |
| Travel to collect ITNs with voucher |  |  |  |  |  |  | 1 |  |
| Variation in voucher top-up costs |  |  |  |  |  |  | 1 |  |
| Inaccessibility to free ITNS |  |  |  |  |  |  |  | 1 |

* Numbers refer to the number of qualitative and quantitative studies included in this review that report the barrier or facilitator

Abbreviations: ITN, insecticide treated net

Table S4.4. Barrier and facilitators to the delivery and use of ITNs from the healthcare provider perspective.*

|  | **Individual** | | **Organisational** | | **Health System** | | **Non-health System** | |
| --- | --- | --- | --- | --- | --- | --- | --- | --- |
| **Healthcare provider – Facilitators** | Qualitative | Quantitative | Qualitative | Quantitative | Qualitative | Quantitative | Qualitative | Quantitative |
| Perception that free distribution of ITNs is beneficial | 1 |  |  |  |  |  |  |  |
| Recommending ITN use to pregnant women |  | 1 |  |  |  |  |  |  |
| **Healthcare provider – Barriers** |  |  |  |  |  |  |  |  |
| Healthcare providers imposing eligibility criteria for vouchers | 1 |  |  |  |  |  |  |  |
| Vouchers not available |  |  |  |  | 1 |  |  |  |
| Stock out of ITNs |  |  |  |  | 2 |  |  |  |
| Cost of ITNs |  |  |  |  | 1 |  | 2 |  |
| ITNs cause burning eyes, perspiration and restrained mobility |  |  |  |  |  |  | 1 |  |

* Numbers refer to the number of qualitative and quantitative studies included in this review that report the barrier or facilitator

Abbreviations: ITN, insecticide treated net
