# Supplementary material for: Factors Affecting the Delivery, Access, and Use of Interventions to Prevent Malaria in Pregnancy in Sub-Saharan Africa: A Systematic Review and Meta-Analysis
Source: PLoS Med. 2013 Jul 23;10(7):e1001488. doi: 10.1371/journal.pmed.1001488 (PMC3720261; doi:10.1371/journal.pmed.1001488)
Supplement: Text S2 — Meta-analysis of determinants of IPTp and ITN use in pregnancy. (PDF) [file pmed.1001488.s006.pdf]

Text S2: Meta-analysis of determinants of IPTp and ITN use in pregnancy

We conducted a meta-analysis of data available from studies on determinants of intermittent preventive treatment (IPTp) and insecticide treated net (ITN) use in pregnancy. Odds ratios (ORs) were used to represent effect size and data were extracted from studies in the following hierarchy: raw data (numbers) were used in preference to unadjusted ORs and 95% CI; unadjusted ORs were used where raw numbers were not provided; adjusted ORs were used if unadjusted ORs or raw counts were not provided. Several studies reported a cluster design, but it was not always clear if the design was taken into account when the results were presented. For this reason the use of a cluster design as provided by the studies is indicated in the meta-analysis forest plots and notes, in addition to the use of adjusted (by multivariate analysis) ORs. If studies contributed data for "1+ doses" and "2+ doses" of IPTp, the data for "1+ doses" was removed to avoid over-representation of that study population in the analysis. A random effects model was used to provide a summary OR estimate of effect using the approach by DerSimonian and Laird. The random effects model recognises that the studies are a sample of all potential studies and incorporates a between study component to the estimate of variability. We present the ORs with 95% confidence intervals (CI), the I^2 and 95% CI to describe the percentage of the variability in the summary OR that is attributable to between study heterogeneity and the P-value for the Z-statistic. The Z-value tests the null-hypothesis that the effect of an OR is 1. We conducted subgroup analysis and considered the following factors for IPTp: number of SP doses (1+ or 2+), location of enrolment (community or clinic), study population (postpartum women or a mixed population of postpartum and pregnant women or pregnant women only), and study country. For the subgroup analysis for ITNs we considered the following factors: type of net (ITN or net), definition of net use (last night or during pregnancy), location of enrolment (community or clinic), study population (postpartum women or a mixed population of postpartum and pregnant women or pregnant women only), and study country. To assess a potential effect of study quality on the examined associations, we assigned studies a score based on the quality assessment (supporting information 3), with a score of three or more failures for the categories examined indicating moderate-to-poor study quality. We included the effect of study quality in sub group analyses for both IPTp and ITNs for all exposures. Meta-analyses were performed using Stata version 12 for the presentation of the main results and Comprehensive Meta-analysis (Biostat, http://www.meta-analysis.com/) for the subgroup analysis.

The following determinants were examined

	IPTp		ITN	
	Meta-analysis	Sub group Analysis	Meta-analysis	Sub group Analysis
Socioeconomic status	Figure S2.1 (page 2)	Table S2.1 (page 3)	Figure S2.13 (page 24)	Table S2.13 (page 25)
Education	Figure S2.2 (page 4)	Table S2.2 (page 5)	Figure S2.14 (page 26)	Table S2.14 (page 27)
Residence	Figure S2.3 (page 6)	Table S2.3 (page 7)	Figure S2.15 (page 28)	Table S2.15 (page 29)
Employment	Figure S2.4 (page 8)	Table S2.4 (page 9)	Figure S2.16 (page 30)	Table S2.16 (page 31)
Marital status	Figure S2.5 (page 10)	Table S2.5 (page 11)	Figure S2.17 (page 32)	Table S2.17 (page 33)
Age	Figure S2.6 (page 12)	Table S2.6 (page 13)	Figure S2.18 (page 34)	Table S2.18 (page 35)
Parity/gravidity	Figure S2.7 (page 14)	Table S2.7 (page 15)	Figure S2.19 (page 36)	Table S2.19 (page 37)
Knowledge malaria	Figure S2.8 (page 16)	Table S2.8 (page 16)	Figure S2.20 (page 38)	Table S2.20 (page 39)
Distance ANC	Figure S2.9 (page 17)	Table S2.9 (page 18)	Not enough data	
Timing start ANC	Figure S2.10 (page 19)	Table S2.10 (page 20)	Not enough data	
# of ANC visits	Figure S2.11 (page 21)	Table S2.11 (page 22)	Not enough data	
Other prevention	Figure S2.12 (page 23)	Table S2.12 (page 23)	Figure S2.21 (page 40)	Table S2.21 (page 40)

Figure S2.1: **IPTp and socioeconomic status: higher vs. lower wealth in quintiles of quartiles**

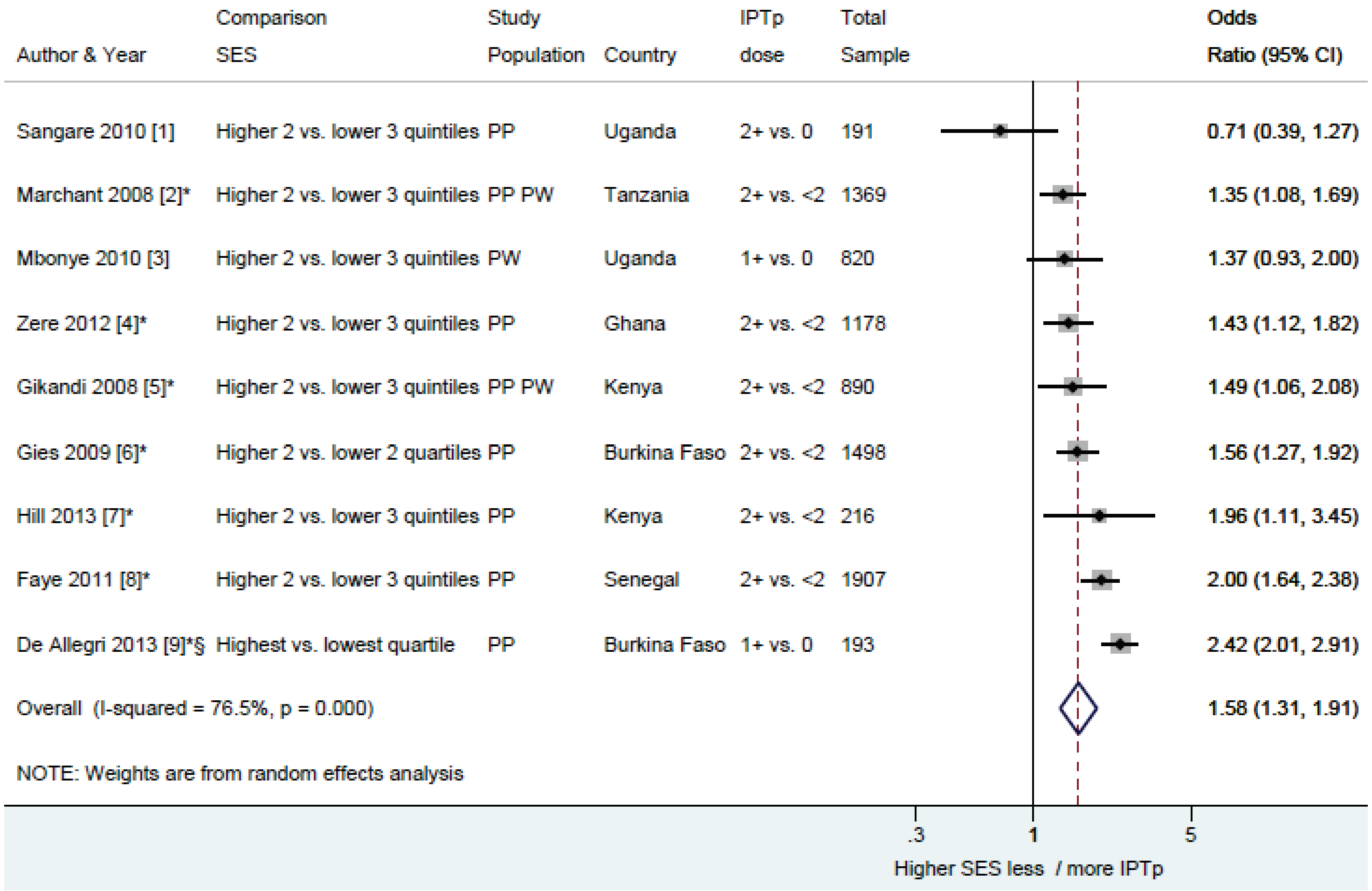

Abbreviations: CI: confidence interval. IPTp: intermittent preventive treatment pregnant women. PP: postpartum women. PW: pregnant women.
The grey boxes indicate the weight for each study.
* Studies using cluster design. The studies by Marchant 2008, Gikandi 2008, and Gies 2009 took the study design into account in the analysis [2,3,5]. For Faye 2011 and Zere 2012 this was not clear [4,8].
§ Studies where an adjusted OR was used
Summary estimate: p <0.001. I^2 =77%, 95% CI 55-88%. Total sample size: 8262 women in 9 studies

Notes: The study population of Sangare 2010 included postpartum women who had at least 2 ANC visits which qualified for IPTp [1]. The study population of De Allegri 2013 made at least one ANC visit [9]. In the study by Gies 2009 et al, only primi- and secundigravidae participated [6].

***Table S2.1*: IPTp and socioeconomic status sub-group analyses: Groups with higher socio-economic status vs. lower economic status**

						Within subgroups		Between subgroup
**Covariate**	**Subgroup**	**N studies**	**Odds Ratio**	**Lower limit 95% CI**	**Upper limit 95% CI**	**p-value†**	I^2	**p-value†**
Number of SP doses	1+	2	2.00	1.43	2.81	0.019	81.7%	0.139
	2+	7	1.49	1.23	1.81	0.009	64.6%	
Location enrolment	All recruited from Community							
Study population	Postpartum women	6	1.67	1.34	2.09	<0.001	81.6%	0.469
	Pregnant and postpartum	3	1.45	1.06	1.99	0.797	0.0%	
Country	Burkina Faso	2	1.95	1.23	3.09	0.002	89.4%	0.640
	Ghana	1	1.44	0.74	2.79			
	Kenya	2	1.67	0.97	2.87	0.419	0.0%	
	Senegal	1	1.99	1.04	3.80			
	Tanzania	1	1.36	0.70	2.62			
	Uganda	2	1.09	0.63	1.88	0.013	83.7%	
Study quality	Good	5	1.56	1.18	2.06	<0.001	85.9%	0.872
	Moderate-to-low	4	1.61	1.20	2.17	0.150	43.6%	

Abbreviations: CI: confidence interval. IPTp: intermittent preventive treatment pregnancy. SP: Sulfadoxine-pyrimethamine.
†p-value Q-statistic (assessment of heterogeneity)

Figure S2.2: **IPTp and education: More education vs. less**

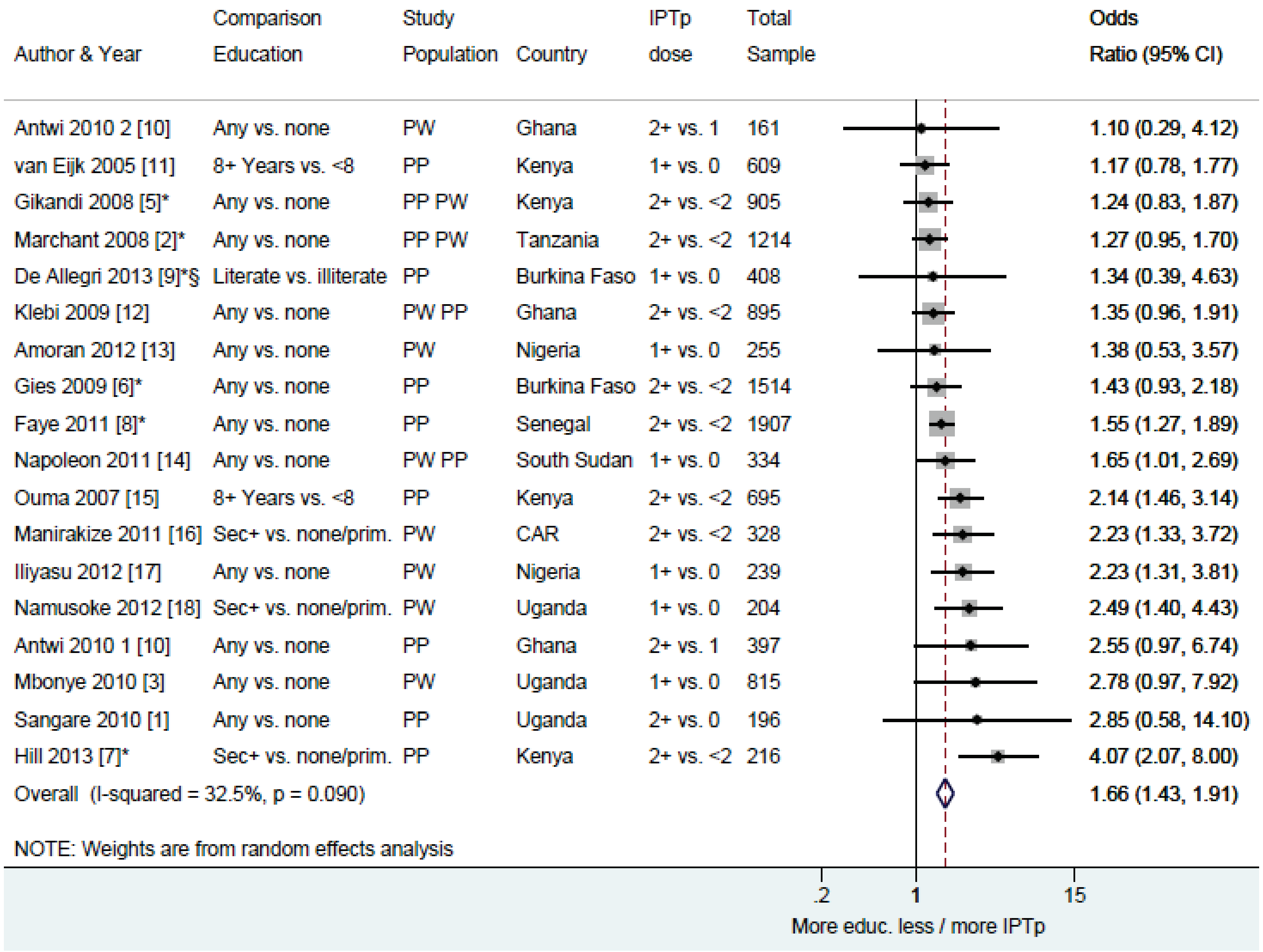

Abbreviations: CAR: Central African Republic. CI: confidence interval. IPTp: intermittent preventive treatment pregnant women. PP: postpartum women. PW: pregnant women. Prim: primary school education. Sec: Secondary school education. The grey boxes indicate the weight for each study.
*Studies using cluster design. The studies by Marchant 2008, Gikandi 2008, Gies 2009, de Allegri 2013 and Hill 2013 took the study design into account in the analysis [2,5-7,9]. For Faye 2011 this is not clear [8].
§ Studies where an adjusted OR was used
Summary estimate: p <0.001. I^2 =33%, 95% CI 0-62%. Total population: 11,289 women in 17 studies (1 study, Antwi 2010, included two study populations).
Notes: The study population of Gies 2009 included only primi- and secundigravidae; the study population of Sangare 2010 included postpartum women who had at least 2 ANC visits which qualified for IPTp [1,6]. De study population of De Allegri 2013 and Amoran 2012 included women who had visited the ANC at least once [9,13].

***Table S2.2*: IPTp and education: sub-group analyses**

						Within subgroups		Between subgroups
**Covariate**	**Subgroup**	**N studies**	**Odds Ratio**	**Lower limit 95% CI**	**Upper limit 95% CI**	**p-value†**	***I^2***	**p-value†**
Number of SP doses	1+	7	1.73	1.31	2.27	0.320	14.4%	0.754
	2+	11	1.64	1.37	1.96	0.057	44.1%	
Location enrolment	Clinic	7	2.01	1.55	2.62	0.798	0.0%	0.077
	Community	11	1.53	1.31	1.78	0.063	43.1%	
Study population	Pregnant women	6	2.15	1.61	2.87	0.800	0.0%	**0.026**
	Post partum women	8	1.67	1.41	1.98	0.062	47.9%	
	Pregnant and postpartum	4	1.34	1.09	1.64	0.811	0.0%	
Country	Burkina Faso	2	1.41	0.77	2.59	0.924	0.0%	0.846
	Central African Republic	1	2.23	1.06	4.68			
	Ghana	3	1.51	0.90	2.53	0.445	0.0%	
	Kenya	4	1.74	1.23	2.48	0.004	77.1%	
	Nigeria	2	1.91	1.02	3.56	0.386	0.0%	
	Senegal	1	1.55	0.87	2.74			
	South Sudan	1	1.65	0.80	3.41			
	Tanzania	1	1.27	0.69	2.33			
	Uganda	3	2.61	1.42	4.80	0.976	0.0%	
Definition of education	Any vs. none	12	1.50	1.29	1.75	0.699	0.0%	0.049
	Other (Secondary+ vs. none/primary, literate, 8+ yrs educ.)	6	2.00	1.57	2.54	0.035	58.3%	
Study quality	Good	10	1.87	1.51	2.31	0.059	45.1	0.130
	Moderate-to-low	8	1.50	1.23	1.81	0.493	0.0%	

Abbreviations: CI: confidence interval. IPTp: Intermittent preventive treatment. SP: Sulfadoxine-pyrimethamine.
†p-value Q-statistic (assessment of heterogeneity)

Figure S2.3: **Location of residence and IPTp: Rural vs. urban**

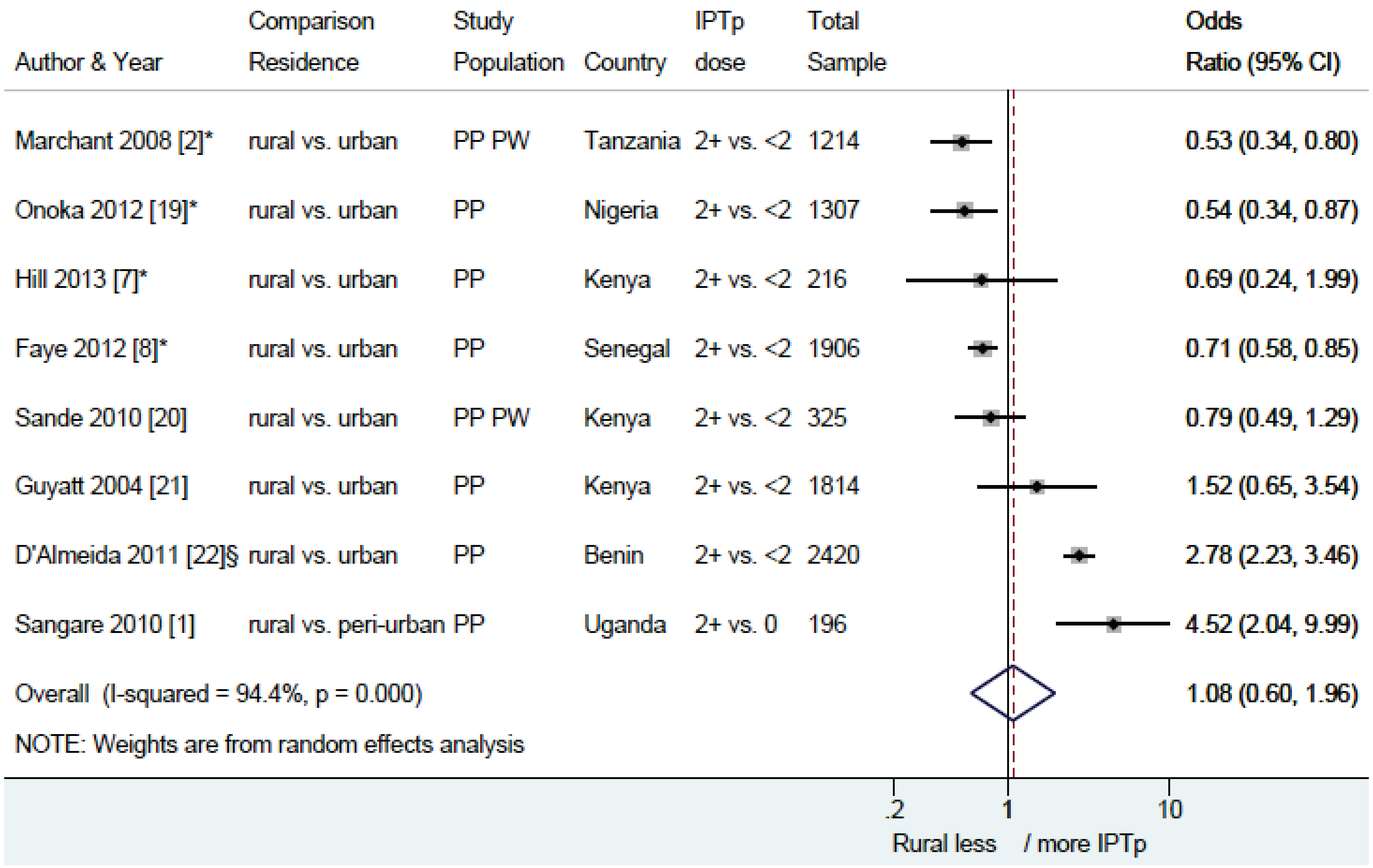

Abbreviations: CI: confidence interval. IPTp: intermittent preventive treatment pregnant women. PP: postpartum women; PW: pregnant women
The grey boxes indicate the weight for each study.
*Studies using cluster design. The studies by Marchant 2008 and Onoka 2012 took the study design into account in the analysis [2,19]. For Faye 2011 this is not clear [8].
§ Studies where an adjusted OR was used
Summary estimate: p =0.792. I^2 =94%, 95% CI 91-96%. Total population: 9398 women in 8 studies
Notes: The study population of Sangare 2010 included postpartum women who had at least 2 ANC visits which qualified for IPTp [1].

***Table S2.3*: IPTp and location residence sub-group analyses: Rural vs. urban**

						Within subgroups		Between subgroups
**Covariate**	**Subgroup**	**N studies**	**Odds Ratio**	**Lower limit 95% CI**	**Upper limit 95% CI**	**p-value†**	I^2	**p-value†**
Number of SP doses	All studies used 2+ doses							
Location enrolment	Clinic	2	1.54	0.63	3.76	<0.001	95.3%	0.355
	Community	6	0.94	0.53	1.64	<0.001	81.5%	
Study population	Postpartum women	6	1.31	0.64	2.66	<0.001	95.4%	0.315
	Pregnant and postpartum	2	0.64	0.20	2.11	0.216	34.7%	
Country	Benin	1	2.78	2.23	3.46			**<0.001**
	Kenya	3	0.89	0.61	1.32	0.369	0.0%	
	Nigeria	1	0.54	0.34	0.87			
	Senegal	1	0.71	0.58	0.85			
	Tanzania	1	0.53	0.34	0.80			
	Uganda	1	4.52	2.04	9.99			
Study quality	Good	6	1.09	0.53	2.24	<0.001	95.9%	0.982
	Moderate-to-low	2	1.07	0.30	3.81	0.187	42.5%	

Abbreviations: CI: confidence interval. IPTp: Intermittent preventive treatment pregnancy. SP: Sulfadoxine-pyrimethamine.
†p-value Q-statistic (assessment of heterogeneity)

***Figure S2.4*: Employment and IPTp**

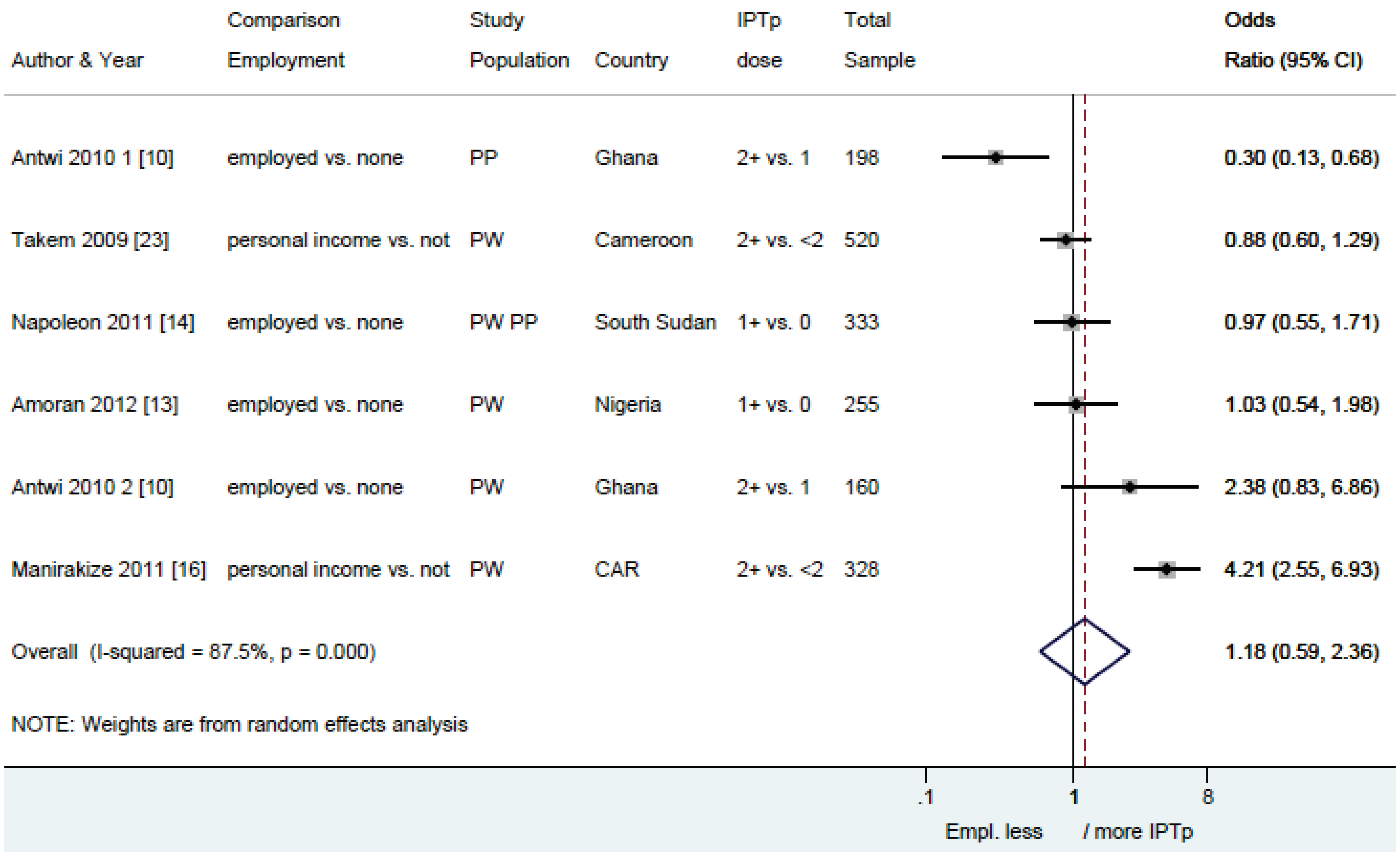

Abbreviations: CI: confidence interval. IPTp: intermittent preventive treatment pregnant women. PP: postpartum women. PW: pregnant women. Empl: Employed
The grey boxes indicate the weight for each study.
Summary estimate: p =0.644. I^2 =87%, 95% CI 75-94%. Total population: 1794 women in 6 studies
Notes: De study population of Amoran 2012 included women who had visited the ANC at least once [13].

***Table S2.4*: Employment and IPTp sub-group analyses: employed or salaried income vs. not**

						Within subgroups		Between subgroups
**Covariate**	**Subgroup**	**N studies**	**Odds Ratio**	**Lower limit 95% CI**	**Upper limit 95% CI**	**p-value†**	I^2	**p-value†**
Number of SP doses	1+	2	1.00	0.26	3.79	0.884	0.0%	0.769
	2+	4	1.28	0.49	3.35	<0.001	92.2%	
Location enrolment	All recruited from clinic							
Study population	Postpartum women	1	0.30	0.06	1.47			0.066
	Pregnant women	5	1.51	0.77	2.97	<0.001	85.6%	
Country	Cameroon	1	0.88	0.06	13.81			0.909
	Central African Republic	1	4.21	0.26	67.23			
	Ghana	2	0.82	0.11	6.29	0.002	89.1%	
	Nigeria	1	1.03	0.06	17.04			
	South Sudan	1	0.97	0.06	15.70			
Definition employment	Personal income	2	1.90	0.55	6.53	<0.001	95.8%	0.342
	Employed	4	0.90	0.35	2.28	0.016	71.0%	
Study quality	Good	2	0.79	0.21	2.90	0.002	89.1%	0.471
	Moderate-to-low	4	1.39	0.60	3.21	<0.001	88.8%	

Abbreviations: CI: Confidence interval. IPTp: Intermittent preventive treatment. SP: Sulfadoxine-pyrimethamine.
†p-value Q-statistic (assessment of heterogeneity)

Figure S2.5: **Marital status and IPTp: Married vs. not married**

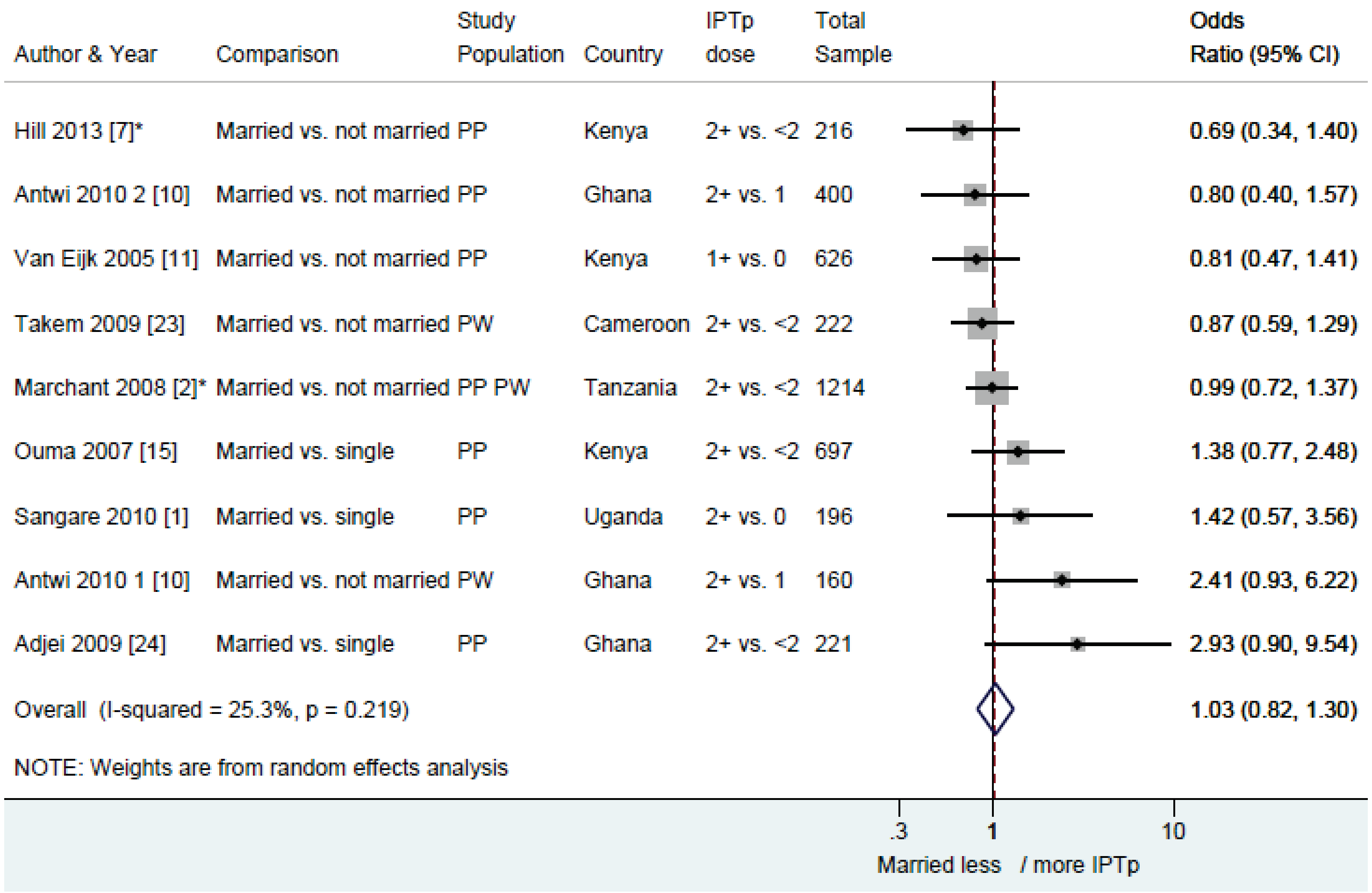

Abbreviations: CI: confidence interval. IPTp: intermittent preventive treatment pregnant women. PP: postpartum women. PW: pregnant women.
*Study using cluster design. Both Hill 2013 and Marchant 2008 took the study design into account in the analysis [2,7].
The grey boxes indicate the weight for each study.
Summary estimate: p =0.786. I^2 =25%, 95% CI 0-65%. Total population: 3952 women in 8 studies
Notes: The study population of Sangare 2010 included postpartum women who had at least 2 ANC visits which qualified for IPTp [1].

***Table S2.5*: Marital status and IPTp sub-group analyses: Married vs. not married**

						Within subgroups		Between subgroups
**Covariate**	**Subgroup**	**N studies**	**Odds Ratio**	**Lower limit 95% CI**	**Upper limit 95% CI**	**p-value†**	I^2	**p-value†**
Number of SP doses	1+	1	0.81	0.41	1.60			0.443
	2+	8	1.08	0.83	1.40	0.186	30.4%	
Location enrolment	Clinic	4	1.13	0.75	1.71	0.062	59.2%	0.627
	Community	5	1.00	0.72	1.38	0.506	0.0%	
Study population	Pregnant women	2	1.18	0.63	2.21	0.052	73.5%	0.930
	Post partum women	6	1.06	0.71	1.57	0.226	27.8%	
	Pregnant and postpartum	1	0.99	0.49	2.02			
Country	Cameroon	1	0.87	0.38	1.99			0.765
	Ghana	3	1.53	0.78	2.98	0.067	63.0%	
	Kenya	3	0.93	0.54	1.61	0.267	24.3%	
	Tanzania	1	0.99	0.45	2.19			
	Uganda	1	1.42	0.44	4.58			
Study quality	Good	7	1.14	0.86	1.50	0.170	33.8%	0.244
	Moderate-to-low	2	0.85	0.56	1.27	0.842	0.0%	

Abbreviations: CI: confidence interval. IPTp: Intermittent preventive treatment pregnancy. SP: Sulfadoxine-pyrimethamine.
†p-value Q-statistic (assessment of heterogeneity)

***Figure S2.6*: Age and IPTp: Older vs. younger age groups**

Author & Year	Comparison (yrs)	Study Population	Country	IPTp dose	Total sample	Odds Ratio (95% CI)
Manirakiza 2011 [16]	20+ vs. <20	PW	CAR	2+ vs. <2	328	0.64 (0.39, 1.04)
Hill 2013 [7]*	20+ vs. 15-19	PP	Kenya	2+ vs. <2	216	0.68 (0.31, 1.52)
Ouma 2007 [15]	20+ vs. <20	PP	Kenya	2+ vs. <2	698	0.72 (0.44, 1.18)
Bouyou-Akotet 2013 [25]	17+ vs. <17	PW	Gabon	2+ vs. <2	442	0.78 (0.30, 2.02)
Nganda 2005 [26]	20-29 vs. 15-19	PP	Tanzania	1+ vs. 0	234	0.80 (0.40, 1.59)
Napoleon 2011 [14]	18+ vs. <18	PP PW	South Sudan	1+ vs. 0	330	0.81 (0.43, 1.54)
Van Eijk 2005 [11]	20+ vs. <20	PP	Kenya	1+ vs. 0	627	0.86 (0.53, 1.42)
Adjei 2009 [24]	21-25 vs. 15-20	PP	Ghana	2+ vs. <2	118	0.88 (0.28, 2.76)
Amoran 2012 [13]	25+ vs. <25	PW	Nigeria	1+ vs. 0	255	0.89 (0.50, 1.61)
Gikandi 2008 [5]*	30-49 vs. 15-19	PP PW	Kenya	2+ vs. <2	356	0.93 (0.46, 1.87)
De Allegri 2013 [9]*§	25-34 vs. <25	PP	Burkina Faso	1+ vs. 0	359	1.05 (0.70, 1.58)
Marchant 2008 [2]*	20+ vs. <20	PP PW	Tanzania	2+ vs. <2	1201	1.09 (0.75, 1.58)
Sangare 2010 [1]	19+ vs. <19	PP	Uganda	2+ vs. 0	196	1.11 (0.47, 2.63)
Namusoke 2012 [18]	21+ vs. <21	PW	Uganda	1+ vs. 0	204	1.16 (0.61, 2.22)
Takem 2009 [23]	25-29 vs. <20	PW	Cameroon	2+ vs. <2	215	1.27 (0.67, 2.38)
Gies 2009 [6]*§	20+ vs. < 20	PP	Burkina Faso	2+ vs. <2	1513	1.33 (1.03, 1.73)
Mbonye 2010 [3]	20+ vs. 14-19	PW	Uganda	1+ vs. 0	804	1.47 (0.91, 2.39)
Iliyasu 2012 [17]	20+ vs. <20	PW	Nigeria	1+ vs. none	239	7.33 (1.68, 31.99)
Overall (I-squared = 23.6%, p = 0.176)						1.01 (0.87, 1.18)

NOTE: Weights are from random effects analysis

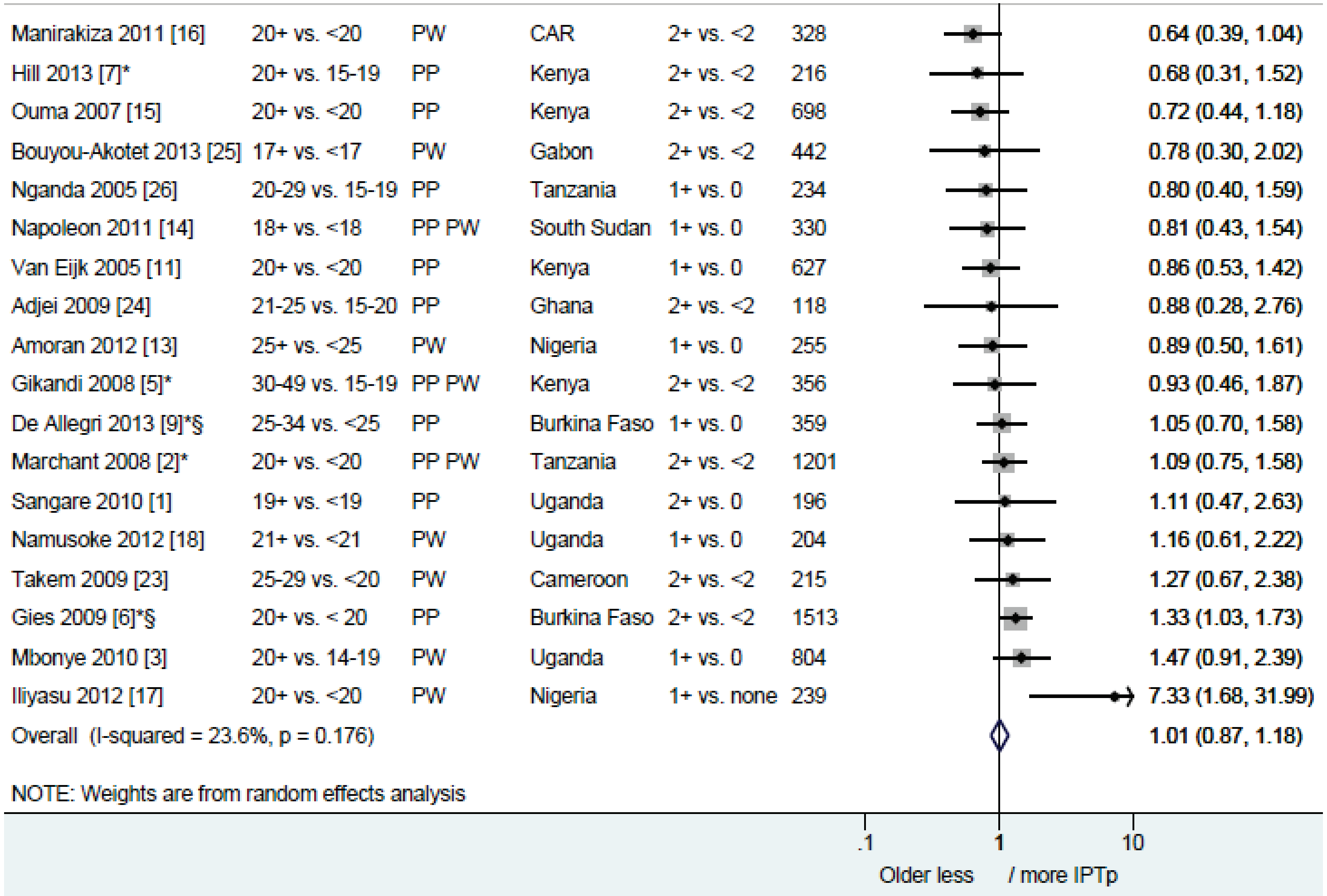

Abbreviations: CI: confidence interval. IPTp: intermittent preventive treatment pregnant women. PP: postpartum women. PW: pregnant women.
The grey boxes indicate the weight for each study.
*Studies using cluster design. The studies by Marchant 2008, Gikandi 2008, Gies 2009, de Allegri 2013 and Hill 2013 took the study design into account in the analysis [2,5-7,9].

§ Studies where an adjusted OR was used
Summary estimate: p =0.891. I^2 =24%, 95% CI 0-57%. Total population: 8335 women in 18 studies
Notes: The study population of Sangare 2010 included postpartum women who had at least 2 ANC visits which qualified for IPTp [1]. The study population of Gies 2009 included only primi- and secundigravidae [6]. De study population of De Allegri 2013 and Amoran 2012 included women who had visited the ANC at least once [9,13].

***Table S2.6*: Age and IPTp sub-group analyses: older age groups vs. younger age groups**

						Within subgroups		Between subgroups
**Covariate**	**Subgroup**	**N studies**	**Odds Ratio**	**Lower limit 95% CI**	**Upper limit 95% CI**	**p-value†**	**Within group I^2**	**p-value†**
Number of SP doses	1+	8	1.06	0.83	1.35	0.151	34.8%	0.584
	2+	10	0.97	0.79	1.20	0.245	21.5%	
Location enrolment	Clinic	9	0.93	0.72	1.20	0.164	31.7%	0.412
	Community	9	1.06	0.88	1.28	0.356	9.5%	
Study population	Pregnant women	7	1.08	0.81	1.44	0.034	56.0%	0.834
	Post partum women	8	0.97	0.76	1.23	0.346	10.8%	
	Pregnant and postpartum	3	0.98	0.66	1.42	0.730	0.0%	
Country	Burkina Faso	2	1.23	0.93	1.62	0.337	0.0%	0.346
	Cameroon	1	1.27	0.65	2.48			
	Central African Republic	1	0.64	0.37	1.09			
	Gabon	1	0.78	0.29	2.08			
	Ghana	1	0.88	0.27	2.83			
	Kenya	4	0.80	0.58	1.09	0.898	0.0%	
	Nigeria	2	1.23	0.69	2.20	0.009	85.2%	
	South Sudan	1	0.81	0.41	1.60			
	Tanzania	2	1.00	0.69	1.46	0.445	0.0%	
	Uganda	3	1.30	0.89	1.90	0.783	0.0%	
Study quality	Good	10	1.08	0.88	1.32	0.147	32.7%	0.347
	Moderate-to-low	8	0.93	0.74	1.17	0.420	1.2%	

Abbreviations: CI: Confidence interval. IPTp: Intermittent preventive treatment pregnancy. SP: Sulfadoxine-pyrimethamine.
†p-value Q-statistic (assessment of heterogeneity)

Figure S2.7**: Gravidity/Parity and IPTp: Low parity/gravidity number vs. higher**

Author & Year	Comparison	Study Population	Country	IPTp dose	Total Sample	Odds Ratio (95% CI)
Iliyasu 2012 [17]	Primi's vs. Multi's	PW	Nigeria	1+ vs. 0	239	0.27 (0.09, 0.79)
Gies 2009 [6]*	Primi's vs. Secundi's	PP	Burkina Faso	2+ vs. <2	1514	0.73 (0.61, 0.87)
Sangare 2010 [1]	Primi's vs. Multi's	PP	Uganda	2+ vs. 0	196	0.96 (0.49, 1.90)
Antwi 2010 1 [10]	Primi's vs. Multi's	PP	Ghana	2+ vs. 1	401	1.07 (0.53, 2.16)
Kiwuwa 2008 [27]	Primi's vs. Multi's	PP	Uganda	2+ vs. <2	768	1.16 (0.79, 1.70)
Ndyomugyenyi 2010 [28]	Primi's vs. Multi's	PP	Uganda	2+ vs. <2	418	1.35 (0.83, 2.18)
Amoran 2012 [13]	Primi's vs. Multi's	PW	Nigeria	1+ vs. 0	255	1.40 (0.78, 2.51)
Ouma 2007 [15]	Primi's vs. Multi's	PP	Kenya	2+ vs. <2	698	1.57 (0.97, 2.53)
Van Eijk 2005 [11]	Primi's vs. Multi's	PP	Kenya	1+ vs. 0	627	1.68 (1.04, 2.72)
Gikandi 2008 [5]*	Primi's vs. Multi's	PP PW	Kenya	2+ vs. <2	910	1.73 (1.17, 2.57)
Hill 2013 [7]*	Primi's vs. Multi's	PP	Kenya	2+ vs. <2	215	1.86 (0.87, 4.01)
Takem 2009 [23]	Primi's vs. Multi's	PW	Cameroon	2+ vs. <2	525	2.00 (1.29, 3.10)
Antwi 2010 2 [10]	Primi's vs. Multi's	PW	Ghana	2+ vs. 1	161	2.16 (0.60, 7.74)
Nduka 2011 [29]	Primi's vs. Multi's	PW	Nigeria	2+ vs. <2	844	3.83 (2.82, 5.19)
Manirakiza 2011 [16]	Primi's vs. Multi's	PW	CAR	2+ vs. <2	328	6.92 (4.11, 11.66)
Overall (I-squared = 90.4%, p = 0.000)						1.54 (1.05, 2.26)

NOTE: Weights are from random effects analysis

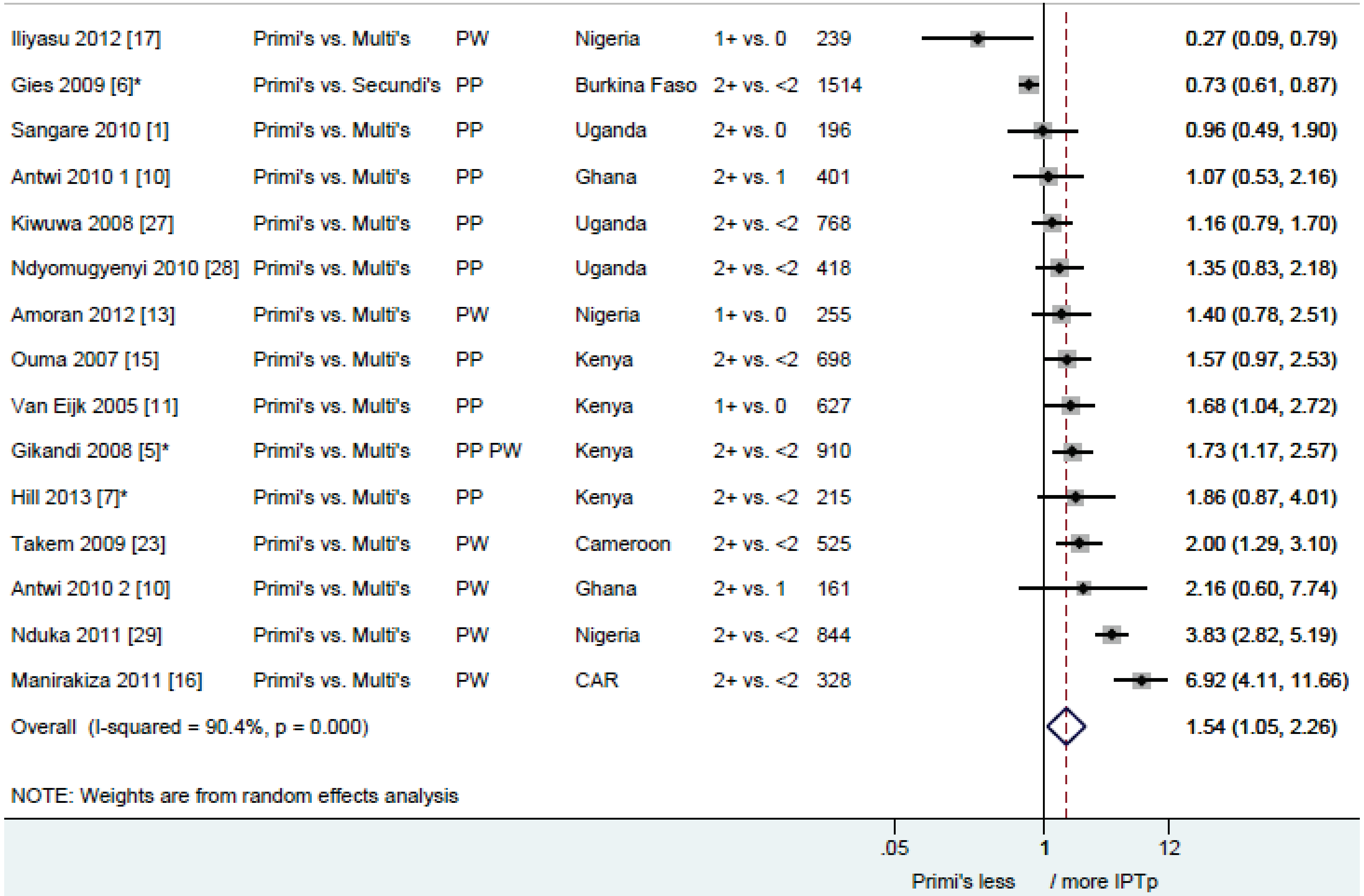

Abbreviations: CI: confidence interval. IPTp: intermittent preventive treatment pregnant women. Multi's: multigravidae (pregnant women) or multiparae (postpartum women). Primi's: primigravidae (pregnant women) or primiparae (postpartum women). PP: postpartum women. PW: pregnant women. Secundi's: secundigravidae (pregnant women or secundipara (postpartum women). The grey boxes indicate the weight for each study.

*Studies using cluster design. The studies by Gikandi 2008, Gies 2009, and Hill 2013 took the study design into account in the analysis [5-7].
§ Studies where an adjusted OR was used
Summary estimate: p =0.026. I^2 =90%, 95% CI 86-94%. Total population: 8099 women in 14 studies
Notes: The study population of Sangare 2010 included postpartum women who had at least 2 ANC visits which qualified for IPTp [1]. The study population of Gies 2009 included only primi- and secundiparae [6]. De study population of Amoran 2012 included women who had visited the ANC at least once [13].

***Table S2.7*: IPTp and gravidity/parity sub-group analyses: Low parity/gravidity number vs. higher**

						**Within subgroups**		**Between subgroups**
**Covariate**	**Subgroup**	**N studies**	**Odds Ratio**	**Lower limit 95% CI**	**Upper limit 95% CI**	**p-value†**	I^2	**p-value†**
Number of SP doses	1+	3	0.97	0.39	2.41	0.010	78.3%	0.269
	2+	12	1.71	1.11	2.65	<0.001	92.0%	
Location enrolment	Clinic	7	1.99	1.24	3.18	<0.001	87.6%	0.180
	Community	8	1.30	0.87	1.95	<0.001	78.1%	
Study population	Pregnant women	6	2.22	1.37	3.61	<0.001	87.8%	0.172
	Post partum women	8	1.22	0.83	1.81	0.001	71.0%	
	Pregnant and postpartum	1	1.73	0.60	5.00			
Country	Burkina Faso	1	0.73	0.30	1.79			0.055
	Cameroon	1	2.00	0.75	5.34			
	CAR	1	6.92	2.49	19.24			
	Ghana	2	1.37	0.55	3.39	0.349	0.0%	
	Kenya	4	1.70	1.02	2.83	0.981	0.0%	
	Nigeria	3	1.59	0.85	2.97	<0.001	92.6%	
	Uganda	3	1.16	0.64	2.09	0.724	0.0%	
Study quality	Good	8	1.05	0.71	1.57	0.002	68.7%	**0.006**
	Moderate-to-low	7	2.27	1.55	3.32	<0.001	84.7%	

Abbreviations: CI: confidence interval. IPTp: Intermittent preventive treatment pregnancy. SP:Sulfadoxine-pyrimethamine.
†P-value Q-statistic (assessment of heterogeneity)

***Figure S2.8*: Knowledge of malaria and IPTp: high vs. lower for malaria score**

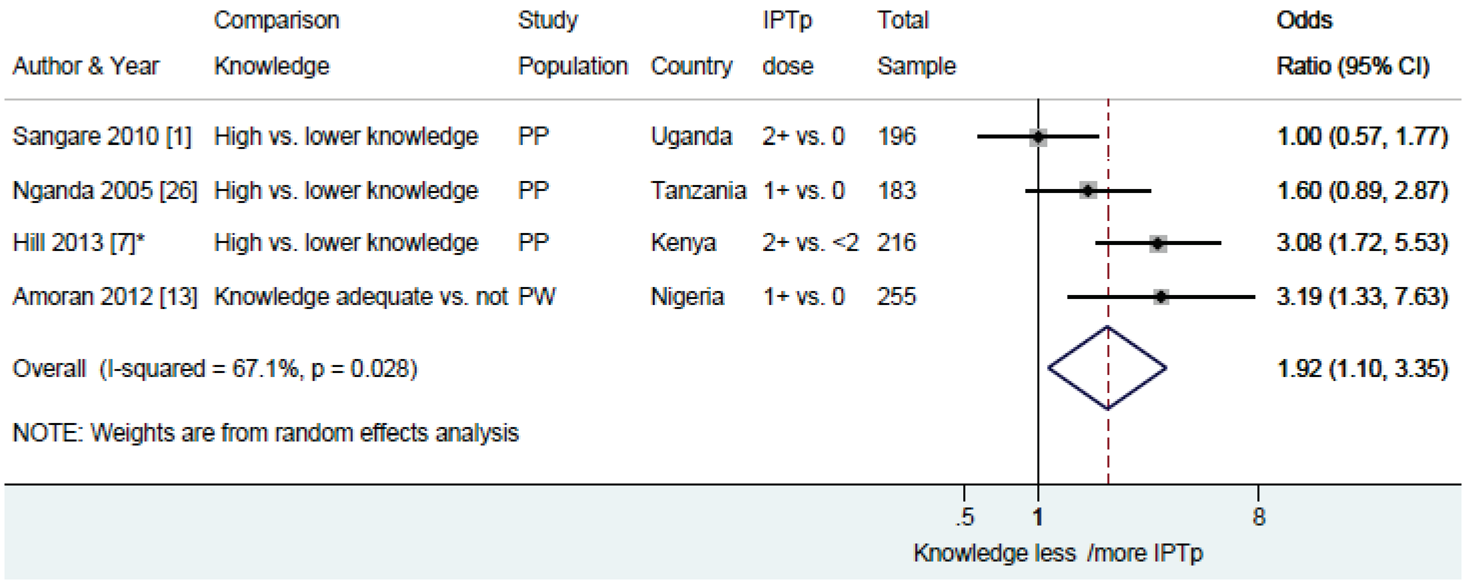

Abbreviations: CI: confidence interval. IPTp: Intermittent preventive treatment pregnant women. PP: postpartum women.
The grey boxes indicate the weight for each study.
*Studies using cluster design. The studies by Hill 2013 took the study design into account in the analysis [7].
Summary estimate: p =0.021. I^2 =67%, 95% CI 4%-89%. Total population: 850 women in 4 studies
Notes: The study population of Sangare 2010 included postpartum women who had at least 2 ANC visits which qualified for IPTp [1]. De study population of Amoran 2012 included women who had visited the ANC at least once [13].

***Table S2.8*: IPTp and knowledge of malaria sub-group analyses: High knowledge vs. lower knowledge**

						Within subgroups		Between subgroups
**Covariate**	**Subgroup**	**N studies**	**Odds Ratio**	**Lower limit 95% CI**	**Upper limit 95% CI**	**p-value†**	I^2	**p-value†**
Number of SP doses	1+	2	2.18	0.81	5.87	0.198	39.5%	0.756
	2+	2	1.75	0.69	4.49	0.007	86.3%	
Location enrolment	Clinic/community		Same division as above for SP dose					0.756
Study population	Pregnant women	1	3.19	0.88	11.54			0.391
	Post partum women	3	1.70	0.90	3.22	0.026	72.7%	
Country			All studies in different countries, no subgroup analysis conducted by country					
Study quality	Good	2	1.75	0.69	4.49	0.007	86.3%	0.756
	Moderate-to-low	2	2.18	0.81	5.87	0.198	39.5%	Same division as SP dose

Abbreviations: CI: confidence interval. IPTp: Intermittent preventive treatment pregnancy. SP:Sulfadoxine-pyrimethamine. †P-value Q-statistic (assessment of heterogeneity)

***Figure S2.9*: Distance to ANC and IPTp sub-group analyses: Farther away vs. nearby**

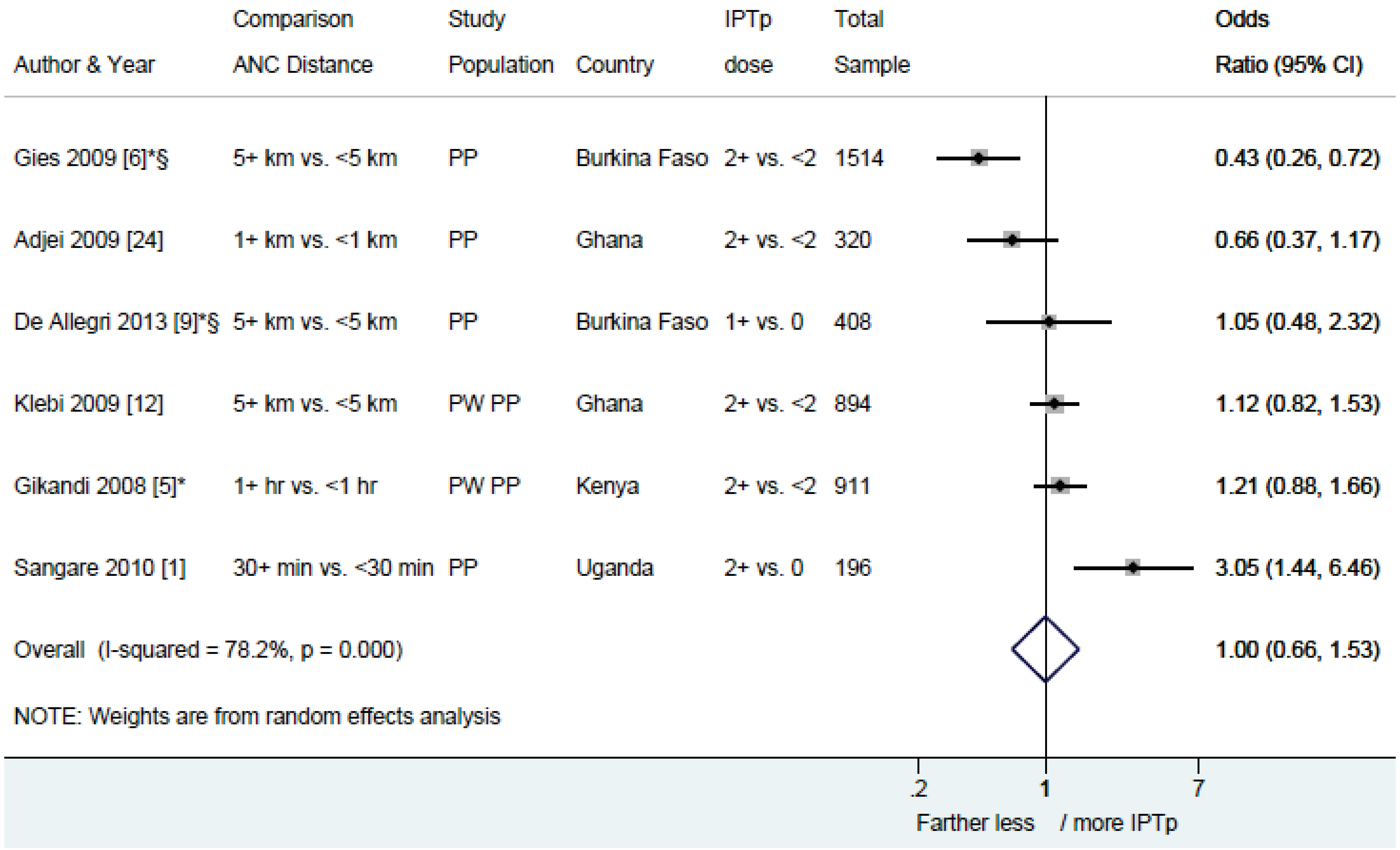

Abbreviations: ANC: antenatal clinic. CI: confidence interval. GE: greater or equal. Hr: hours. IPTp: intermittent preventive treatment pregnant women. LE: less or equal. Min: minutes. Km: kilometres. PP: postpartum women. PW: pregnant women. The grey boxes indicate the weight for each study.
*Studies using cluster design. The studies by Gikandi 2008, Gies 2009, and de Allegri 2013 took the study design into account in the analysis [5,6,9].
§ Studies where an adjusted OR was used
Summary estimate: p =0.984. I^2 =78%, 95% CI 52-90%. Total population: 4243 women in 6 studies.
Notes: The study population of Sangare 2010 included postpartum women who had at least 2 ANC visits which qualified for IPTp [1]. The study population of Gies 2009 included only primi- and secundigravidae [6]. De study population of De Allegri 2013 included women who had visited the ANC at least once [9].

***Table S2.9*: Distance to ANC and IPTp sub-group analyses: Farther away vs. nearby**

						Within subgroups		Between subgroups
**Covariate**	**Subgroup**	**N studies**	**Odds ratio**	**Lower limit 95% CI**	**Upper limit 95% CI**	**p-value†**	I^2	**p-value†**
Number of SP doses	1+	1	1.05	0.31	3.61			0.939
	2+	5	1.00	0.62	1.61	<0.001	82.5%	
Location enrolment	Clinic	1	0.66	0.22	1.95			0.405
	Community	5	1.09	0.68	1.76	<0.001	80.4%	
Study population	Postpartum women	4	0.91	0.52	1.60	<0.001	84.0%	0.590
	Pregnant and postpartum	2	1.16	0.59	2.32	0.742	0.0%	
Country	Burkina Faso	2	0.62	0.31	1.25	0.062	71.4%	0.099
	Ghana	2	0.89	0.48	1.66	0.109	61.1%	
	Kenya	1	1.21	0.53	2.76			
	Uganda	1	3.05	1.05	8.88			
Study quality	Good	4	0.91	0.52	1.60	<0.001	84.0%	0.590
	Moderate-to-low	2	1.16	0.59	2.32	0.742	0.0%	

Abbreviations: CI: confidence interval. IPTp: intermittent preventive treatment pregnancy. SP: Sulfadoxine-pyrimethamine.
†P-value Q-statistic (assessment of heterogeneity)

Figure S2.10**: Timing of start ANC visits and IPTp: start in 1st or 2nd trimester vs. 3rd trimester**

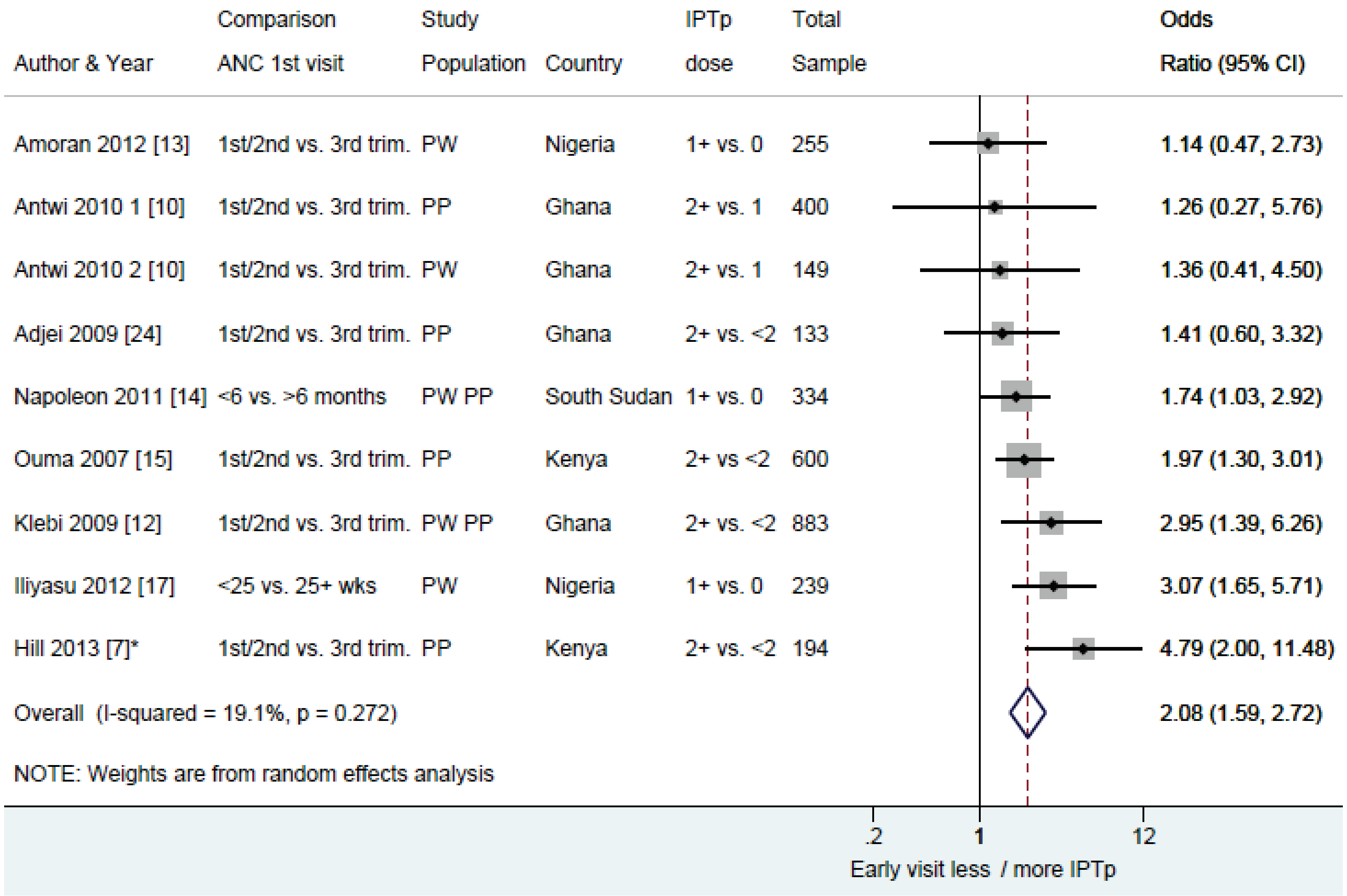

Abbreviations: ANC: Antenatal clinic. CI: confidence interval. IPTp: intermittent preventive treatment pregnant women. M: months of gestational age. PP: postpartum women. PW: pregnant women. Trim: trimester. The grey boxes indicate the weight for each study.
*Studies using cluster design. The study by Hill 2013 took the study design into account in the analysis [7].
Summary estimate: p <0.001. I^2 =19%, 95% CI 0-61%. Total population: 3187 in 8 studies
Notes: De study population of Amoran 2012 included women who had visited the ANC at least once [13].

***Table S2.10*: Timing of start ANC visits and IPTp sub-group analyses: earlier vs. later start ANC visits**

						Within subgroups		Between subgroups
**Covariate**	**Subgroup**	**N studies**	**Odds Ratio**	**Lower limit 95% CI**	**Upper limit 95% CI**	**p-value†**	I^2	**p-value†**
Number of SP doses	1+	3	1.94	1.22	3.08	0.157	46.0%	0.708
	2+	6	2.17	1.48	3.19	0.302	17.3%	
Location enrolment	Clinic	6	1.77	1.25	2.51	0.459	0.0%	0.172
	Community	3	2.56	1.72	3.81	0.173	43.1%	
Study population	Postpartum women	4	2.14	1.28	3.56	0.188	37.3%	0.946
	Pregnant women	3	1.89	1.03	3.49	0.148	47.6%	
	Pregnant and postpartum	2	2.16	1.15	4.05	0.26	22.4%	
Country	Ghana	4	1.79	0.95	3.37	0.501	0.0%	0.816
	Kenya	2	2.72	1.36	5.42	0.073	68.9%	
	Nigeria	2	2.03	0.96	4.29	0.070	69.6%	
	South Sudan	1	1.74	0.70	4.34			
Study quality	Good	6	2.23	1.56	3.20	0.252	24.2%	0.522
	Moderate-to-low	3	1.84	1.15	2.94	0.257	26.3%	

Abbreviations: CI: confidence interval; IPTp: Intermittent preventive treatment pregnancy. SP: sulfadoxine-pyrimethamine.
†P-value Q-statistic (assessment of heterogeneity)

***Figure S2.11*: Number of ANC visits and IPTp: 3-4 ANC visits vs. <3-4 visits**

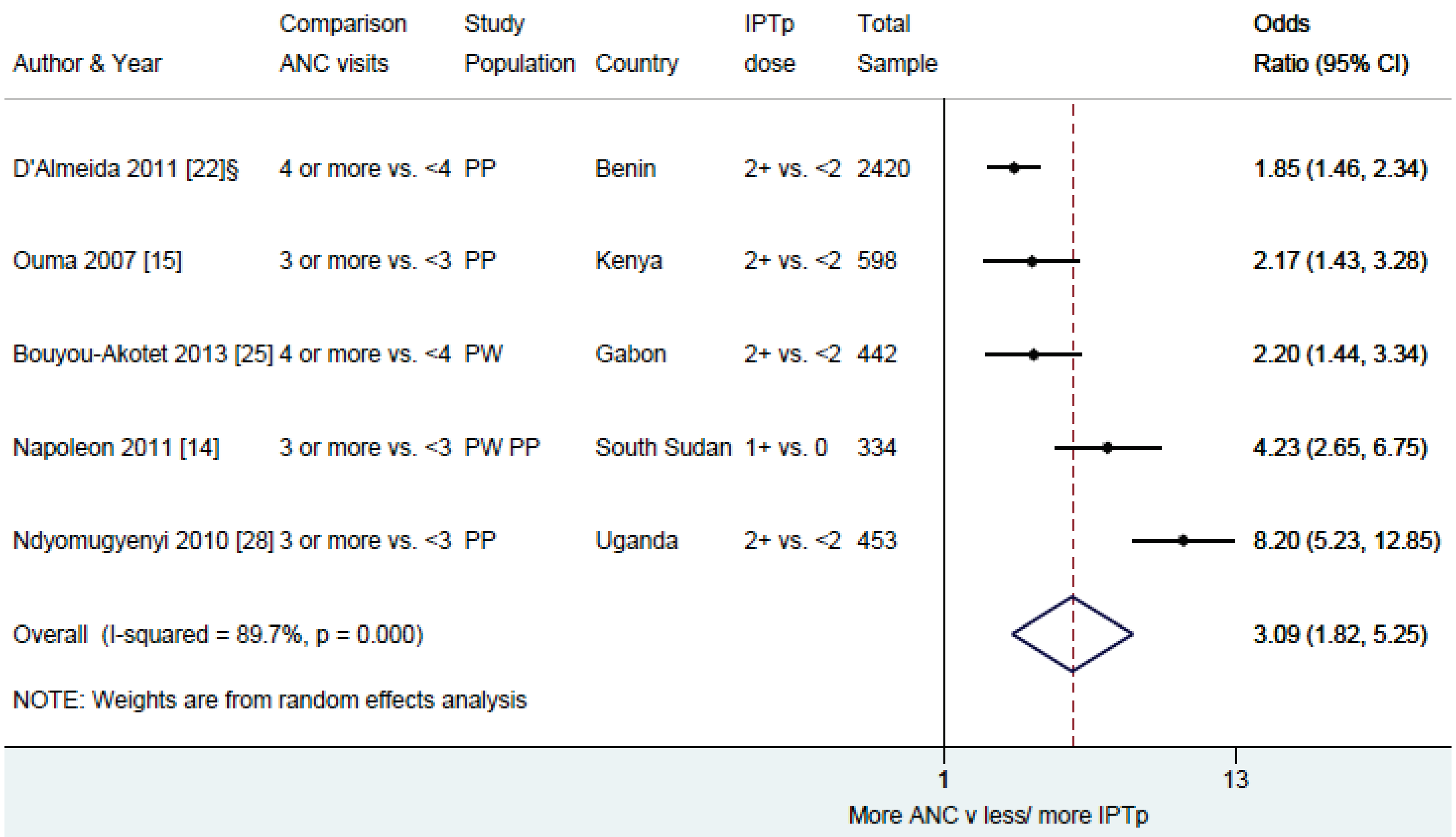

Abbreviations: ANC: antenatal clinic. CI: confidence interval. IPTp: intermittent preventive treatment pregnant women. PP: postpartum women. PW: pregnant women. V: visit.
The grey boxes indicate the weight for each study.
* Studies using cluster design
§ Studies where an adjusted OR was used
Summary estimate: p <0.001. I^2 =90%, 95% CI 79-95%. Total population: 4219 in 5 studies

***Table S2.11*: Number of ANC visits and IPTp sub-group analyses: 3-4 ANC visits vs. <3-4 visits**

						Within subgroups		Between subgroups
**Covariate**	**Subgroup**	**N studies**	**Odds Ratio**	**Lower limit 95% CI**	**Upper limit 95% CI**	**p-value†**	I^2	**p-value†**
Number of SP doses	1+	2	2.74	1.07	7.03	0.002	89.6%	0.739
	2+	3	3.38	1.55	7.36	<0.001	91.4%	
Location enrolment	Clinic	3	2.54	1.29	4.99	0.008	79.2%	0.364
	Community	2	4.19	1.81	9.68	<0.001	94.5%	
Study population	Postpartum women	3	3.16	1.35	7.40	<0.001	94.1%	0.830
	Pregnant women	1	2.20	0.50	9.71			
	Pregnant and postpartum	1	4.23	0.94	18.97			
Country	In 5 different countries							
Study quality	Good	3	2.01	1.56	2.61	0.692	0.0%	**<0.001**
	Moderate-to-low	2	5.94	4.05	8.71	0.045	75.0%	

Abbreviations: ANC: antenatal clinic. CI: confidence interval. IPTp: Intermittent preventive treatment pregnancy. SP: Sulfadoxine-pyrimethamine
†p-value Q-statistic (assessment of heterogeneity).

***Figure S2.12*: Other malaria prevention and IPTp: ITN vs. no ITN**

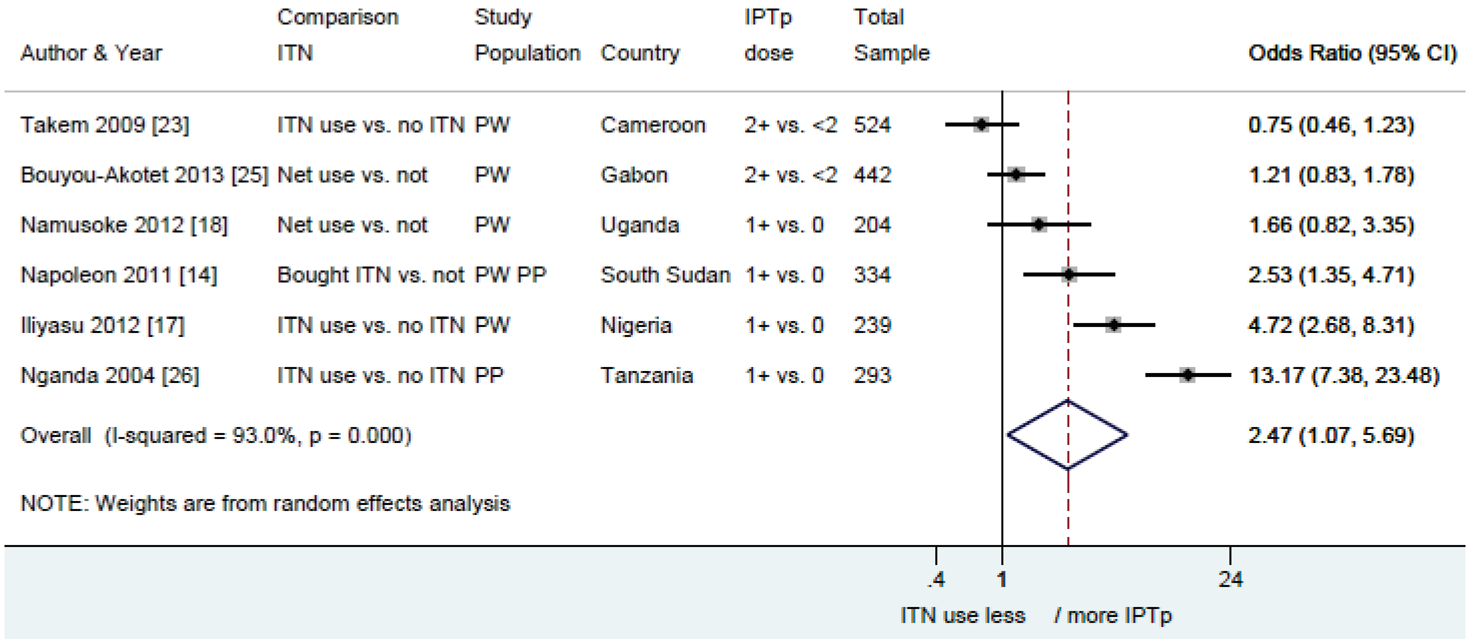

Abbreviations: CI: confidence interval. IPTp: intermittent preventive treatment pregnant women.ITN: insecticide treated net. PP: postpartum women. PW: pregnant women. The grey boxes indicate the weight for each study. Summary estimate: p =0.034. I^2 =93%, 95% CI 88-96%. Total population: 2036 women in 6 studies

***Table S2.12*: IPTp and interventions sub-group analyses: ITN vs. no ITN**

						**Within subgroups**		**Between subgroups**
**Covariate**	**Subgroup**	**N studies**	**Odds ratio**	**Lower limit 95% CI**	**Upper limit 95% CI**	**p-value†**	I^2	**p-value†**
Number of SP doses	1+	4	4.10	1.98	8.47	<0.001	87.7%	**0.020**
	2+	2	0.96	0.36	2.56	0.135	55.2%	
Location enrolment	All recruited from clinic							
Study population	Postpartum women	1	13.17	2.94	49.02			0.049
	Pregnant women	4	1.61	0.77	3.39	<0.001	87.7%	
	Pregnant and postpartum	1	2.53	0.55	11.52			
Country	In 6 different countries							
Study quality	Good	3	2.11	0.56	7.94	<0.001	87.0%	0.739
	Moderate-to-low	3	2.90	0.77	10.92	<0.001	96.3%	

Abbreviations: CI: confidence interval. IPTp: Intermittent preventive treatment pregnancy. ITN: insecticide treated net. SP: Sulfadoxine-pyrimethamine.
†p-value Q-statistic (assessment of heterogeneity)

Figure S2.13**: Socioeconomic status and ITN use: higher vs. lower socioeconomic status**

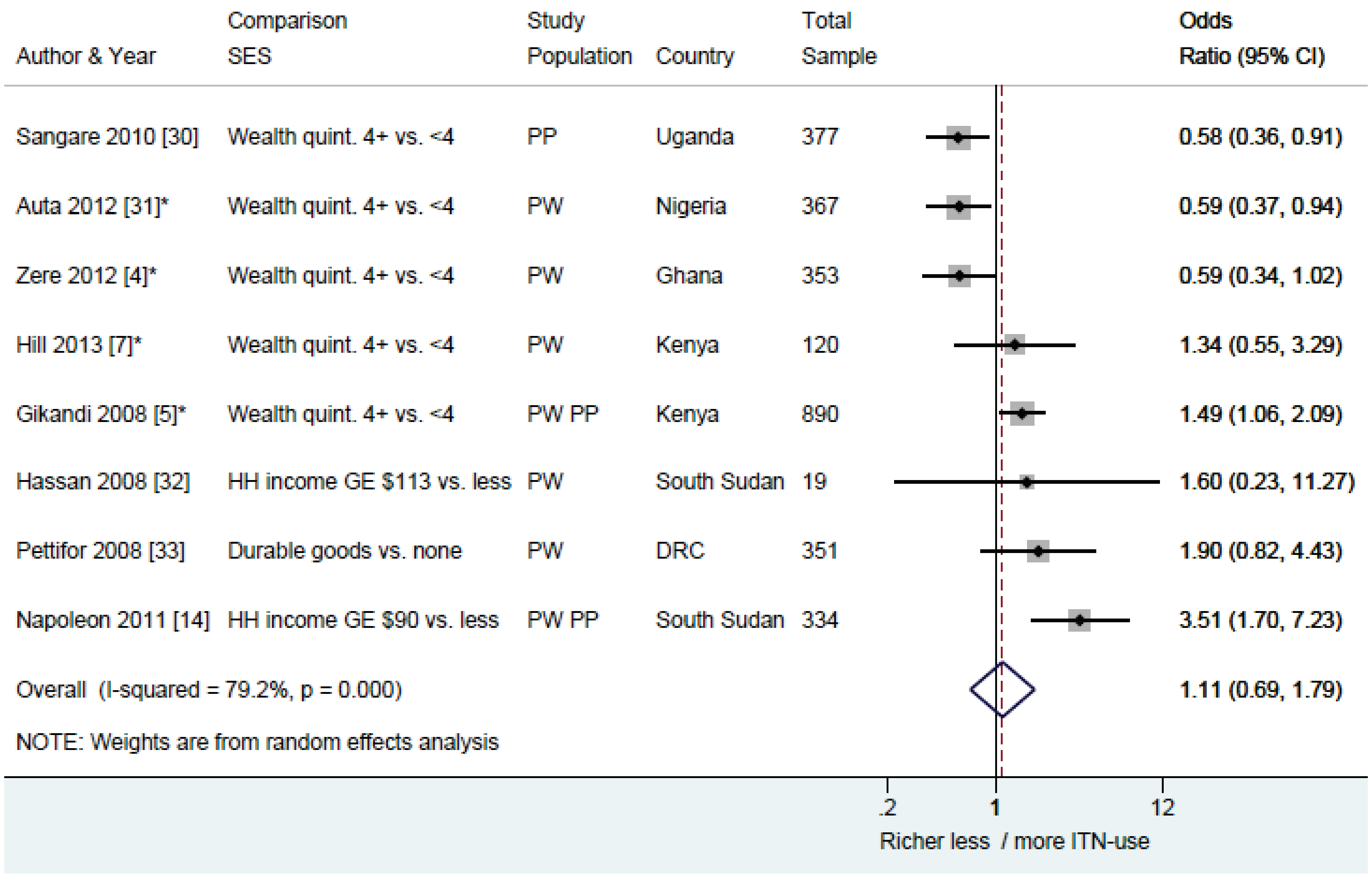

Abbreviations: CI: confidence interval. GE: greater or equal. HH: household. ITN: insecticide treated net. PP: postpartum women. PW: pregnant women. Quint: quintiles. SES: socioeconomic status. The grey boxes indicate the weight for each study.

*Study with a cluster design. The studies by Gikandi 2008, and Hill 2013 took the study design into account in the analysis [5,7]. For Auta 2012 and Zere 2012 this was not clear [4,31].

Summary estimate: p =0.657. I^2 =79%, 95% CI 59-89%. Total population: 2811 women in 8 studies

Notes: Sangare 2010: Net use vs. none net use among net owners [30]. Auta 2012: ITN use in households with ITNs [31].

***Table S2.13*: Socioeconomic status and ITN use sub-group analyses: Higher vs. lower socioeconomic status**

						Within subgroups		Between subgroups
**Covariate**	**Subgroup**	**N studies**	**Odds ratio**	**Lower limit 95% CI**	**Upper limit 95% CI**	**p-value†**	**Within group I^2**	**p-value†**
Type of net	ITN	6	1.18	0.65	2.14	<0.001	80.1%	0.735
	Net	2	0.97	0.36	2.60	0.015	83.1%	
Net use when	Last night	5	0.95	0.50	1.83	0.073	53.4%	0.465
	Pregnancy	3	1.38	0.66	2.88	<0.001	89.9%	
Location enrolment	Clinic	2	2.64	1.16	6.01	0.280	14.3%	**0.017**
	Community	6	0.84	0.54	1.31	0.002	73.2%	
Study population	Pregnant women	5	0.91	0.54	1.56	0.073	53.4%	0.071
	Post partum women	1	0.58	0.22	1.54			
	Post partum or pregnant	2	2.11	1.03	4.33	0.036	77.3%	
Country	Democratic Republic of Congo	1	1.90	0.82	4.43			**<0.001**
	Ghana	1	0.59	0.34	1.02			
	Kenya	2	1.47	1.07	2.02	0.826	0.0%	
	Nigeria	1	0.59	0.37	0.94			
	South Sudan	2	3.19	1.62	6.29	0.460	0.0%	
	Uganda	1	0.58	0.36	0.91			
Study Quality	Good	3	0.91	0.37	2.23	0.184	40.9%	0.589
	Moderate-to-low	5	1.23	0.68	2.23	<0.001	84.8%	

Abbreviations: CI: confidence interval. ITN: Insecticide treated net.
†p-value Q-statistic (assessment of heterogeneity)

Figure S2.14: **Education and ITN use: More vs. less education**

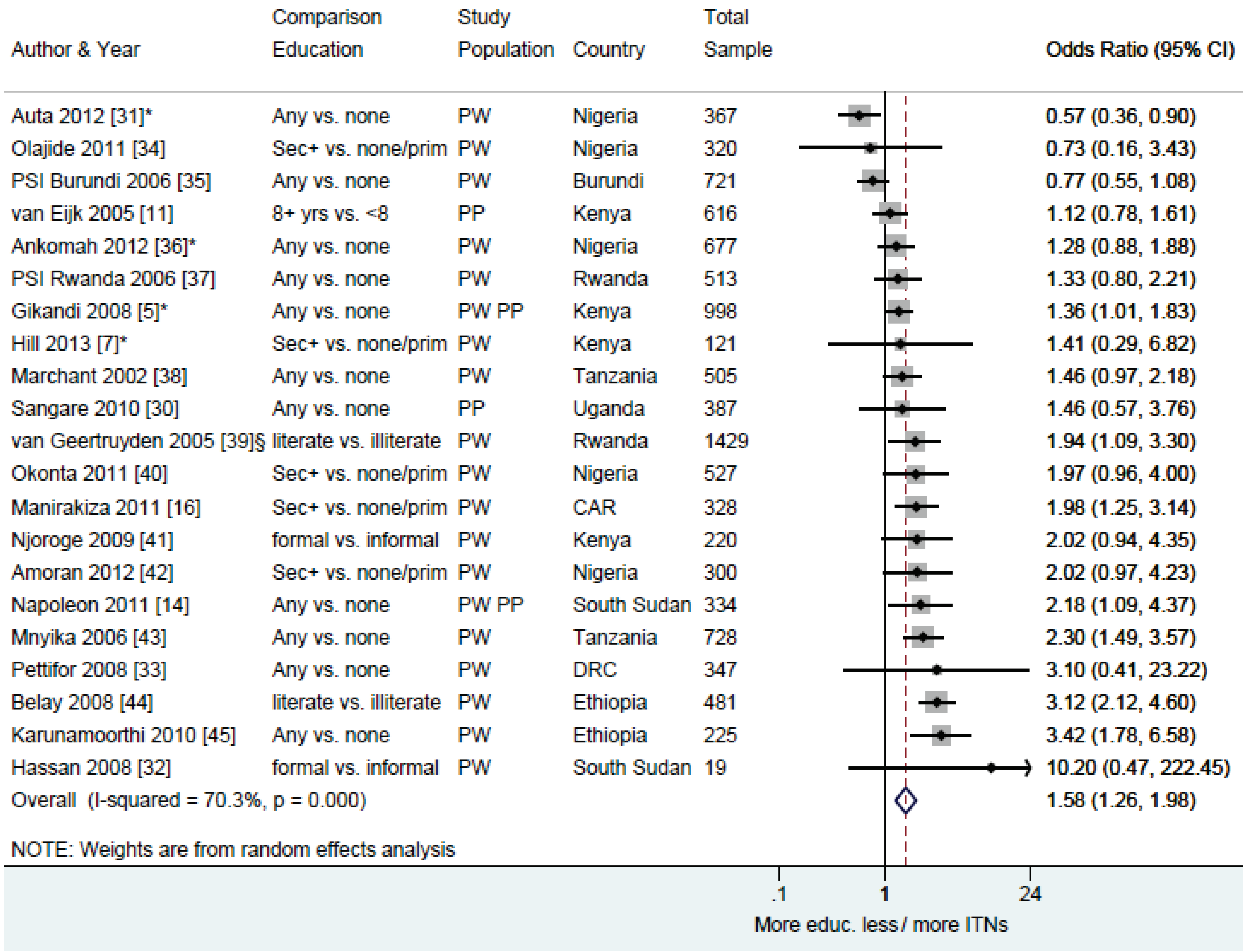

Abbreviations: CI: confidence interval. Educ: Education. ITN: insecticide treated net. PP: postpartum women. PW: pregnant women. Prim: primary school education. Sec: secondary school education. The grey boxes indicate the weight for each study.
*Studies using a cluster design. The studies by Gikandi 2008, and Hill 2013 took the study design into account in the analysis [5,7]. For Auta 2011 and Ankomah 2012 this was not clear [31,36].
§ Studies where an adjusted OR was used
Summary estimate: p <0.001. I^2 =70%, 95% CI 54-81%. Total population: 10,163 women in 21 studies.
Notes: Sangare 2010 net use vs. non-net use in households with nets [30]. Auta 2012 & Belay 2008: ITN use vs. none-use in households with ITNs [31,44].
PSI Rwanda 2006 and PSI Burundi 2006 may have used cluster design; this is not clear from the reports [35,37].

***Table S2.14*: Education and ITN use sub-group analyses: More vs. less education**

						**Within subgroups**		**Between subgroups**
**Covariate**	**Subgroup**	**N studies**	**Odds Ratio**	**Lower limit 95% CI**	**Upper limit 95% CI**	**p-value†**	**Within group I^2**	**p-value†**
Type of net	ITN	17	1.51	1.18	1.92	<0.001	73.8%	0.348
	Net	4	2.02	1.16	3.52	0.811	0.0%	
Net use when	Last night	8	1.27	0.87	1.85	<0.001	83.8%	0.157
	Pregnancy	13	1.78	1.35	2.34	0.159	28.4%	
Location enrolment	Clinic	10	2.13	1.56	2.89	0.884	0.0%	**0.011**
	Community	11	1.26	0.98	1.63	<0.001	77.2%	
Study population	Pregnant women	17	1.63	1.24	2.15	<0.001	74.8%	0.791
	Post partum women	2	1.23	0.57	2.67	0.611	0.0%	
	Post partum or pregnant	2	1.65	0.80	3.40	0.221	33.2%	
Country	Burundi	1	0.77	0.42	1.42			**0.028**
	Central African Republic	1	1.98	1.00	3.94			
	DRC	1	3.10	0.39	24.75			
	Ethiopia	2	3.23	1.94	5.36	0.813	0.0%	
	Kenya	4	1.36	0.93	1.98	0.576	0.0%	
	Nigeria	5	1.15	0.80	1.65	0.007	71.7%	
	Rwanda	2	1.59	0.95	2.68	0.323	0.0%	
	South Sudan	2	2.43	1.06	5.59	0.339	0.0%	
	Tanzania	2	1.82	1.14	2.90	0.131	56.2%	
	Uganda	1	1.46	0.50	4.28			
Definition of education	Any vs. none	10	1.52	1.10	2.10	0.001	69.7%	0.716
	Other (Secondary+ vs. none/primary, literate, 8+ yrs educ.)	11	1.66	1.18	2.32	<0.001	73.2%	
Study quality	Good	8	1.53	1.03	2.26	<0.001	77.4%	0.812
	Moderate-to-low	13	1.62	1.21	2.16	<0.001	66.7%	

Abbreviations: CI: confidence interval. DRC: Democratic Republic of Congo. ITN: insecticide treated nets.
† p-value Q-statistic (assessment of heterogeneity).

***Figure S2.15*: Location of residence and ITNs: Urban vs. rural**

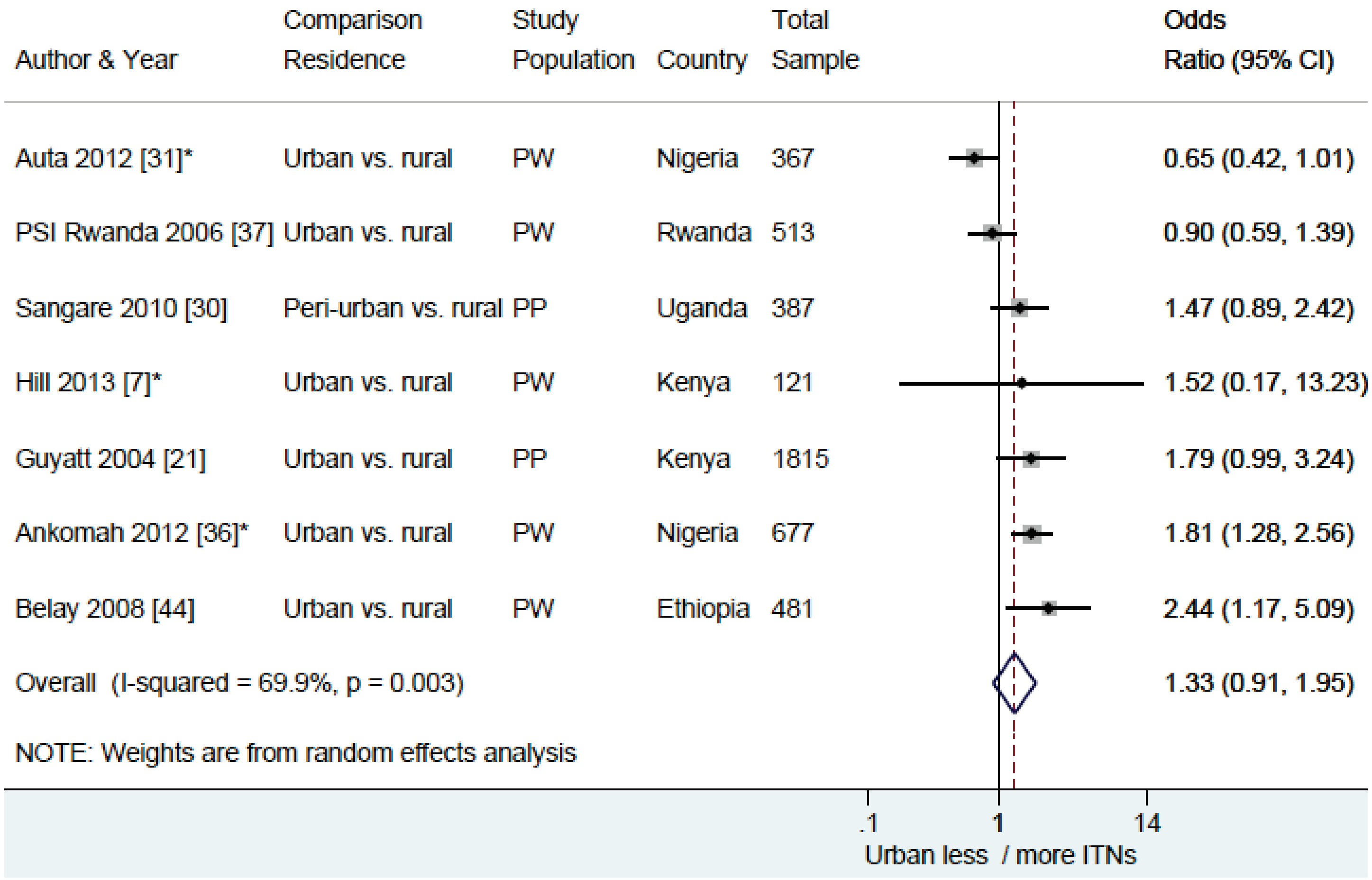

Abbreviations: CI: confidence interval. ITN: insecticide treated net. PP: postpartum women. PW: pregnant women.
The grey boxes indicate the weight for each study.
*Studies using a cluster design. The study by Hill 2013 took the study design into account in the analysis [7]. For Auta 2011 and Ankomah 2012 this was not clear [31,36].
Summary estimate: p =0.135. I^2 =70%, 95% CI 34-86%. Total population: 4360 women in 7 studies.
Notes: Notes: Sangare 2010 net use vs. non-net use in households with nets [30]. Auta 2012 & Belay 2008: ITN use vs. none-use in households with ITNs [31,44].
PSI Rwanda 2006 may have used a cluster design; this is not clear from the reports [37].

***Table S2.15*: Location of residence and ITN use sub-group analyses: Urban vs. rural**

						**Within subgroups**		**Between subgroups**
**Covariate**	**Subgroup**	**N studies**	**Odds Ratio**	**Lower limit 95% CI**	**Upper limit 95% CI**	**p-value†**	**Within group I^2**	**p-value†**
Type of net	ITN	6	1.32	0.84	2.08	0.001	74.6%	0.853
	Net	1	1.47	0.52	4.11			
Net use when	Last night	5	1.23	0.76	1.99	0.001	77.9%	0.542
	Pregnancy	2	1.61	0.79	3.31	0.615	0.0%	
Location enrolment	All enrolled in community							
Study population	Pregnant women	5	1.23	0.76	1.99	0.001	77.9%	0.542
	Post partum women	2	1.61	0.79	3.31	0.615	0.0%	(Same division as Net use)
Country	Ethiopia	1	2.44	0.57	10.49			0.853
	Kenya	2	1.72	0.51	5.81	0.885	0.0%	
	Nigeria	2	1.10	0.43	2.79	<0.001	92.2%	
	Rwanda	1	0.90	0.24	3.41			
	Uganda	1	1.47	0.38	5.68			
Study quality	Good	5	1.50	0.96	2.36	0.086	50.9%	0.352
	Moderate-to-low	2	1.03	0.54	1.98	0.007	86.1%	

Abbreviations: CI: confidence interval. ITN: insecticide treated net.
†p-value Q-statistic (assessment of heterogeneity)

Figure S2.16: **Employment status and ITN use: Employed vs. housewife or farmer**

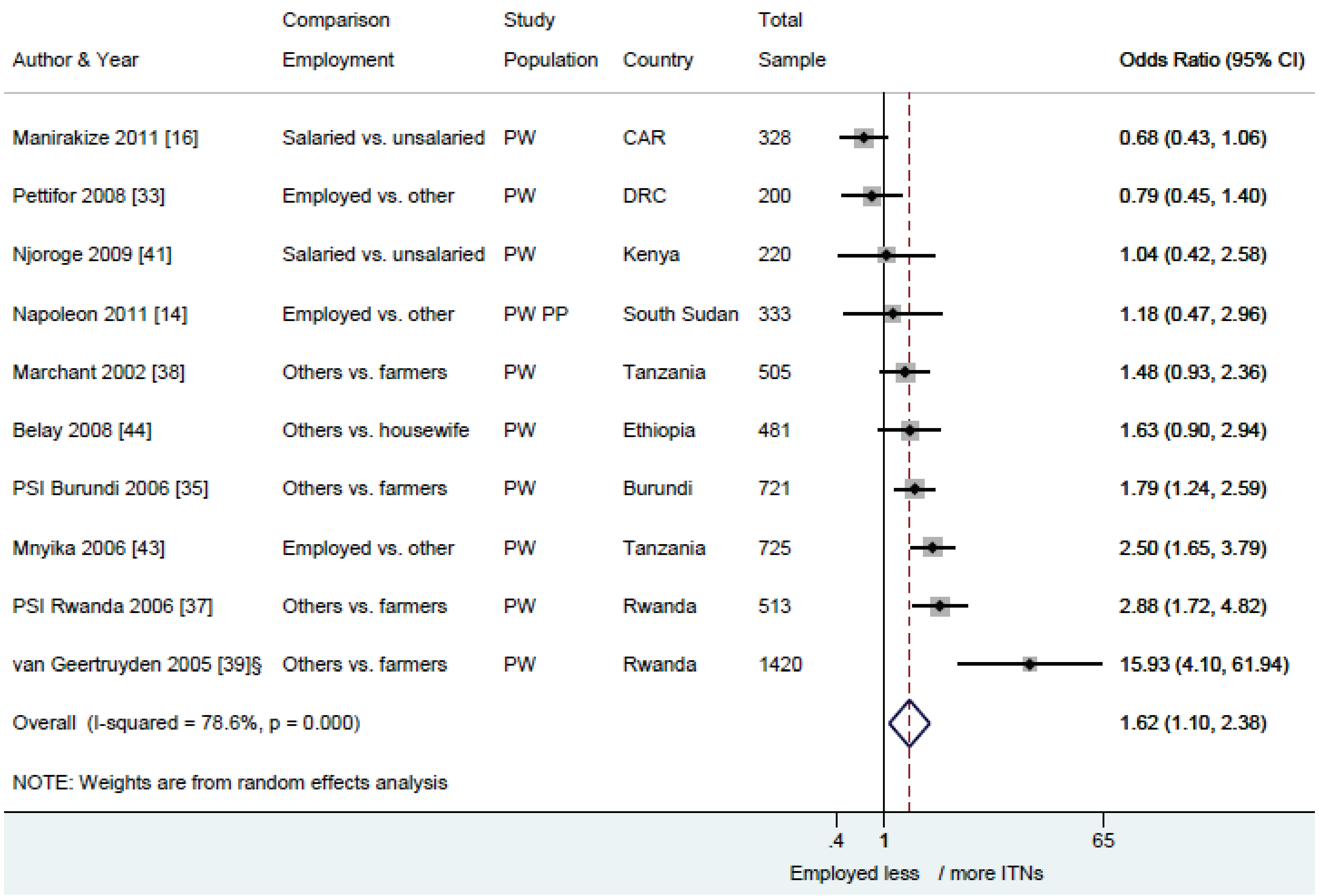

Abbreviations: CAR: Central African Republic. CI: confidence interval. DRC: Democratic Republic of Congo. ITN: insecticide treated net. PP: postpartum women. PW: pregnant women. The grey boxes indicate the weight for each study.

*Studies using cluster design. § Studies where an adjusted OR was used

Summary estimate: p =0.015. I^2 =79%, 95% CI 61-88%. Total population: 5446 women in 10 studies.

Notes: Belay 2008: ITN use vs. none ITN use among ITN owners [44].

PSI Rwanda 2006 and PSI Burundi 2006 may have used cluster design; this is not clear from the reports [35,37].

***Table S2.16*: Employment status and ITN use sub-group analyses: Employed vs. housewife or farmer or salaried vs. unsalaried**

						Within subgroups		Between subgroups
**Covariate**	**Subgroup**	**N studies**	**Odds Ratio**	**Lower limit 95% CI**	**Upper limit 95% CI**	**p-value†**	**Within group I^2**	**p-value†**
Type of net	ITN	7	1.42	0.88	2.29	0.003	69.8%	0.297
	Net	3	2.30	1.07	4.98	<0.001	90.1%	
Net use when	Last night	4	1.62	0.87	3.02	0.012	72.7%	0.983
	Pregnancy	6	1.63	0.94	2.84	<0.001	83.5%	
Location enrolment	Clinic	6	1.44	0.84	2.45	<0.001	85.2%	0.511
	Community	4	1.88	1.04	3.38	0.274	22.8%	
Study population	Pregnant women	9	1.66	1.10	2.51	<0.001	80.8%	0.646
	Post partum or pregnant	1	1.18	0.29	4.84			
Country	Burundi	1	1.79	0.62	5.16			0.1791
	Central African Republic	1	0.68	0.23	2.02			
	Democratic Republic of Congo	1	0.79	0.25	2.48			
	Ethiopia	1	1.63	0.51	5.17			
	Kenya	1	1.04	0.27	3.99			
	Rwanda	2	4.86	1.92	12.35	0.02	81.2%	
	South Sudan	1	1.18	0.30	4.57			
	Tanzania	2	1.94	0.90	4.17	0.100	63.0%	
Study quality	Good	4	1.79	0.96	3.32	0.210	33.8%	0.688
	Moderate-to-low	6	1.51	0.90	2.55	<0.001	85.2%	

Abbreviations: CI: confidence interval. ITN: insecticide treated net.
†p-value Q-statistic (assessment of heterogeneity).

Figure S2.17: **Marital status and ITN use: Married vs. not married**

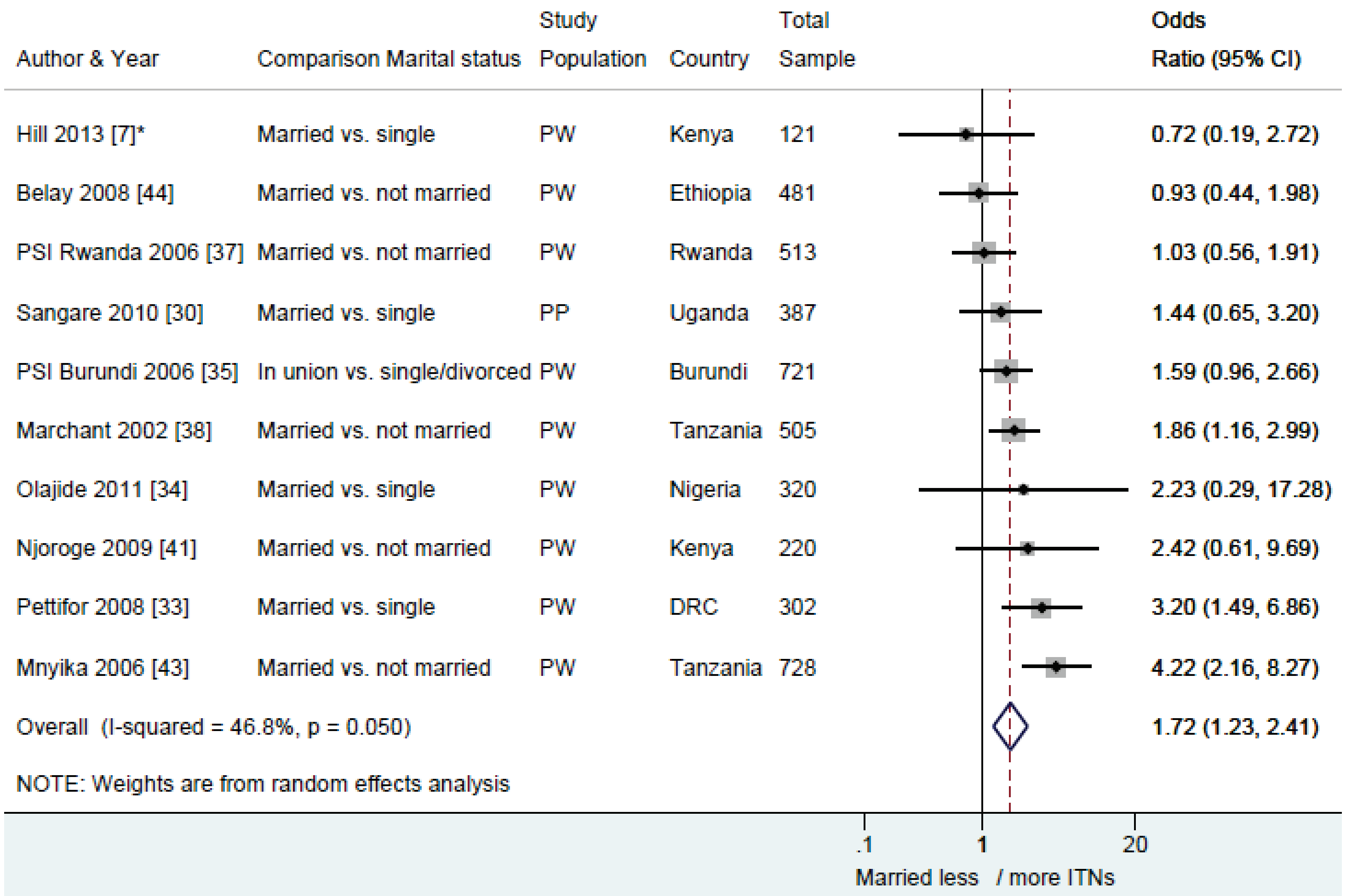

Abbreviations: CI: confidence interval. DRC: Democratic Republic of Congo. ITN: insecticide treated net. PP: postpartum women. PW: pregnant women.
The grey boxes indicate the weight for each study.
*Studies using a cluster design. The study by Hill 2013 took the study design into account in the analysis [7].
Summary estimate: p =0.001. I^2 =47%, 95% CI 0-74%. Total population: 4298 women in 10 studies.
Notes: Sangare 2010 & Belay 2008: Net/ITN use vs. none use among net/ITN owners, respectively [30,44].
PSI Rwanda 2006 and PSI Burundi 2006 may have used cluster design; this is not clear from the reports [35,37].

***Table S2.17*: Marital status and ITN use sub-group analyses: Married vs. not married**

						Within subgroups		Between subgroups
**Covariate**	**Subgroup**	**N studies**	**Odds Ratio**	**Lower limit 95% CI**	**Upper limit 95% CI**	**p-value†**	**Within group I^2**	**p-value†**
Type of net	ITN	7	1.40	1.03	1.91	0.487	0.0%	**0.015**
	Net	3	2.82	1.76	4.52	0.121	52.5%	
Net use when	Last night	5	1.38	0.90	2.11	0.100	48.6%	0.123
	Pregnancy	5	2.27	1.42	3.61	0.268	23.0%	
Location enrolment	Clinic	4	3.47	2.19	5.51	0.847	0.0%	**0.001**
	Community	6	1.39	1.07	1.80	0.457	0.0%	
Study population	Pregnant women	9	1.76	1.21	2.55	0.033	52.1%	0.773
	Post partum women	1	1.44	0.48	4.34			
Country	Burundi	1	1.59	0.53	4.80			0.750
	Democratic Republic of Congo	1	3.20	0.93	11.06			
	Ethiopia	1	0.93	0.27	3.20			
	Kenya	2	1.30	0.40	4.23	0.216	34.7%	
	Nigeria	1	2.23	0.23	21.55			
	Rwanda	1	1.03	0.32	3.27			
	Tanzania	2	2.70	1.21	6.02	0.051	73.8%	
	Uganda	1	1.44	0.41	5.08			
Study quality	Good	6	1.27	0.94	1.72	0.627	0.0%	0.002
	Moderate-to-low	4	2.57	1.83	3.61	0.239	28.9%	

Abbreviations: CI: confidence interval. ITN: insecticide treated net.
†p-value Q-statistic (assessment of heterogeneity).

Figure S2.18: **Age and ITN use: older vs. younger age groups**

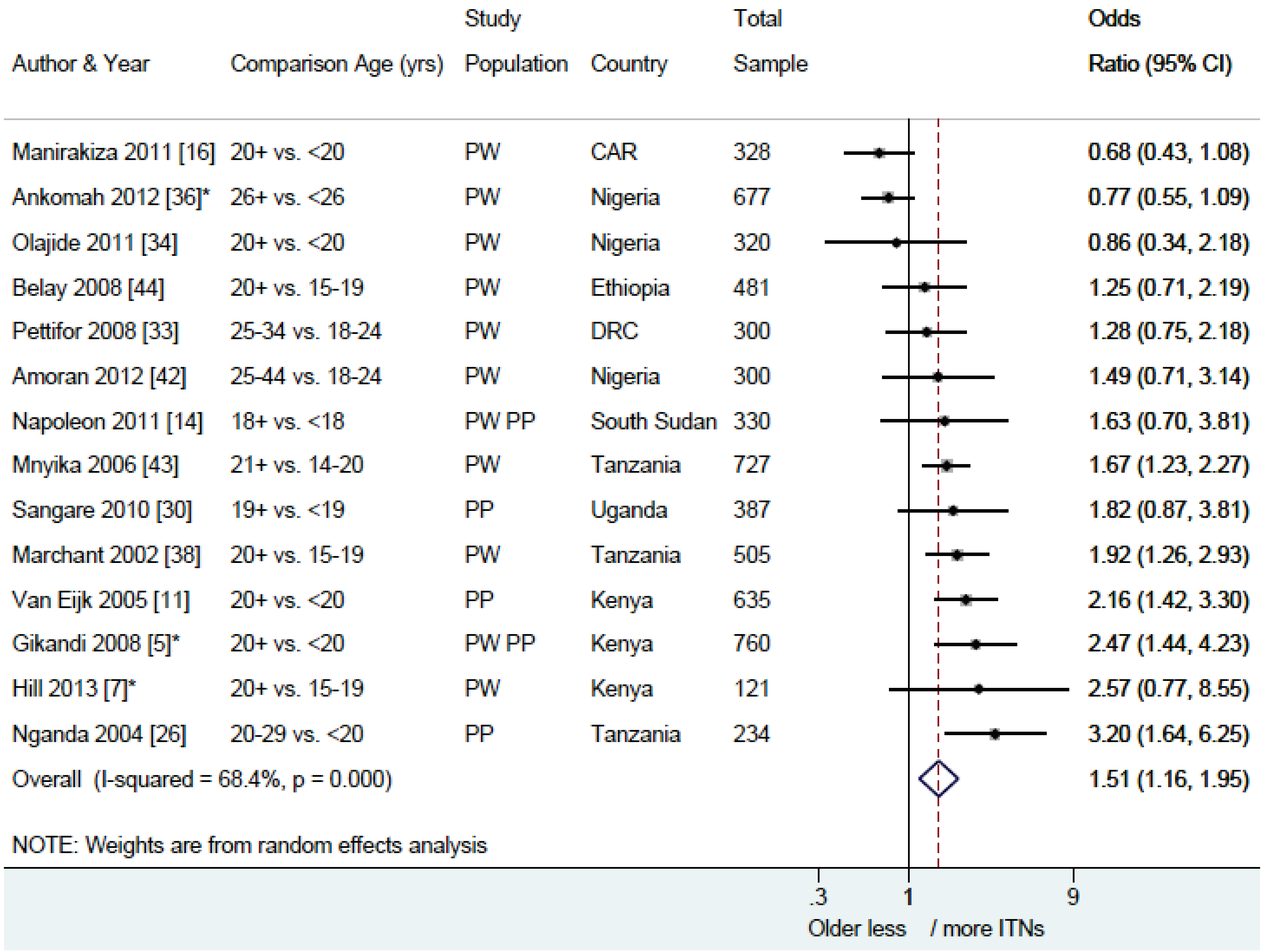

Abbreviations: CAR: Central African Republic. CI: confidence interval. DRC: Democratic Republic of Congo. ITN: insecticide treated net. OR: PP: postpartum women. PW: pregnant women. Yrs: years. The grey boxes indicate the weight for each study.

*Studies using a cluster design. The studies by Gikandi 2008, and Hill 2013 took the study design into account in the analysis [5,7]. For Ankomah 2012 this was not clear [36].

Summary estimate: p=0.002. I^2 =68%, 95% CI 45-82%. Total population: 6105 women in 14 studies.
Notes: Sangare 2010 & Belay 2008: Net/ITN use vs. none use among net/ITN owners, respectively [30,44].

***Table S2.18*: Age and ITN use sub-group analyses: older age groups vs. younger age groups**

						Within subgroups		Between subgroups
**Covariate**	**Subgroup**	**N studies**	**Odds Ratio**	**Lower limit 95% CI**	**Upper limit 95% CI**	**p-value†**	**Within group I^2**	**p-value†**
Type of net	ITN	11	1.49	1.09	2.04	<0.001	74.7%	0.884
	Net	3	1.57	0.89	2.76	0.649	0.0%	
Net use when	Last night	4	1.14	0.74	1.78	0.113	49.8%	0.156
	Pregnancy	10	1.67	1.26	2.20	0.004	62.8%	
Location enrolment	Clinic	7	1.38	0.93	2.04	0.007	66.4%	0.532
	Community	7	1.64	1.12	2.41	0.001	74.0%	
Study population	Pregnant women	9	1.22	0.93	1.61	0.003	66.2%	**0.043**
	Post partum women	3	2.32	1.42	3.79	0.497	0.0%	
	Pregnant and postpartum	2	2.11	1.12	4.00	0.419	0.0%	
Country	Central African Republic	1	0.68	0.43	1.08			**<0.001**
	Democratic Republic of Congo	1	1.28	0.75	2.18			
	Ethiopia	1	1.25	0.71	2.19			
	Kenya	3	2.30	1.67	3.16	0.913	0.0%	
	Nigeria	3	0.88	0.64	1.17	0.290	19.3%	
	South Sudan	1	1.63	0.70	3.81			
	Tanzania	3	1.89	1.49	2.38	0.224	33.2%	
	Uganda	1	1.82	0.87	3.81			
Study quality	Good	4	1.24	0.77	1.99	0.054	60.8	0.350
	Moderate-to-low	10	1.61	1.21	2.14	0.003	64.0	

Abbreviations: CI: confidence interval. ITN: Insecticide treated net.
†p-value Q-statistic (assessment of heterogeneity).

Figure S2.19: **Parity/gravidity and ITN use: Higher parity or gravidity number vs. lower**

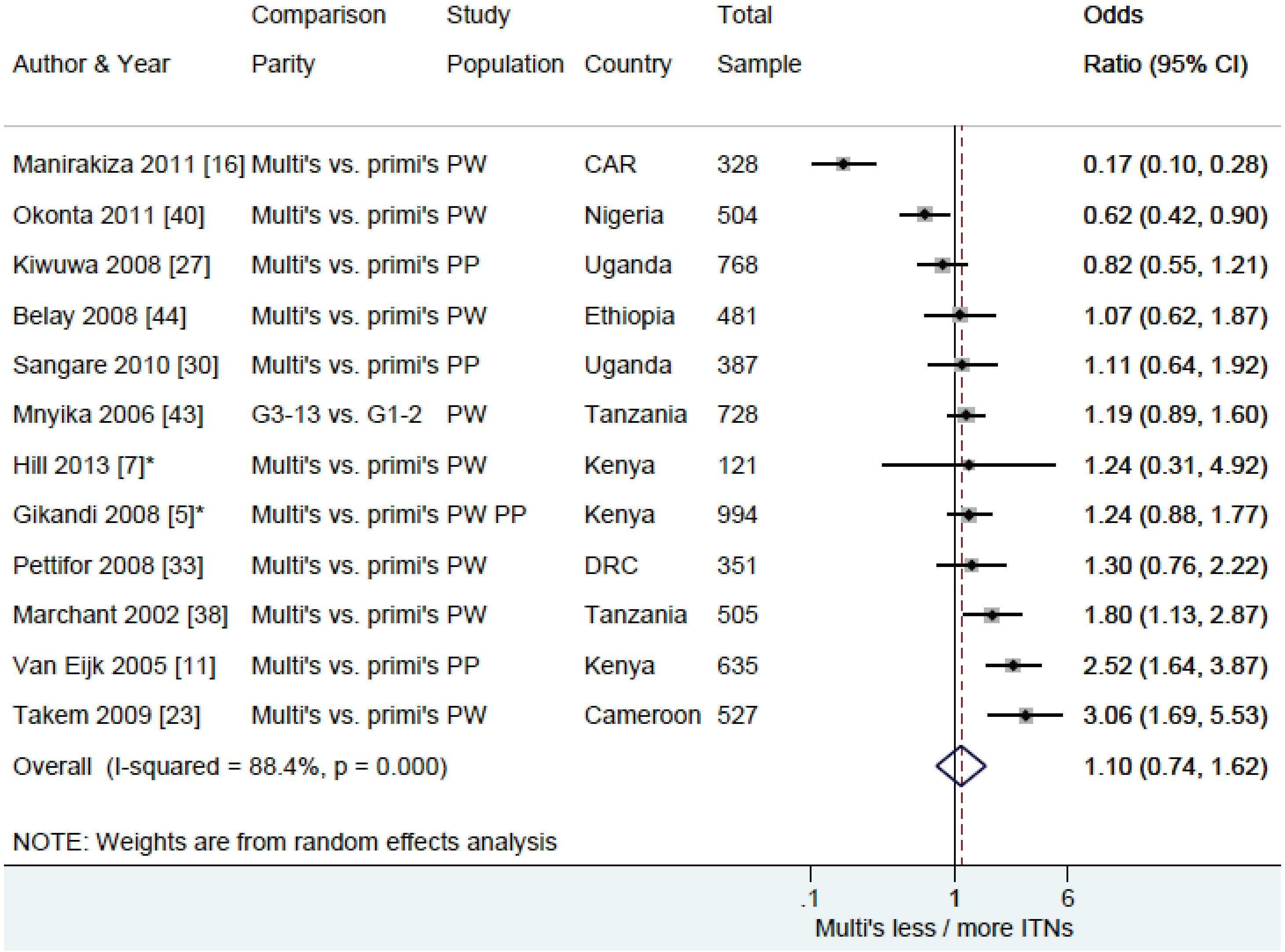

Abbreviations: CAR: Central African Republic. CI: confidence interval. DRC: Democratic Republic of Congo. G: gravidae. ITN: insecticide treated net. Multi's: multigravidae (pregnant women) or multiparae (postpartum women). Primi's: primigravidae (pregnant women) or primiparae (postpartum women). PP: postpartum women. PW: pregnant women. The grey boxes indicate the weight for each study.

*Studies using a cluster design. The studies by Gikandi 2008, and Hill 2013 took the study design into account in the analysis [5,7].

Summary estimate: p =0.641. I^2 =88%, 95% CI 82-93%**.** Total population: 6329 women in 12 studies.
Notes: Sangare 2010 & Belay 2008: Net/ITN use vs. none use among net/ITN owners, respectively [30,44].

***Table S2.19*: Parity/gravidity and ITN use sub group analysis: Higher parity or gravidity number vs. lower**

						**Within subgroups**		**Between subgroups**
**Covariate**	**Subgroup**	**N studies**	**Odds Ratio**	**Lower limit 95% CI**	**Upper limit 95% CI**	**p-value†**	**Within group I^2**	**p-value†**
Type of net	ITN	8	1.10	0.66	1.86	<0.001	92.4%	0.972
	Net	4	1.09	0.54	2.21	0.426	0.0%	
Net use when	Last night	3	1.19	0.50	2.88	0.887	0.0%	0.833
	Pregnancy	9	1.07	0.68	1.70	<0.001	91.5%	
Location enrolment	Clinic	5	0.86	0.47	1.56	<0.001	94.1%	0.285
	Community	7	1.32	0.79	2.24	0.009	64.8%	
Study population	Pregnant women	8	1.00	0.58	1.73	<0.001	90.8%	0.858
	Post partum women	3	1.32	0.56	3.13	0.001	86.4%	
	Pregnant and postpartum	1	1.24	0.29	5.39			
Country	Cameroon	1	3.06	1.37	6.82			**<0.001**
	Central African Republic	1	0.17	0.08	0.35			
	Democratic Republic of Congo	1	1.30	0.61	2.78			
	Ethiopia	1	1.07	0.50	2.33			
	Kenya	3	1.67	1.07	2.62	0.042	68.5%	
	Nigeria	1	0.62	0.32	1.20			
	Tanzania	2	1.42	0.89	2.26	0.139	54.4%	
	Uganda	2	0.94	0.57	1.55	0.373	0.0%	
Study quality	Good	4	1.02	0.49	2.16	0.755	0.0%	0.826
	Moderate-to-low	8	1.13	0.69	1.85	<0.001	92.4%	

Abbreviations: CI: confidence interval. DRC: Democratic Republic of Congo. ITN: Insecticide treated net.
†p-value Q-statistic (assessment of heterogeneity).

***Figure S2.20*: Knowledge of malaria and ITN use: Knowledge on malaria/ITNs vs. no knowledge**

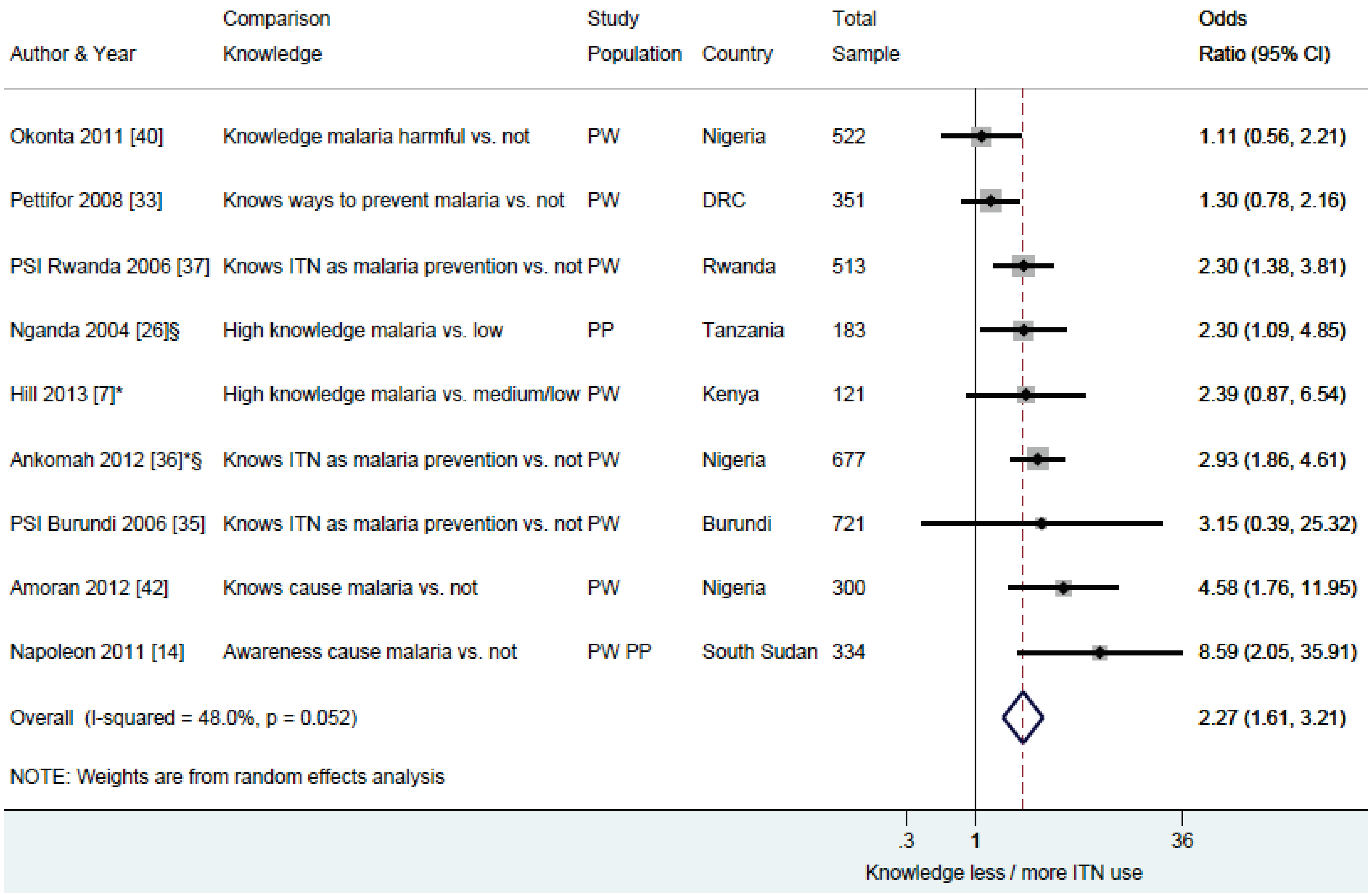

Abbreviations: CI: confidence interval. DRC: Democratic Republic of Congo. ITN: insecticide treated net. PP: postpartum women. PW: pregnant women. The grey boxes indicate the weight for each study.

*Studies using a cluster design. The study by Hill 2013 took the study design into account in the analysis [7]. For Ankomah 2012 this was not clear [36].

§ Studies where an adjusted OR was used

Summary estimate: p <0.001. I^2 =48%, 95% CI 0-76%. Total population: 3722 women in 9 studies.
Notes: PSI Rwanda 2006 and PSI Burundi 2006 may have used cluster design; this is not clear from the reports [35,37].

***Table S2.20*: Knowledge of malaria and ITN use sub-group analyses: Knowledge on malaria/ITNs vs. no knowledge**

						**Within subgroups**		**Between subgroups**
**Covariate**	**Subgroup**	**N studies**	**Odds Ratio**	**Lower limit 95% CI**	**Upper limit 95% CI**	**p-value†**	**Within group I^2**	**p-value†**
Type of net	ITN	8	2.51	1.79	3.53	0.166	32.8%	0.109
	Net	1	1.30	0.63	2.70			
Net use when	Last night	5	2.16	1.34	3.48	0.213	31.3%	0.679
	Pregnancy	4	2.54	1.40	4.61	0.023	68.5%	
Location enrolment	Clinic	5	2.03	1.28	3.24	0.016	67.1%	0.491
	Community	4	2.59	1.56	4.32	0.906	0.0%	
Study population	Pregnant women	7	2.10	1.45	3.04	0.069	48.7%	0.231
	Post partum women	1	2.30	0.86	6.19			
	Post partum or pregnant	1	8.59	1.79	41.32			
Country	Burundi	1	3.15	0.30	33.19			0.813
	Democratic Republic of Congo	1	1.30	0.39	4.35			
	Kenya	1	2.39	0.54	10.59			
	Nigeria	3	2.38	1.13	5.04	0.026	72.7%	
	Rwanda	1	2.30	0.69	7.68			
	South Sudan	1	8.59	1.42	52.09			
	Tanzania	1	2.30	0.61	8.67			
Study quality	Good	4	2.59	1.56	4.32	0.906	0.0%	0.491
	Moderate-to-low	5	2.03	1.28	3.24	0.016	67.1%	

Abbreviations: CI: confidence interval. ITN: insecticide treated nets.
†p-value Q-statistic (assessment of heterogeneity).

***Figure S2.21*: Other malaria prevention and ITN use: one or more doses of SP vs. none**

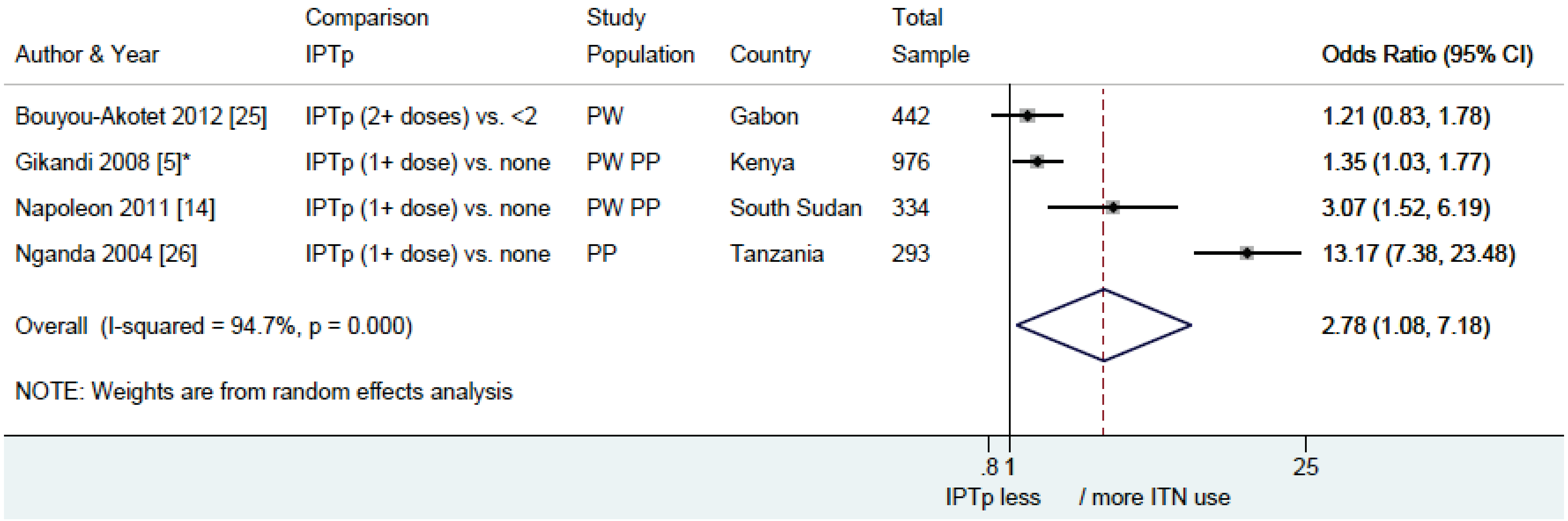

Abbreviations: CI: confidence interval.IPT: intermittent preventive treatment. ITN: insecticide treated net. PP: postpartum women. PW: pregnant women. SP: Sulfadoxine-pyrimethamine. The grey boxes indicate the weight for each study.
*Study with a cluster design. The study by Gikandi 2008 took the study design into account in the analysis [5]. § Studies where an adjusted OR was used
Summary estimate: p =0.035. I^2 =95%, 95% CI 89-97%. Total population: 2045 women in 4 studies.

***Table S2.21*: Other malaria prevention and ITN use subgroup analysis: SP dose(s) vs. none**

						Within subgroups		Between subgroups
**Covariate**	**Subgroup**	**N studies**	**Odds Ratio**	**Lower limit 95% CI**	**Upper limit 95% CI**	**p-value†**	**Within group I^2**	**p-value†**
Type of net	ITN	3	3.75	0.88	15.96	<0.001	96.0%	0.441
	Net	1	1.21	0.10	14.47			
Net use when	All studies assessed during pregnancy							
Location enrolment	Clinic	3	3.63	0.81	16.23	<0.001	96.6%	0.512
	Community	1	1.35	0.11	17.24			
Study population	Pregnant women	1	1.21	0.41	3.56			0.007
	Postpartum women	1	13.17	4.12	42.12			
	Post partum or pregnant	2	1.91	0.86	4.22	0.032	78.3%	
Country	Studies in 4 different countries							
Study quality	Good	1	1.21	0.10	14.47			0.441
	Moderate-to-low	3	3.75	0.88	15.96	<0.001	96.0%	Same division as net

Abbreviations: CI: Confidence interval. ITN: Insecticide treated net. SP: sulfadoxine-pyrimethamine †p-value Q-statistic (assessment of heterogeneity).

References

1. Sangare LR, Stergachis A, Brentlinger PE, Richardson BA, Staedke SG, et al. (2010) Determinants of use of intermittent preventive treatment of malaria in pregnancy: Jinja, Uganda. PLoS One 5: e15066.
2. Marchant T, Nathan R, Jones C, Mponda H, Bruce J, et al. (2008) Individual, facility and policy level influences on national coverage estimates for intermittent preventive treatment of malaria in pregnancy in Tanzania. Malar J 7: 260.
3. Mbonye AK, Hansen KS, Wamono F, Magnussen P (2010) Integration of malaria and HIV/AIDS prevention through the private sector in Uganda. Int Health 2: 52-58.
4. Zere E, Kirigia JM, Duale S, Akazili J (2012) Inequities in maternal and child health outcomes and interventions in Ghana. BMC Public Health 12: 252.
5. Gikandi PW, Noor AM, Gitonga CW, Ajanga AA, Snow RW (2008) Access and barriers to measures targeted to prevent malaria in pregnancy in rural Kenya. Trop Med Int Health 13: 208-217.
6. Gies S, Coulibaly SO, Ky C, Ouattara FT, Brabin BJ, et al. (2009) Community-based promotional campaign to improve uptake of intermittent preventive antimalarial treatment in pregnancy in Burkina Faso. Am J Trop Med Hyg 80: 460-469.
7. Hill J, Dellicour S, Bruce J, Ouma PO, Smedley J, et al. (2013) Effectiveness of antenatal clinics to deliver intermittent preventive treatment and insecticide treated nets for the control of malaria in pregnancy in Kenya. PLoS One In press.
8. Faye A, Manga NM, Seck I, Niang K, Leye MM, et al. (2012) Access to intermittent preventive treatment (IPT) in a situation of abolition of user's fee: role of economic welfare. Bull Soc Pathol Exot 105: 215-219.
9. De Allegri M, Louis VR, Tiendrebeogo J, Souares A, Ye M, et al. (2013) Moving towards universal coverage with malaria control interventions: achievements and challenges in rural Burkina Faso. Int J Health Plann Manage 28: 102-121.
10. Antwi GD (2010) Factors influencing the uptake of intermittent preventive treatment of malaria in pregnancy in Bosomtwe district of Ghana. Kumasi, Ghana: Kwami Nkrumah University of Science and Technology.
11. van Eijk AM, Blokland IE, Slutsker L, Odhiambo F, Ayisi JG, et al. (2005) Use of intermittent preventive treatment for malaria in pregnancy in a rural area of western Kenya with high coverage of insecticide-treated bed nets. Trop Med Int Health 10: 1134-1140.
12. Klebi GW (2009) Factors accounting for the decline in IPTp2/IPTp3 among pregnant mothers in the Keta municipality. Kumasi, Ghana: Kwami Nkrumah University of Science and Technology.
13. Amoran OE, Ariba AA, Iyaniwura CA (2012) Determinants of intermittent preventive treatment of malaria during pregnancy (IPTp) utilization in a rural town in Western Nigeria. Reprod Health 9: 12.
14. Napoleon RP, Anyangu AS, Omolocan J, Ongus JR (2011) Preventing malaria during pregnancy: factors determining the use of insecticide-treated bednets and intermittent preventive therapy in Juba. South Sudan Med J 4: 33-38.
15. Ouma PO, van Eijk AM, Hamel MJ, Sikuku E, Odhiambo F, et al. (2007) The effect of health care worker training on the use of intermittent preventive treatment for malaria in pregnancy in rural western Kenya. Trop Med Int Health 12: 953-961.
16. Manirakiza A, Serdouma E, Djalle D, Soula G, Laganier R, et al. (2011) Relatively low prevalence of peripheral and placental Plasmodium infection at delivery in Bangui, Central African Republic. J Trop Medicine 2011: 434816.
17. Iliyasu Z, Gajida AU, Galadanci HS, Abubakar IS, Baba AS, et al. (2012) Adherence to intermittent preventive treatment for malaria in pregnancy in urban Kano, northern Nigeria. Pathog Glob Health 106: 323-329.
18. Namusoke F, Ntale M, Wahlgren M, Kironde F, Mirembe F (2012) Validity of self-reported use of sulphadoxine-pyrimethamine intermittent presumptive treatment during pregnancy (IPTp): a cross-sectional study. Malar J 11: 310.
19. Onoka CA, Hanson K, Onwujekwe OE (2012) Low coverage of intermittent preventive treatment for malaria in pregnancy in Nigeria: demand-side influences. Malar J 11: 82.
20. Sande JH, Kaseje D, Nyapada L, Owino VO (2010) Fear of being tested for HIV at ANC clinics associated with low uptake of intermittent preventive treatment (IPT) of malaria among pregnant women attending Bondo District Hospital, Western Kenya. East Afr J Public Health 7: 92-96.
21. Guyatt HL, Noor AM, Ochola SA, Snow RW (2004) Use of intermittent presumptive treatment and insecticide treated bed nets by pregnant women in four Kenyan districts. Trop Med Int Health 9: 255-261.

22. d'Almeida TC, Agboton-Zoumenou MA, Garcia A, Massougbodji A, Briand V, et al. (2011) Field evaluation of the intermittent preventive treatment of malaria during pregnancy (IPTp) in Benin: evolution of the coverage rate since its implementation. Parasit Vectors 4: 108.
23. Takem EN, Achidi EA, Ndumbe PM (2009) Use of intermittent preventive treatment for malaria by pregnant women in Buea, Cameroon. Acta Trop 112: 54-58.
24. Adjei DJD (2009) Factors affecting the intermittent preventive therapy of malaria in pregnancy programme in the Ejisu-Juabeng municipality. Kumasi, Ghana: Kwame Nkrumah University of Science and Technology.
25. Bouyou-Akotet MK, Mawili-Mboumba DP, Kombila M (2013) Antenatal care visit attendance, intermittent preventive treatment and bed net use during pregnancy in Gabon. BMC Pregnancy Childbirth 13: 52.
26. Nganda RY, Drakeley C, Reyburn H, Marchant T (2004) Knowledge of malaria influences the use of insecticide treated nets but not intermittent presumptive treatment by pregnant women in Tanzania. Malar J 3: 42.
27. Kiwuwa MS, Mufubenga P (2008) Use of antenatal care, maternity services, intermittent presumptive treatment and insecticide treated bed nets by pregnant women in Luwero district, Uganda. Malar J 7: 44.
28. Ndyomugyenyi R, Katamanywa J (2010) Intermittent preventive treatment of malaria in pregnancy (IPTp): do frequent antenatal care visits ensure access and compliance to IPTp in Ugandan rural communities? Trans R Soc Trop Med Hyg 104: 536-540.
29. Nduka FO, Nwosu E, Oguariri RM (2011) Evaluation of the effectiveness and compliance of intermittent preventive treatment (IPT) in the control of malaria in pregnant women in south eastern Nigeria. Ann Trop Med Parasitol 105: 599-605.
30. Sangare LR (2010) Use of malaria prevention and control measures during pregnancy in Jinja, Uganda. Seattle, USA: University of Washington.
31. Auta A (2012) Demographic factors associated with insecticide treated net use among Nigerian women and children. N Am J Med Sci 4: 40-44.
32. Hassan SEH, Malik EM, Okoued SL, Eltayeb EM (2008) Retention and efficacy of long-lasting insecticide-treated nets distributed in eastern Sudan: a two-step community-based study. Malar J 7: 85.
33. Pettifor AE, Taylor E, Nku D, Duvall S, Tabala M, et al. (2008) Bed net ownership, use and perceptions among women seeking antenatal care in Kinshasa, Democratic Republic of the Congo (DRC): Opportunities for improved maternal and child health. BMC Public Health 8: 331.
34. Olajide FO, Afolabi OT, Olajide AO, Omisore AG, Omomuniniyi OA (2011) Challenges with the use of insecticide treated nets among pregnant women in Ife-Ijesha Zone, South Western Nigeria. J Comm Med Prim Health Care 23: 79-86.
35. PSI Burundi (2006) Determinants of the utilisation of nets among mothers with children less than years of age and pregnant women in Burundi. Bujumbura, Burundi.
36. Ankomah A, Adebayo SB, Arogundade ED, Anyanti J, Nwokolo E, et al. (2012) Determinants of insecticide-treated net ownership and utilization among pregnant women in Nigeria. BMC Public Health 12: 105.
37. PSI Rwanda (2006) Malaria TRaC Study evaluating the use of insecticide treated nets among pregnant women and children under 5 years of age. First Round. Kigali, Rwanda.
38. Marchant T, Schellenberg JA, Edgar T, Nathan R, Abdulla S, et al. (2002) Socially marketed insecticide-treated nets improve malaria and anaemia in pregnancy in southern Tanzania. Trop Med Int Health 7: 149-158.
39. Van Geertruyden JP, Ntakirutimana D, Erhart A, Rwagacondo C, Kabano A, et al. (2005) Malaria infection among pregnant women attending antenatal clinics in six Rwandan districts. Trop Med Int Health 10: 681-688.
40. Okonta PI (2011) Utilization of insecticide-treated net during pregnancy in Delta-State: a survey of recently delivered mothers. Ebonyi Med J 10: 105-111.
41. Njoroge FK, Kimani VN, Ongore D, Akwale WS (2009) Use of insecticide treated bed nets among pregnant women in Kilifi District, Kenya. East Afr Med J 86: 314-322.
42. Amoran OE, Lawal KM, Jeminusi OA, Alabi AA, Oluwole FA (2012) Determinants of uptake of insecticide treated nets among pregnant women in Ado-Odo Local Government area of Ogun State, Nigeria. J Community Med Health Edu 2: 126.
43. Mnyika KS, Kabalimu TK, Mbaruku G (2006) Determinants of utilisation of mosquito bednets for malaria prevention among pregnant women in Kigoma urban district, western Tanzania. East Afr J of Public Health 3: 31-34.
44. Belay M, Deressa W (2008) Use of insecticide treated nets by pregnant women and associated factors in a pre-dominantly rural population in northern Ethiopia. Trop Med Int Health 13: 1303-1313.

45. Karunamoorthi K, Deboch B, Tafere Y (2010) Knowledge and practice concerning malaria, insecticide-treated net (ITN) utilization and antimalarial treatment among pregnant women attending specialist antenatal clinics. J Public Health 18: 559-566.
